# Alumanyl silanides as multifunctional reagents for olefin cycloaddition, CO hydrosilylation, and reductive CO coupling

**DOI:** 10.1039/d5sc09910b

**Published:** 2026-01-15

**Authors:** Moritz Ludwig, Johannes Voigtland, Petra Vasko, Sebastian Stigler, Shigeyoshi Inoue

**Affiliations:** a TUM School of Natural Sciences, Department of Chemistry, Catalysis Research Center and Institute for Silicon Chemistry, Technical University of Munich Lichtenbergstraße 4 85748 Garching bei München Germany s.inoue@tum.de; b Department of Chemistry, P.O. Box 55 (A.I. Virtasen Aukio 1), 00014 University of Helsinki Finland

## Abstract

Alumanyl silanides represent a rare class of main group complexes, characterized by an anionic Al–Si bond, stabilized through an intimate ion pair. Computations revealed pronounced multiple-bond character in these alumanyl silanides, which is further enhanced upon counter ion sequestration. Despite these electronic features, such bonding motifs remain largely unexplored in experimental chemistry. In this work, we investigate the reactivity of a sodium alumanyl silanide, stabilized by bulky silyl groups and an N-heterocyclic imine (NHI), towards alkenes, alkynes, aldehydes, CO_2_, and CO. The addition of ethylene, styrene, or mesitylaldehyde to said alumanyl silanide affords the corresponding [2+2]-cycloaddition products, characterized by polarized Al–Si–C–C and Al–Si–C–O heterocycles, respectively. Furthermore, the investigated alumanyl silanide captures two equivalents of CO_2_. One molecule is inserted between the Al center and its adjacent NHI ligand, whereas a second molecule of CO_2_ adds across the central Al–Si bond. Moreover, the title compound selectively adds one equivalent of CO to the Al–Si core. This is followed by 1,2-hydrogen migration from the silicon center to the carbon in the formed Al–Si–C–O cycle, showcasing a rare main group-mediated hydrosilylation of CO. The mechanism for this formation is examined using DFT calculations, which reveal the generation of a cyclic carbene intermediate as the key step. At low temperatures, the intermediate is successfully trapped in the presence of additional CO and an N-heterocyclic carbene (NHC), yielding silanyl ethynolates *via* reductive coupling of two CO molecules.

## Abstract 2

This article reports on the reactivity of a previously described alumanyl silanide with a series of olefins, carbonyls, and CO_2_ by means of [2+2]-cycloaddition reactions in addition to the formal hydrosilylation of CO.

## Graphical abstract

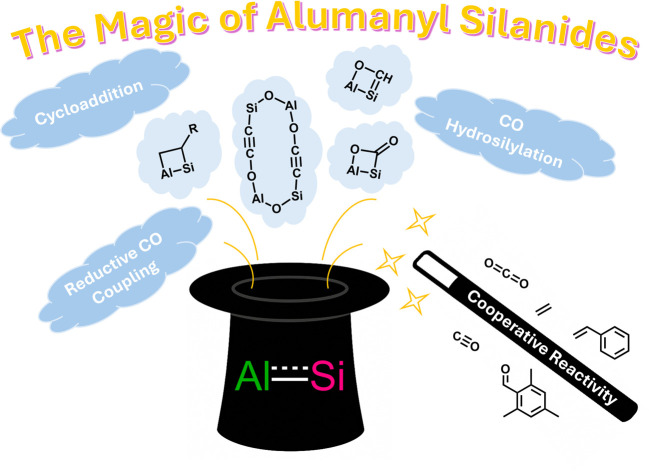

## Introduction

The generation of C–C bonds from carbon monoxide as a C1 feedstock is of fundamental importance for large-scale industrial processes, such as the Fischer–Tropsch, Monsanto, or Cativa processes.^1^ To efficiently break the stable C≡O bond, these processes rely on transition-metal-based catalysts that enable low-energy reaction pathways but are often associated with toxicity and high procurement cost.^2^ In recent years, more earth-abundant and environmentally friendly low-valent s- and p-block elements have shown transition-metal-like reactivity in the activation and transformation of carbon monoxide.^3^

**Scheme 1:**
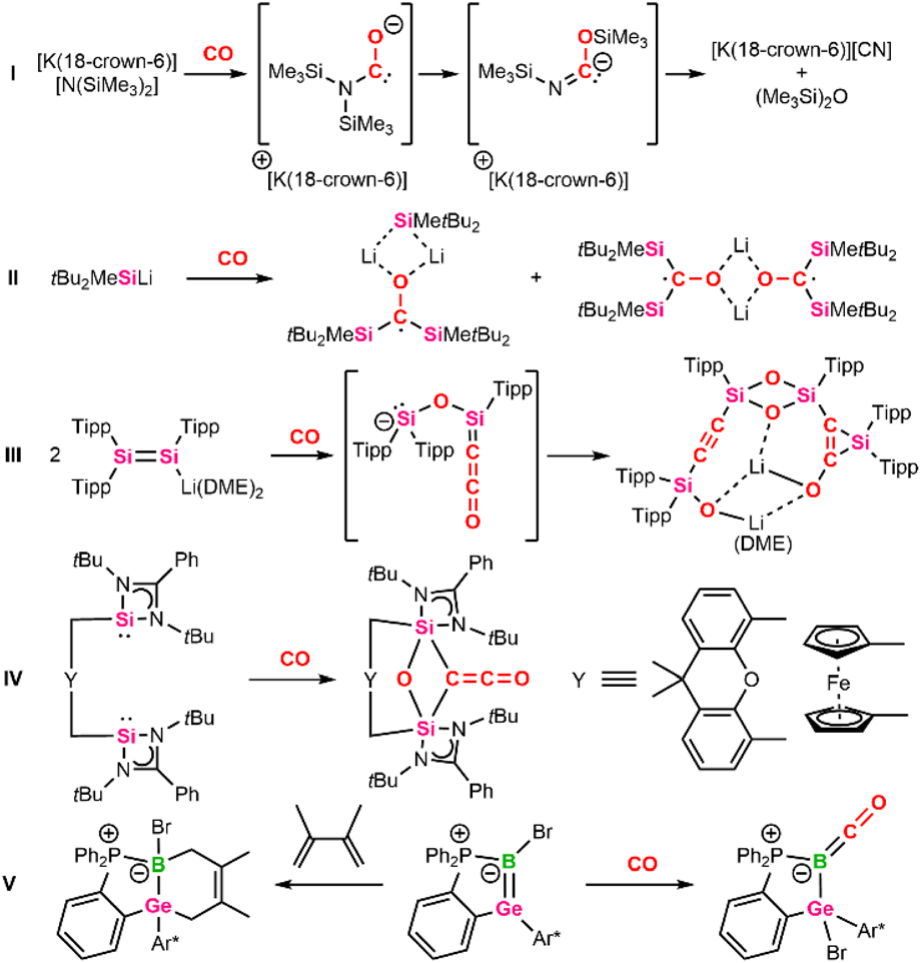
Selected examples of main-group mediated CO activation (Tipp = 2,4,6-iPr_3_C_6_H_3_, Ar* = 2,6-Tipp_2_C_6_H_3,_ Dipp = 2,6-iPr_3_C_6_H_3_, DME = dimethoxyethane).

The first report using molten potassium for CO coupling dates back to the 1830s.^4^ Since then, group 1 metals (Li, Na, K)^5^ have been investigated for CO homologation *via* C–C coupling. Modern approaches are based on related organometallic reducing agents, such as benzyl potassium,^6^ dilithiomethane,^7^ alkali metal amides^8,9^ and phosphides.^10,11^ Potassium and lithium silylamides have even enabled complete cleavage of the CO bond, resulting in the formation of cyanides and hexamethyldisiloxane (Scheme 1, I).^12^ According to calculations, key step for this reaction was the formation of a transient carbene after nucleophilic attack at CO and subsequent 1,3-silyl migration. Since then, further systems, relying on the synergistic effect of alkali metals and p-block elements such as boryl lithium^13,14^ or simple silanides,^15,16^ have been reported to insert CO in the highly polarized B–Li or Si–Li bond (Scheme 1, II).^17^ These findings complement the well-established reactivity of classical organolithium compounds.^18^ Furthermore, systems based on a cooperative effect of multiple reactive centers, including low-valent dimeric^3,19–26^ or multiple-bonded main-group compounds^27–30^ and frustrated Lewis pairs (FLPs),^31^ enable CO activation.^32^ Noteworthy is the reductive coupling of CO by a disilenide, characterized by an anionic [Si=Si]^−^ center and a lithium counter ion, yielding an alkynyl silane (Scheme 1, III).^33^ Said silane is assumed to be formed *via* a ketenyl intermediate. Elsewhere, the selective formation of ketenes (C=C=O) from two CO molecules using bis-N-heterocyclic silylenes (NHSi) was reported (Scheme 1, IV).^34^ Here, the NHSi units were bridged *via* a xanthene or ferrocene spacer ligand to control the distance between the active Si(ii) centers and to incorporate two carbonyl moieties in between.

**Scheme 2:**
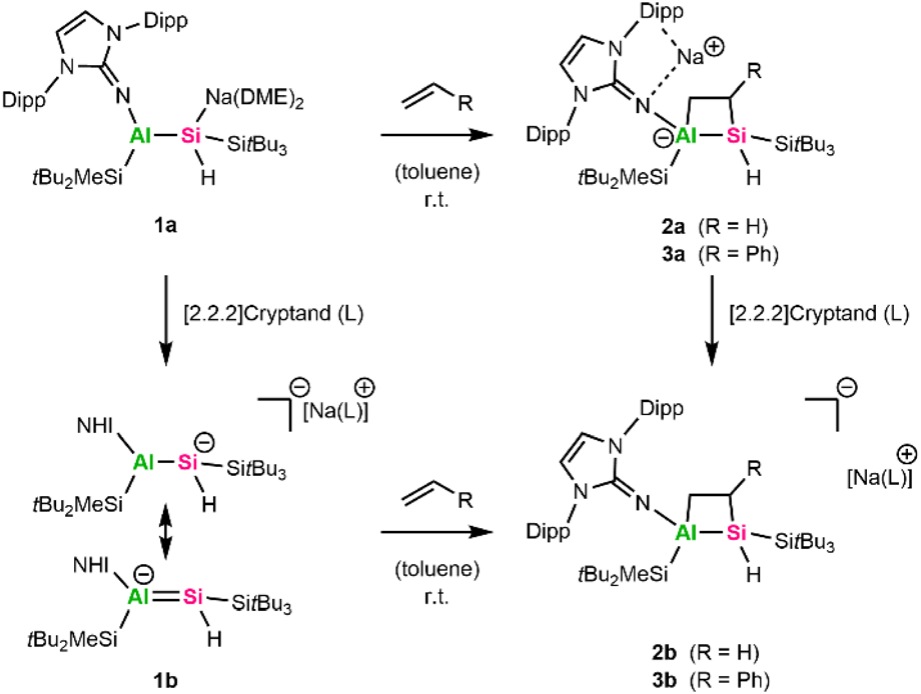
Synthesis of 2a, 2b, 3a and 3b (Dipp = 2,6-iPr_3_C_6_H_3_).

In 2023, we isolated an alumanyl silanide 1a (Scheme 2) featuring an Al–Si multiple bond, stabilized by bulky silyl groups, an NHI, and a Si–Na contact ion pair.^35^ The Al–Si bond in 1a is highly polarized toward silicon, as shown by NMR and bonding analyses. Correspondingly, 1a reacts as a silicon-centered nucleophile in the presence of TMSI or phenylacetylene and enables salt metathesis with NHC-stabilized coinage metal halides to form alumanyl silyl coinage metal complexes.^36^ When the sodium counter ion of 1a was sequestered, the multiple bond character was enhanced, emphasizing its aluminata-silene resonance structure 1b marked by an anionic [Al=Si]^−^ core (Scheme 2).^35^ Apart from a proposed transient intermediate with multiple-bonding between aluminum and silicon,^37^ there is only one other isolated aluminata-silene reported. This species exhibits a very short Al=Si bond and displays classic double bond reactivity, including the full oxidation of the Al=Si bond *via* double sulfur insertion and intramolecular C–H addition across the Al=Si bond at elevated temperatures.^38^ In contrast, we were not able to exploit the multiple-bond character in 1a/1b experimentally so far.

To date, only a few heterodiatomic complexes formed from heavier group 13 and group 14 elements are known, and their reactivity has hardly been investigated.^28,39,40^ Despite the rich chemistry of boraalkenes^41^ and borata-alkenes,^42^ which feature neutral or anionic B=C/[B=C]^−^ double bonds, the list of examples is limited to borasilenes (B=Si)^43,44^ and borata-silenes ([B=Si]^−^),^45^ neutral four-membered aromatic B_2_Si_2_ heterocycles,^46^ boragermenes (B=Ge),^43,47,48^ a zwitterionic stannaborene (B=Sn),^49^ heavier allenic anions of the type [>E_14_=E_13_=E_14_<]^−^ (E_13_ = Ga, In; E_14_ = Si, Ge)^50^ and an aluminate complex characterized by an anionic [Al=C]^−^ core.^51^ Very recently, our group published the first alumene, which is characterized by a neutral [Al=C] motif.^19^ The difficulty for the formation of multiple bonding, particularly between heavier group 13 and 14 elements, arises due to polarization of the E_13_–E_14_ (E = p-block element) bond and the intrinsic Lewis acidity of the triels in addition to weaker π-bonding due to poor orbital overlap when going down the group.^39,52^

It should be noted that the only in-depth reactivity study has been reported for a zwitterionic boragermene.^48^ Interestingly, said boragermene shows a versatile chemistry, including the addition of carbon-based Lewis acids (NHC, AdNC, CO) at the nucleophilic boron center to form B–C bonds (Scheme 1, V). In addition, the B=Ge double bond was accessed by [2+2]- and [2+4]-cycloaddition reactions with CO_2_ and dimethylbutadiene or partial bond oxidation with selenium to give the respective three-, four-, and six-membered heterocycles.^48^

Inspired by the reports on silicon-centered CO activation, we were interested in the capacity of 1a to activate CO based on its predominant silanide character.^15,27,33,34^ To further complement our earlier reactivity studies and highlight the reactivity dualism between π-bonding-type and zwitterionic reactivity (nucleophilic Si and electrophilic Al center), the partial multiple bond character of the central Al–Si bond is investigated. Here we report on the ability of 1a/1b to undergo [2+2]-cycloaddition reactions in the presence of olefins and the facile activation of C=O/C≡O bonds in mesitylaldehyde, CO_2,_ and CO by 1a.

## Results and discussion

### [2+2]-Cycloaddition with alkenes and alkynes

A typical reactivity observed not only for alkenes but also for their heavier analogues is the cycloaddition reaction across unsaturated carbon–carbon bonds. As previously discussed, [2+2]- and [2+4]-cycloaddition reactions have been well-documented for highly polarized heteroatomic double bonds.^48^ To further investigate the multiple-bonding character of alumanyl silanide 1a and aluminata-silene 1b, we were interested in their behavior towards unsaturated substrates. It should be noted that 1a possesses a sterically highly crowded aluminum center, limiting the choice of suitable substrates. As such, no reaction was observed for sterically encumbered substrates diphenylacetylene, *cis*-stilbene, *cis*- and *trans*-3-hexene, 1,5-cyclooctadiene, or norbornadiene, but rather a decomposition of the starting material 1a above 90 °C.

We, therefore, probed the reactivity of 1a with terminal unsaturated compounds, including neohexene, styrene, ethylene, and acetylene. While neohexene did not react with 1a even at elevated temperatures up to 90 °C, 1a was completely consumed in the presence of 1 bar acetylene, yet no selective products could be identified in the ^1^H NMR spectrum. Exposure of a C_6_D_6_ solution of 1a to 1 bar ethylene at room temperature results in complete discoloration of the orange color of 1a. The formal [2+2]-cycloaddition product 2a (Scheme 2) was obtained as an off-white solid in 72% yield after workup.

The ^1^H NMR spectrum of 2a shows four broad resonances around 2.72, 1.78, 0.96, and 0.43 ppm, corresponding to the diastereotopic protons from the two CH_2_-groups in the newly formed Al–Si–C–C ring. Furthermore, the ^1^H–^13^C HSQC NMR spectrum matches these proton resonances to carbon shifts at 7.3 and 18.4 ppm in the ^13^C NMR spectra.

The resonance of the Si-bonded proton overlaps with the signal of the Si^*t*^Bu_3_ group at 1.27 ppm (C_6_D_6_) in the ^1^H NMR spectrum and was assigned *via*^1^H–^29^Si HMBC experiments. The Si–H bond stretching was observed at 1973 cm^−1^ in the infrared spectrum, which aligns with the Si–H band of 1a (1920 cm^−1^).^35^^29^Si NMR spectroscopy reveals a significantly low-field shift to −82.3 ppm for the central Si–H group in comparison to 1a (−163.0 ppm), resulting from the change in the silicon coordination. For the substituted silyl groups, silicon shifts similar to the starting material (1a: 37.3/0.8 ppm Si^*t*^Bu_3_/Si^*t*^Bu_2_Me; 2a: 17.0/−0.2 ppm Si^*t*^Bu_3_/Si^*t*^Bu_2_Me) were observed.

**Fig. 1:**
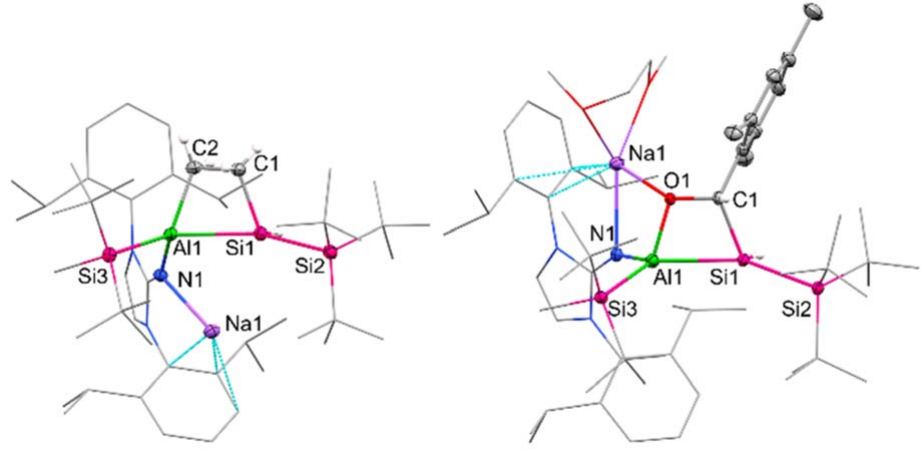
Molecular structure of 2a (left) and 5 (right) with thermal ellipsoids drawn at the 20% probability level. Hydrogen atoms are omitted for clarity, except on Si1 and C1/C2, respectively. The NHI ligand and selected alkyl/aryl groups are depicted as wireframes. Selected bond lengths [Å] and angles [°] of 5: Al1–Si1 = 2.5502(5), Al1–Si3 = 2.5502(5), Al1–O1 = 1.8125(8), O1–C1 = 1.4603(13), Si1–C1 = 1.9690(11), Si1–Si2 = 2.4032(4), O1–Al1–Si1 = 72.98(3), Al1–Si1–C1 = 71.10(3), Si1–C1–C2 = 100.76(6), C1–O1–Al1 = 108.84(6).

Single crystals of 2a were obtained by cooling a concentrated solution of a pentane/toluene mixture at −35 °C. SC-XRD clearly confirmed the connectivity of 2a (Fig. 1); however, bonding parameters are not discussed due to the limited data quality.

Thus far, [2+2]-cycloaddition reactions with ethylene are predominantly known for heavier group 13 and 14 compounds featuring homodiatomic multiple bonds, with only a few examples being reported for highly polarized heteroatomic multiple bonds.^53^ In the case of phosphaalumenes, complete cleavage of the Al=P double bond *via* the insertion of 2 equivalents of ethylene is reported, with the formation of an Al–P–C–C heterocyclic ring proposed as an intermediate, whereas reactions with sterically more hindering alkenes and alkynes result in the formation of typical [2+2]-cycloaddition products.^54^

In terms of molecular heterocycles containing Al–Si bonds, a few examples are known: besides the aforementioned aluminata-silene and its reaction products, Al_2_Si_2_ and AlSiC_3_N heterocycles were obtained from silylene-stabilized aluminylene derivatives either by dimerization or the addition of 2-methylquinoline.^37,38,55^ Furthermore, a six-membered Al_2_Si_6_ cycle was obtained by the reaction of a bis-silylene and an aluminum hydride.^56^ Adding to this, alumanyl silanide 1a allows for facile access to aluminum silicon heterocycles in moderate to good yields. Such four-membered heterocycles are prone to further functionalization based on the ring strain, adding to a polarized and weak Al–C single bond.^54,57^ Unexpectedly, 2a remains stable up to 120 °C in solution (C_6_D_6_ in a sealed NMR tube) and does not react with another ethylene molecule before unselective decomposition occurs.

The analogous [2+2]-cycloaddition product 3a (Scheme 2) was obtained in 62% yield when 1a was reacted with styrene at room temperature overnight, as apparent from multinuclear NMR spectroscopy. A doublet of doublets (dd) at 3.03 ppm (^2^*J*_Si–H_ = 10 Hz; ^3^*J*_H–H_ = 5 Hz) can be assigned to the Si–H group (^1^*J*_Si–H_ = 116 Hz) in the ^1^H- and ^1^H–^29^Si HMBC spectra. The CH group's proton of the newly formed Al–Si–C–C heterocycle overlaps with resonances of the Dipp group protons in the range of 1.52–1.43 ppm, but it can be assigned *via* a ^1^H–^29^Si-HMBC experiment. The endocyclic CH_2_ group, featuring two diastereotopic protons, gives rise to a broad singlet at 1.81 ppm and a doublet of doublets (dd) at 2.45 ppm (^3^*J*_HA–H_ = 5 Hz; ^3^*J*_HB–H_ = 15 Hz). The central Si atom in 3a resonates at −91.6 ppm in the ^29^Si NMR spectra and is in a similar range to the Si center in 2a (−82.3 ppm). To date, we have not been able to grow crystals of 3a suitable for SC-XRD.

For comparison and to investigate the influence of the sodium counter ion on the observed [2+2]-cycloaddition reactions, we repeated the experiments with aluminata-silene 1b, which has no contact counter cation and exhibits a more pronounced multiple bond character compared to 1a.^35^ Upon addition of ethylene or styrene to a solution of 1b in C_6_D_6_ at room temperature, an immediate discoloration is observed. Multinuclear NMR spectroscopy confirms the selective formation of the [2+2]-cycloaddition products 2b and 3b, respectively. The characteristic Si–H group can be observed around 3.09 ppm (^1^*J*_Si–H_ = 116 Hz) for 2b and 3.16 ppm (^1^*J*_Si–H_ = 123 Hz) for 3b in the ^1^H NMR spectrum and produces similar shifts in the ^29^Si spectra (−81.7 ppm/−91.2 ppm 2b/3b), respectively. Alternatively, 2b and 3b can be synthesized by sequestering the sodium counter cation in 2a and 3a by adding stoichiometric amounts of [2.2.2]-cryptand. Based on this, we can conclude that the role of the Na counter cation in the formation of the [2+2]-cycloaddition products is negligible. Attempts to reverse the discussed [2+2]-cycloadditions at elevated temperatures up to 120 °C and under vacuum were unsuccessful.

### [2+2]-Cycloaddition of mesitylaldehyde and CO_2_

In recent years, various bimetallic systems featuring an electron-rich Lewis base adjacent to a Lewis acidic center have been utilized for CO_2_ activation.^58^ Due to the strongly polarized Al–Si bond present in alumanyl silanide 1a, we estimated that the activation of other polarized bonds, such as C=O double bonds found in ketones, aldehydes, and CO_2_, would be feasible. Discoloration is observed when pressurizing a J-Young tube of 1a in C_6_D_6_ with 1 bar CO_2_ at room temperature, with a white precipitate forming within 30 min. The precipitate can be redissolved by adding small amounts of acetonitrile. Multinuclear NMR spectroscopy analysis of the resulting C_6_D_6_/MeCN-d_3_ mixture confirms the uptake of two CO_2_ molecules by 1a.

**Scheme 3:**
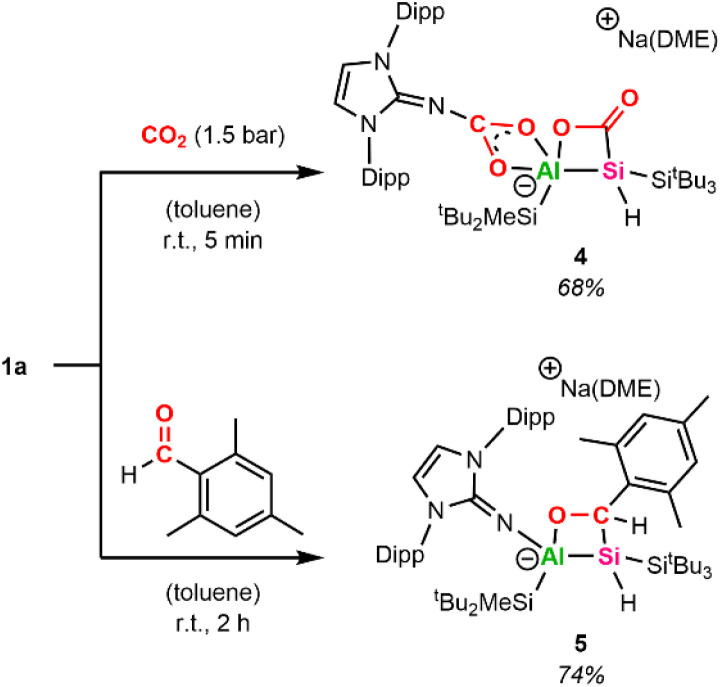
Synthesis of 4 and 5 (Dipp = 2,6-iPr_3_C_6_H_3_).

One molecule is inserted between the aluminum center and the NHI, while a second molecule adds across the central Al–Si bond, yielding the [2+2]-cycloaddition product 4 (Scheme 3). The proton bonded to the central Si gives a single resonance at 4.22 ppm in the ^1^H NMR spectrum, which lies within the typical region for supersilyl-substituted tetra-coordinated silanes.^59^^1^H–^13^C HMBC spectroscopy reveals a cross-peak of said proton with the carbon in the Al–Si–C–O heterocycle at *δ*_C_ = 188.4 ppm. Similar shifts in ^13^C NMR spectra have been observed for analogous cycloaddition products of disilene (185.9 ppm)^60^ and germaborene (184.2 ppm).^48^ In contrast to the germaborene, which is polarized towards the boron center, the carbon of CO_2_ adds to the more electron-rich silicon center in 1a. As a result, the Si center in 4 resonates significantly more downfield in the ^29^Si NMR spectrum at −75.8 ppm compared to the starting material (−163.0 ppm); however, it is upfield shifted in comparison to the disilene congener (11.8 ppm).^60^ This can be attributed to the presence of a proton bonded to the central silicon in 4. A resonance of weak intensity at 162.2 ppm in the ^13^C NMR spectrum of 4 was assigned to the CO_2_ moiety inserted in the N–Al bond. Similar characteristic signals in the ^13^C NMR spectrum around 166.0–168.9 ppm have been observed after the addition of CO_2_ to coinage metal derivatives of 1a.^36^ To verify our assignment, we repeated the experiment with ^13^C-labeled CO_2_, yielding the isotopomer 4^13C^ (for more details, see SI). Both carbon signals (188.4 and 162.2 ppm) are strongly intensified in the ^13^C NMR spectra of 4^13C^ compared to 4. Moreover, the resonance corresponding to the central silicon atom appears as a triplet at −75.8 ppm (^1^*J*_C–Si_ = 70.5 Hz) in the ^29^Si NMR spectra of 4^13C^ due to coupling of the Si center with the neighboring ^13^C carbon in the formed Al–Si–C–O ring.^61^

The straightforward capturing of CO_2_ by our system prompted us to investigate the reactivity of 1a towards ketones and aldehydes as well. [2+2]-Cycloaddition reactions of disilenes in this manner are well documented.^27,60,62^ A similar reactivity has recently been described for a phosphagallene, which among others is able to add CO_2_ and benzaldehyde, forming a P–Ga–O–C cycle in the process.^63^ After unsuccessful attempts with benzaldehyde, we chose mesitylaldehyde as a more electron-rich aldehyde derivative to react with 1a. Compound 5 (Scheme 3), characterized by an Al–Si–C–O heterocyclic ring, was isolated as a colorless powder in 74% yield after workup. The SiH group resonates at *δ*_H_ = 4.44 ppm (^1^*J*_Si–H_ = 127 Hz), which is in a similar low-field area to 4 and gives a doublet signal (^3^*J*_H–H_ = 8 Hz) due to proton–proton coupling with the proton of the neighboring CH group. The mesityl-bound CH resonates at *δ*_H_ = 5.65 ppm and matches the ^13^C carbon signal at *δ*_C_ = 65.8 ppm determined *via*^1^H–^13^C HSQC analysis. The central silicon resonates at −59.9 ppm in the ^29^Si NMR spectrum, which is in accordance with the central Si shifts of analogous cycloaddition products obtained from disilene and benzaldehyde (*δ*_Si_ = −54.6/−52.5 ppm).^60^

Single crystals suitable for SC-XRD analysis were obtained by slow evaporation from a concentrated C_6_D_6_ solution at r.t. and confirm the structure of the [2+2]-cycloaddition product 5 (Fig. 1). Compound 5 is characterized by a non-planar Al–Si–C–O heterocycle (*∑* = 353.61°). The distance between the tetrahedral Si and Al center in 5 amounts to 2.5502(5) Å and is significantly elongated with regard to alumanyl silanides in general (2.4179(10) Å, 2.4113(6) Å, 2.3878(10) Å).^35^ The sodium counter ion in 5 is encapsulated by the NHI (contact with the exocyclic nitrogen and one aromatic carbon of the Dipp substituent), one DME molecule, and the oxygen of the formed 4-membered heterocycle. The distance between the sodium cation and the anionic aluminum center amounts to 3.1101(6) Å.

### CO activation

Lastly, we were interested in the ability of alumanyl silanides to activate the stable triple bond in CO.

**Scheme 4:**
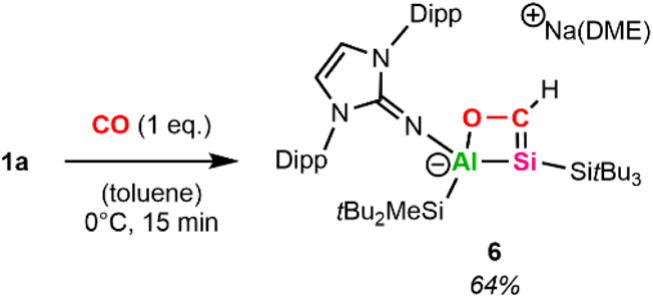
Synthesis of 6.

Pressurizing a toluene-d_8_ solution of 1a with 1 bar CO at 0 °C results in a color change from orange to green. ^1^H NMR spectroscopy reveals the formation of a room-temperature unstable species that decomposes completely within minutes but remains stable for several weeks at −35 °C in toluene. Performing the reaction not with excess, but with 1 equivalent CO at 0 °C in toluene (for more details, see SI) enabled the isolation of the cyclic silacarbene 6 (Scheme 4) as orange powder in 64% yield after workup.

In the ^1^H NMR spectrum of 6, the vinylic proton is observed at 8.73 ppm (^1^*J*_C–H_ = 155 Hz), which is significantly low-field shifted in comparison to previously reported silyl-substituted silacarbenes (5.53–7.43 ppm, ^1^*J*_C–H_ = 133–153 Hz).^64^ This strong deshielding might be caused by the endocyclic oxygen and aluminum in addition to the electropositive silyl-substituents at the four-membered Al–Si–C–O ring. ^13^C and ^29^Si NMR spectra reveal that the Si=C bond resonates at 199.9 ppm (^13^C) and 43.5 ppm (^29^Si). Repeating the experiment employing ^13^C-labeled CO (for more details, see SI) results in the central silicon signal appearing as a doublet (^1^*J*_C–Si_ = 33 Hz) in ^29^Si NMR. To the best of our knowledge, there are no complexes featuring Al–Si–C–O cycles reported in the literature. However, cyclic silacarbenes made from disilenide and carboxylic acid chlorides, which possess a four-membered Si–Si–C–O core formally isovalent to 6, have been isolated.^65^ For said complexes, low-field shifted carbon (213.4–231.6 ppm) and high-field shifted silicon (17.5–34.4 ppm) signals have been observed, pointing to a less polarized Si=C double bond in 6.

**Fig. 2:**
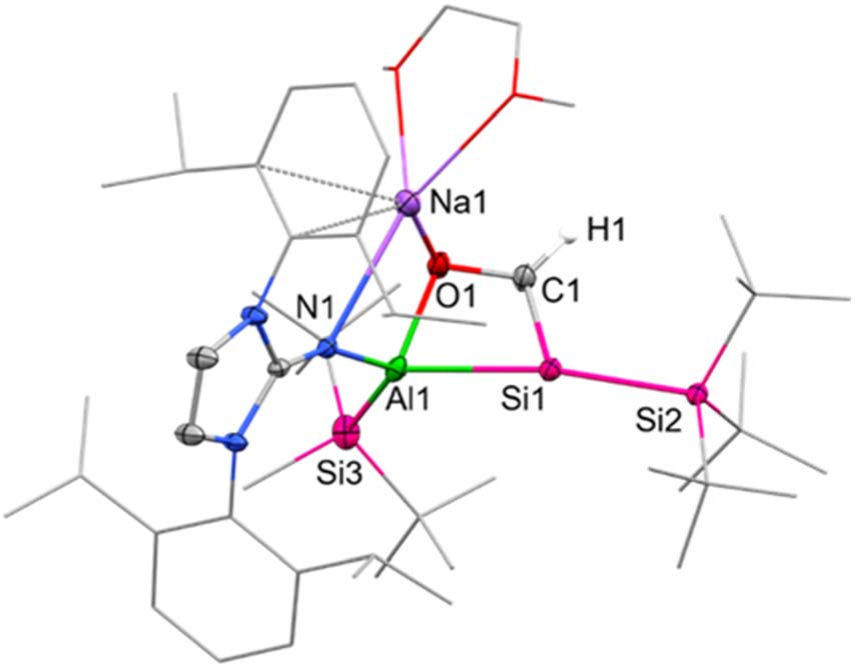
Molecular structure of 6 with thermal ellipsoids drawn at the 20% probability level. Hydrogen atoms are omitted for clarity (except on C1). Selected alkyl and aryl groups are depicted as wireframes. Selected bond lengths [Å] and angles [°]: Al1–Si1 = 2.5596(12), Al1–Si3 = 2.5358(14), Al1–O1 = 1.840(3), O1–C1 = 1.394(4), Si1–C1 = 1.781(4), Si1–Si2 = 2.3841(11), O1–Al1–Si1 = 72.97(8), Al1–Si1–C1 = 68.44(12), Si1–C1–O1 = 114.6(2), C1–O1–Al1 = 101.8(2).

Single crystals suitable for SC-XRD were obtained from cooling a concentrated solution of 6 in hexane at −35 °C. The central Al–Si bond in 6 (Fig. 2) measures 2.5596(12) Å, which is slightly longer than the peripheral Al–Si bond (2.5358(14) Å) in 6 and in range with the central Al–Si bond in 5 (2.5502(5) Å). The endocyclic silicon carbon distance amounts to 1.781(4) Å, which is in accordance with previously reported Si=C bond lengths (1.702(5)–1.778(3) Å).^64^ The C–O distance in 6 amounts to 1.394(4) Å, in line with a carbon–oxygen single bond.^66^ The 4-membered Al–Si–C–O heterocycle is slightly distorted (*∑* = 357.39°), with the 3-coordinated silicon center occupying a pyramidalized geometry (*∑* = 349.14°). Sodium is coordinated by the endocyclic oxygen, the exocyclic nitrogen of the NHI, two aromatic carbon atoms of the Dipp substituent, as well as one molecule of DME, like the coordination found in 5. Based on the structural analysis, 6 is formed *via* [2+2]-cycloaddition of CO across the Al–Si bond, followed by migration of the silicon-bonded proton to the newly introduced carbon of CO.

### Computational study

**Fig. 3:**
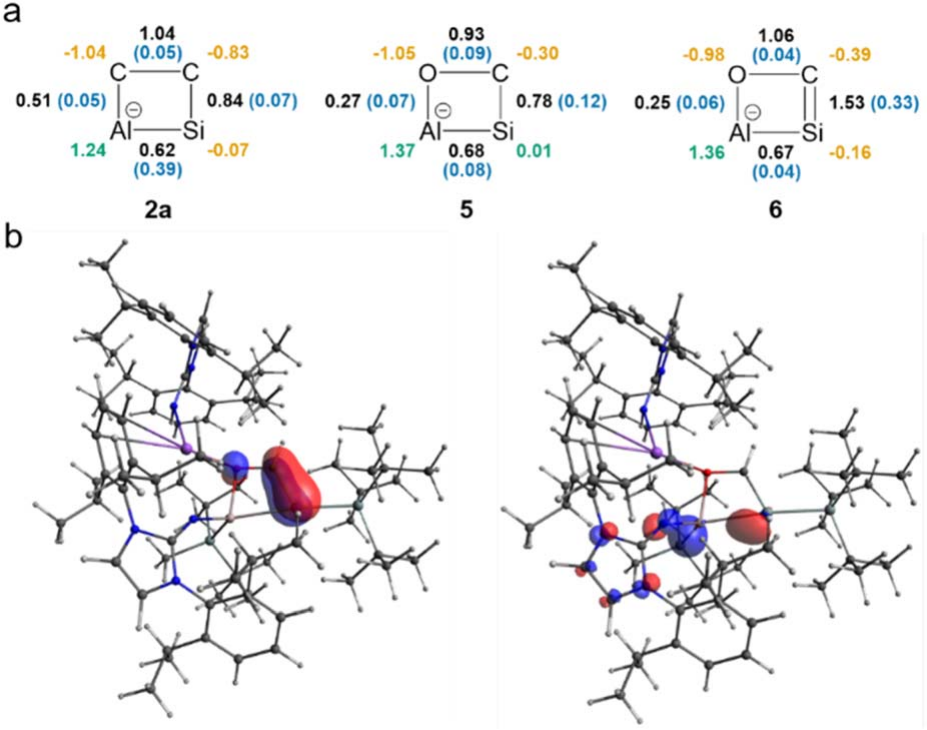
(a) Bonding analysis of 2a, 5, and 6. Wiberg bond indexes (WBI) are shown in black, natural charges are depicted with positive and negative values colored green and orange, respectively. The ellipticity at the BCP is marked in blue. (b) HOMO (left) and HOMO-1 (right) of 6.

To elucidate the bonding situation in the newly formed heterocycles, both QTAIM and NBO analyses were conducted on the optimized structures of 2a, 5, and 6 (for more details, see SI). Upon reaction of 1a with ethylene, mesitylaldehyde, or CO, both the Si and Al centers are oxidized, and the central Al–Si bond order is reduced, resulting in Wiberg bond indices around 0.62–0.68 in 2a, 5, and 6 (Fig. 3). Interestingly, 2a displays a remarkably high ellipticity in the Al–Si Bond Critical Point (BCP), implying a stronger partial π-character found for the Al–Si core in 2a compared to 5 and 6.^67^ Furthermore, the BCPs indicate that the interactions between the Al–Si unit and the substrates are non-covalent in all three cases, indicated by low electron densities and a positive Laplacian, typical features of closed-shell interactions.^68^ The calculations confirm an endocyclic Si=C double bond found in 6 with a WBI of 1.529 and high ellipticity at the BCP (*ε* = 0.3269 a.u.). Following the natural difference in electronegativity between carbon and silicon, NBO analysis shows a σ(Si–C) bond polarized towards carbon (Si/C: 27%/72%; 1.96 electrons occupancy). In contrast, both atoms contribute almost equally (Si/C: 48%/52%) to the purely p-type π-bond with an occupancy of 1.90 electrons, thus indicating an authentic Si=C double bond with significant π-character.

For the polar-covalent endocyclic C–O bond of 6, the WBI value is slightly above 1, suggesting conjugation within the four-membered ring. Moreover, based on NBO analysis, the Al–O interaction in 6 is mainly dative in nature. This is evident from the second-order perturbation analysis, where the donation from the lone pair of O to the empty p-orbital of Al is 42.7 kcal mol^−1^. The QTAIM-derived delocalization index for the Al–O bond is 0.22, which is significantly lower than 1, the expected value for a typical single bond. The highest occupied molecular orbital (HOMO) in 6 corresponds to the Si–C π-bond, which is clearly depicted by the frontier orbitals, whereas the HOMO-1 is delocalized across the Al–Si moiety and to the aluminum center adjacent NHI and silyl group (Fig. 3). The LUMO is delocalized over the aryl substituents at the NHI. This leads to a moderate HOMO–LUMO gap of 3.49 eV. Natural charges (derived from NBO) and QTAIM charges describe an overall polarized heterocycle found in 2a, 5, and 6 (Fig. 3) with a positive and strongly Lewis acidic aluminum center in all cases, despite its fourfold coordination.

**Fig. 4:**
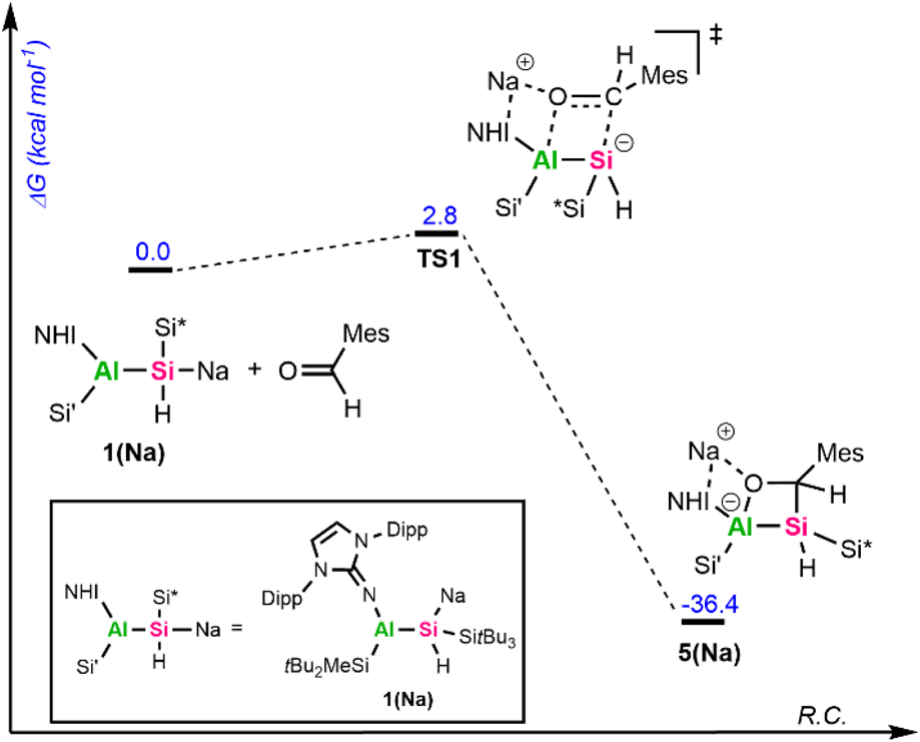
Gibbs free energy diagram of the calculated mechanism involving 1(Na) and mesitylaldehyde (MesCHO, Mes = 2,4,6-trimethylphenyl). Calculated at the SMD(benzene)-PBE0-GD3BJ/Def2-TZVP//PBE0-GD3BJ/Def2-SVP level of theory.

**Fig. 5:**
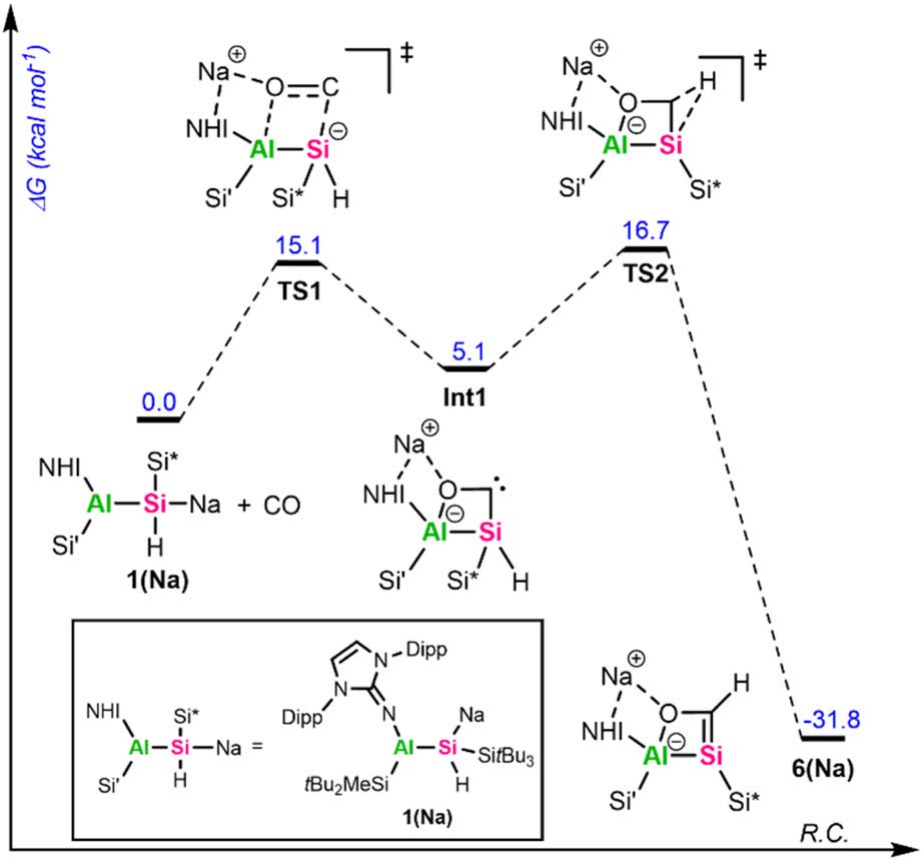
Gibbs free energy diagram of the calculated mechanism involving 1(Na) and CO. The sodium counter ion in TS1, Int1, TS2, and 6(Na) is stabilized through coordination to the Dipp aryl ring and the exocyclic nitrogen of the NHI ligand, as well as by a short contact to the CO oxygen atom. Calculated at the SMD(toluene)-PBE0-GD3BJ/Def2-TZVP//PBE0-GD3BJ/Def2-SVP level of theory.

To get further insights into the mechanism of the formation of 5 and 6, we investigated these reactions computationally using alumanyl silanide 1(Na) with an intimate ion pair with Si–Na contact as model compound (Fig. 4 and 5).

The addition of mesitylaldehyde to 1(Na) does not happen stepwise but proceeds *via* a concerted cyclic transition state (TS) with a small activation energy of 2.8 kcal mol^−1^ (Fig. 4). The addition of CO to 1(Na) begins with an analogous TS1 (Fig. 5), where the Si–C and Al–O bonds are formed simultaneously. Although the reaction barrier here is higher than for the addition of MesCHO, it remains relatively low at 15.1 kcal mol^−1^, leading to the formation of the cyclic singlet carbene Int1. This reactive intermediate features a small HOMO–LUMO gap of 3.04 eV, with the frontier orbitals mainly localized on the lone pair (HOMO) and the empty p-orbital (LUMO) of the carbenic carbon (Fig. S72). Through another low-barrier TS (TS2), 1,2-hydrogen migration from the endocyclic silicon center to the neighboring carbon atom proceeds to yield the silacarbene 6(Na). Both cycloaddition reactions are strongly exergonic (Δ*G* = −36.4 kcal mol^−1^ for 5(Na) and −31.8 kcal mol^−1^ for 6(Na)). The silicon-centered activation of CO was already verified in mechanistic studies for forming sila-ketenyl anions from bis-silyl-substituted silicon anion radicals in our group.^69^ The formation of a transient carbene as an initial step has furthermore been calculated for CO activation by alkali metal amides^9,70^ and phosphides^10^ and was proposed for the reaction of 1,2,4,3-triazaborol-3-yl-lithium with CO.^13^ The group of Apeloig calculated the mechanism for the reduction of CO with Me_3_SiLi.^15^ In their study, a second Me_3_SiLi molecule interacts with the initially formed carbene intermediate, yielding a bis(silyl)-substituted ketyl radical. In contrast, the central silicon in 1a is sterically shielded and possesses a proton in α-position, enabling 1,2-hydrogen migration. For synthesizing cyanides and isocyanides from CO, an analogue of 1,3-silyl migration subsequent to carbene formation has been proposed.^70^ The aforementioned reactions typically proceed *via* initial coordination of the CO oxygen atom to an electropositive metal center, followed by nucleophilic attack at the CO carbon atom to generate a new carbon-based nucleophile that reacts further. Consequently, main-group-mediated CO activation generally affords formal 1,1-carbon-addition products.^3^ In contrast, [2+2]-cycloadditions of CO, as observed with alumanyl silanide 1a, are exceedingly rare in main-group chemistry. For zinc diazo alkyl complexes, a formal [2+3]-cycloaddition of CO has been reported.^71^ However, this activation of CO proceeds *via* initial nucleophilic attack by the diazo nitrogen at the electrophilic CO carbon, facilitated by coordination of the carbon center to zinc and subsequent isomerization. Therefore, this activation is akin to classical silanide-mediated CO insertion. In contrast, the calculations for 1(Na) indicate a truly concerted [2+2]-cycloaddition pathway, which differs from the previously reported reaction pathways and relies on the intrinsic polarity of the central Si–Al bond, with the counter cation playing only a minor role. To the best of our knowledge, a silanide-based hydrosilylation of CO has not been reported to date, despite recent progress in the field of main group-mediated CO activation.^3^

Attempts to trap the proposed cyclic carbene Int1 by addition of transition metal halides ([Rh(COD)Cl]_2_, CuCl), oxidation agents like S_8_ or Woollins' reagent as Se source, or H_2_ at low temperature (−60 °C) to a toluene solution of 1a under CO atmosphere (1 bar) have been unsuccessful. Instead, we were able to grow crystals suitable for SC-XRD by pressurizing a concentrated toluene solution of 1a with 2 bar CO at −35 °C. It is worth mentioning that these crystals are extremely temperature sensitive and decompose above −35 °C by gas evolution, as observed several times under the microscope during the picking process.

**Scheme 5:**
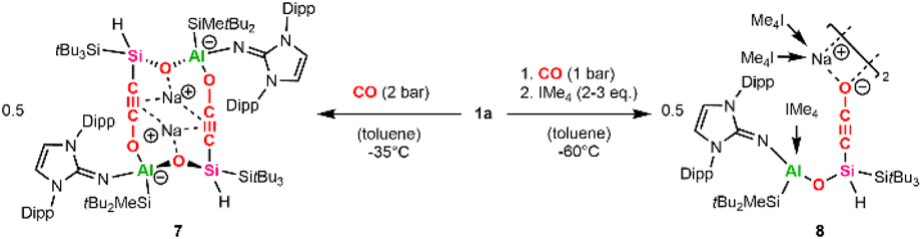
Synthesis of the silanyl ethynolate dimers 7 and 8 (IMe_4_ = 1,3,4,5-tetramethylimidazol-2-ylidene).

Structure analysis revealed the formation of 7 (Scheme 5) from the reaction of 1a with two equivalents of CO, involving C–O bond cleavage and C–C coupling. This results in a silanyl/silyl-substituted ethynolate, with one oxygen inserted into the central Al–Si bond. Two ethynolates then dimerize *via* Al–O bond formation to give the anionic product 7 with two sodium counter ions.

**Fig. 6:**
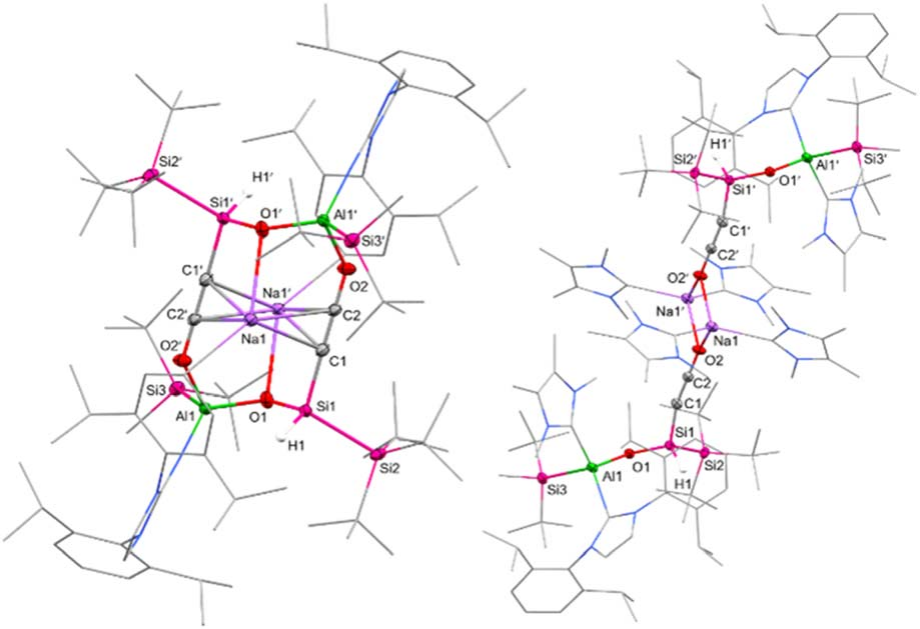
Molecular structures of 7 (left) and 8 (right) with thermal ellipsoids drawn at the 20% probability level. Hydrogen atoms are omitted for clarity (except on Si1/Si1′). Symmetry-generated atoms are marked with a prime. NHC/NHI ligands and selected alkyl groups are depicted as wireframes. Selected bond lengths [Å] and angles [°]: 7: Al1–O1 = 1.744(3), O1–Si1 = 1.627(3), Al1–Si3 = 2.5707(16), Si1–Si2 = 2.4529(13), Si1–C1 = 1.864(3), C1–C2 = 1.219(4), C2–O2 = 1.253(4), Al1–O2 = 1.867(2), Al1–O1–Si1 = 133.00(19). 8: Al1–O1 = 1.7399(13), O1–Si1 = 1.6300(13), Al1–Si3 = 2.5393(7), Si1–Si2 = 2.3694(7), Si1–C1 = 1.7891(19), C1–C2 = 1.219(3), C2–O2 = 1.247(2), Al1–O1–Si1 = 140.46(8).

Compound 7 crystallized in the space group *P*2_1_/*n* and shows a dimeric structure, where the second half is symmetry-generated (Fig. 6, symmetry-generated atoms are marked with a prime). The dimer is characterized by a distorted 12-atom heterocycle, with the two sodium counter ions hovering below and above the non-planar ring. The silicon and the aluminum center are found in a tetrahedral coordination environment with the Al1–O1 bond (1.744(3) Å) being significantly shorter than the Al1–O2′ bond (1.867(2) Å), indicating a weaker interaction between the ethynolate (–C≡C–O^−^) moiety of one half and the corresponding aluminum center of the second half. The Si–C–C–O fragment is almost linear, with measured Si–C–C and C–C–O angles of 178.0(3)° and 179.4(4)°, respectively. Within said fragment, the C1–C2 and C2–O2 bond lengths amount to 1.219(4) Å and 1.253(4) Å, respectively. These values lie between the bond lengths reported for C–C triple bonds and C–O single bonds found in silanyl ethanolates (*d*_C–C_ = 1.18 Å, *d*_C–O_ = 1.31–1.34 Å),^72,73^ as well as C=C and C=O double bonds of bis(silyl)-substituted ketenes (*d*_C–C_ = 1.26–1.28 Å, *d*_C–O_ = 1.18–1.21 Å).^33,34^ Consequently, 7 might be best described by two resonance structures: an ethynolate structure with a C1–C2 triple bond and C2–O2 and O2–Al1 single bonds, and a ketene with a C=C=O fragment stabilizing the aluminum center *via* donor–acceptor interaction between the oxygen and aluminum. An analogous ketene intermediate, formed during the reductive cleavage of CO using disilenide, has been confirmed *via* trapping using transition metal hexacarbonyls (M(CO)_6_, M = Cr, Mo, W).^33^ Despite numerous attempts, we were not able to isolate a room-temperature stable derivative of 7.

The formation of 7 presents a rare example of main-group mediated reductive homologation of CO.^3,32^ This is enabled by the cooperative interaction of the Lewis acidic aluminum center and the neighboring nucleophilic silicon center, stabilized by a sodium counter cation. Similar reactions have been reported for main group complexes involving B–Li,^13,14^ Mg–Mg,^20–26^ Si=Si^33^ and B≡B^29^ bonds as active centers. Additionally, CO homologations mediated by acyclic silylenes,^74^ bis(silylenes),^34,75^ aluminum(i) compounds^76^ and dialanes^19^ are known.

The CO activation through alumanyl silanide 1a is proposed to proceed in an analogous fashion to that reported for lithium disilenide.^33^ For both systems, one key step is the formation of a heterocyclic carbene. The carbene is stabilized by a second CO molecule acting as a Lewis base, yielding a ketene, a step that is corroborated by preliminary DFT calculations (see SI). In the case of the disilenide, calculations on a simplified model showed a rearrangement after ketene formation, resulting in a silanyl/silyl-substituted ethynolate. In our case, we assume the heterocyclic Al–Si–C–O ring to rearrange after ketene formation in a similar manner with cleavage of the highly polarized central Al–Si bond and the endocyclic C–O bond and formation of a thermodynamically favored Si–O bond. The resulting silanyl ethynolate then dimerizes through reaction of the terminal oxygen with the aluminum center of a second silanyl ethynolate to give 7.

We assumed the addition of NHC as a stronger Lewis base would better stabilize the cyclic carbene intermediate than an additional molecule of CO. Since 1a reacts with 1 equivalent of NHC *via* exchange of the DME donor, we added 2–3 equivalents of IMe_4_ (1,3,4,5-tetramethylimidazol-2-ylidene) to a cooled (−60 °C) toluene solution of 1a under CO (1 bar) atmosphere. This approach yielded the NHC-stabilized silanyl ethynolate 8 (Scheme 5). Like 7, compound 8 is not stable at ambient temperature and could not be isolated. However, the solid-state structure of 8 (Fig. 6) was unambiguously determined using SC-XRD after obtaining colorless cuboid-shaped crystals from a concentrated toluene solution at −35 °C under CO atmosphere. Instead of the anticipated NHC-stabilized carbene, the structure of 8 presents a silanyl ethynolate dimer analogous to 7, with the second half of the molecule being symmetry-generated. The two fragments in 8 are bridged by the two sodium counter ions, which each coordinate to the terminal oxygen of the ethynolate moiety and the carbenic carbon of two IMe_4_ molecules, respectively. Thus, the addition of IMe_4_ inhibits the dimerization *via* Al–O bond formation as observed for 7. Another equivalent of IMe_4_ coordinates to the aluminum center, resulting in a tetrahedral geometry. The Al–C distance (2.090(2) Å) lies in the upper range of reported Al–C_IMe4_ bond lengths (1.99–2.09 Å).^77^ Notably, the distance between the exocyclic nitrogen of the NHI and the Al center measures 1.819(7) Å and is significantly elongated in comparison to the Al1–N1 bond length found in 7 (1.751(2) Å) or reported alumanyl silanides (1.77–1.78 Å).^35^ This marks a strong σ-interaction between the NHC and the aluminum center and a weakened bonding towards the NHI. The C1–C2 and C2–O2 bond distances in 8 measure 1.219(3) Å and 1.247(2) Å and match the respective bond lengths in 7. Dissentingly, the Si–C–C–O fragment in 8 is less linear with Si–C–C and C–C–O bond angles of 167.02(17)° and 178.9(2)°, respectively, owing to steric repulsion exerted by the coordinating NHC ligands. Another difference is a considerably shorter Si1–C1 bond found in 8 with respect to 7 (7: *d*_Si1–C1_ = 1.864(3) Å, 8: *d*_Si1–C1_ = 1.7891(19) Å) or literature known silyl ethynolates (1.83–1.84 Å).^72,73^ Most recently, a silyl ethynolate dimer bridged by two Eu(ii) centers was reported, exhibiting almost linear geometry (Si–C–C/C–C–O angles = 176.4(5)°/179.4(7)°) and comparable bond lengths (*d*_C–O_ = 1.25 Å, *d*_C–C_ = 1.20 Å, *d*_Si–C_ = 1.79 Å) to those in 8.^78^ NRT analysis for this silyl ethynolate indicates a major ethynolate resonance contribution (74.1%) alongside a substantial ketenyl component (22.9%) despite its linear geometry and four-coordinate silicon center. Consistent with these findings, a predominant ethynolate character for 7 and 8, with minor contributions from a possible ketene/ketenyl resonance structure, is assumed.

## Conclusions

In summary, we report the isolation and characterization of previously unknown Al–Si–C–C (2a, 2b, 3a, 3b) and Al–Si–C–O heterocycles (4, 5). These were obtained by the formal [2+2]-cycloaddition of ethylene, styrene, mesitylaldehyde, and CO_2_ to the polarized Al–Si bond found in alumanyl silanide/aluminata-silene 1a/1b. In the presence of 1 equivalent CO, an Al–Si–C–O heterocycle containing a Si–C double bond is formed *via* CO addition to the Al–Si core of 1a, followed by proton migration. The formal hydrosilylation of the stable C≡O bond in carbon monoxide once more attests the potential of heavier heteroatomic low-valent main group compounds for oxidative addition and further functionalization of small molecules, both critical steps in catalytic cycles. The formation of 6 is proposed to proceed *via* a heterocyclic carbene intermediate, which could be trapped in the presence of additional CO and NHC at low temperature, yielding the silanyl ethynolates 7 and 8. Thus, the reductive coupling of CO was observed for the first time for a low-valent compound with an Al–Si bond as the active site. All things considered, this in-depth reactivity study of alumanyl silanides has experimentally confirmed the multiple bond character found for the anionic Al–Si core of the title compound. Reactions that have been usually observed for heavier homodiatomic main-group compounds like disilenes have now been attested with heterodiatomic congeners, which formally possess the [Al–Si^−^] core isovalent to [Si=Si]. We hope that these results will fuel the search for heavier heterodiatomic low-valent main group species, for which examples are exceedingly rare, and many combinations across the periodic table are still missing.

## Author contributions

M. L. and J. V. conceived and performed the synthetic experiments and analysed the data. P. V. designed and performed the theoretical analyses. S. S. measured, solved, and refined the XRD data. S. I. conceived and supervised the project. M. L., J. V. and S. I. wrote the manuscript with input and critical revision from all authors.

## Conflicts of interest

There are no conflicts to declare.

## Supplementary Material

SC-017-D5SC09910B-s001

SC-017-D5SC09910B-s002

SC-017-D5SC09910B-s003

## Data Availability

CCDC 2499949 (2a), 2499950 (5), 2499951 (6), 2499952 (7), 2499953 (8) and 2499954 (6′) contain the supplementary crystallographic data for this paper.^79*a*–*f*^ All data supporting this article have been included in the main text or the supplementary information (SI). Supplementary information: full experimental procedures, NMR spectra, SC-XRD structures, FTIR spectra, computational details, and optimized xyz-coordinates. See DOI: https://doi.org/10.1039/d5sc09910b.
