# Supplementary material for: Alumanyl silanides as multifunctional reagents for olefin cycloaddition, CO hydrosilylation, and reductive CO coupling
Source: Chem Sci. 2026 Jan 15;17(11):5463–73. doi: 10.1039/d5sc09910b (PMC12824870; doi:10.1039/d5sc09910b)
Supplement: SC-017-D5SC09910B-s002 [file SC-017-D5SC09910B-s002.pdf]

This journal is © The Royal Society of Chemistry 2026

Table of Contents

1.) Experimental Details 2

2.) Crystallographic Details 75

3.) Details of Theoretical Calculations 84

4.) Supplementary References 91

1.) Experimental Details

General considerations: All manipulations were carried out under exclusion of H_2O and O_2 under an atmosphere of argon 4.6 (≥99.996%; Westfalen AG) using standard Schlenk techniques or in a LABstar glovebox from MBraun Inertgas-Systeme GmbH with H_2O and O_2 levels below 0.5 ppm. The glassware used was heat-dried under fine vacuum prior to use with Triboflon III grease (mixture of polytetrafluoroethylene (PTFE) and perfluoropolyether) from Freudenberg & Co. KG as sealant for glass junctions. For stirring, PTFE-coated magnetic stirrer bars were used or glass-coated ones if stated. Liquid phases were transferred using standard PE/PP syringes equipped with stainless steel cannula or directly canted from vessel to vessel if not stated otherwise. Solvents were dried by standard methods (e.g. withdrawal from MBraun Solvent Purification System, storage over molecular sieves (3 Å), degassing via freeze-pump-thaw cycling, distillation from sodium/ketylradical or CaH_2). Unless otherwise stated, all commercially available chemicals were purchased from abcr GmbH or Sigma-Aldrich and used without further purification. Carbon monoxide (CO) 4.7 (≥99.997%) was purchased from Westfalen AG and used as received. CO_2 and ethylene was purchased from Westfalen AG and used as received. The reagents alumanyl silanide **1a** ,aluminata-silene **1b** and IMe_4 were prepared according to the reported procedures.[1, 2] The nuclear magnetic resonance spectra (NMR) were recorded on a *Bruker DRX400* (^{1}H: 400.13 MHz, ^{13}C: 100.62 MHz, ^{29}Si:79.49 MHz), *AV500* (^{1}H: 500.13 MHz) or *AV500C* (^{1}H: 500.36 MHz, ^{13}C: 125.83 MHz, ^{29}Si: 99.41 MHz) spectrometer at ambient temperature (300 K) and referenced to residual solvent signals as internal standards (^{1}H and ^{13}C) or an external standard ($SiMe_4$ for ^{29}Si). Values for the chemical shift (*δ*) are given in parts per million. In some NMR spectra, signals from silicone grease (C_6D_6: δ(^{1}H) = 0.29 ppm, δ(^{13}C) = 1.4 ppm and δ(^{29}Si) = -21.8 ppm), originating from the cannulas used (*B. Braun Melsungen AG Sterican*®), can be observed. Assignment of ^{13}C resonances was mostly supported by ^{1}H^{13}C HSQC or ^{1}H^{13}C HMQC experiments. Quantitative elemental analyses (EA) were measured with a *EURO EA (HEKAtech)* instrument equipped with a CHNS combustion analyzer at the *Laboratory for Microanalysis* at the *TUM Catalysis Research Center*. Infrared (IR) spectra were recorded on a Perkin Elmer FT-IR spectrometer (diamond ATR, Spectrum Two) in a range of 400–4000 cm^{-1} at room temperature inside an argon-filled glovebox. Melting Points (m.p.) were determined in sealed glass capillaries under inert gas by a Büchi M-565 melting point apparatus.

Abbreviations: s = singlet, d = doublet, t = triplet, sept = septet, IMes = 1,3-bis(mesityl)-imidazolin-2-ylidene, mesityl = 2,4,6-trimethylphenyl, TMS = trimethylsilyl ($SiMe_3$), SC-XRD = Single Crystal X-ray diffraction, IG = Inverse-Gated, INEPT = Insensitive Nuclei Enhanced by Polarization Transfer, HSQC = Heteronuclear Single Quantum Coherence/Correlation, HMBC = Heteronuclear Multiple Bond Correlation.

Synthesis of Ethylene Cycloaddition Product (2a)

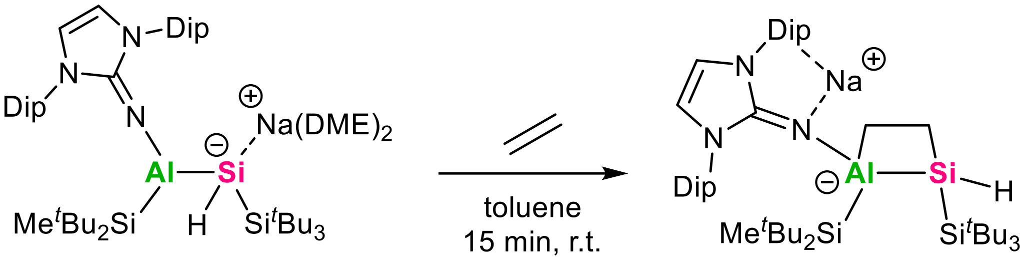

1a **2a**

A solution of alumanyl silanide **1a** (80 mg, 78.5 µmol, 1 eq) in toluene (5 mL) was degassed via three freeze-pump-thaw cycles. After pressurizing the reaction mixture with ethylene (1.3 bar), a color change from orange to yellow was observed within 15 minutes at r.t.. The solvent was removed in a dynamic vacuum. Recrystallization from a concentrated pentane solution (1 mL) over 1 week at -35°C yielded **2a** as light yellow crystals (59.0 mg, **72%**), suitable for SC-XRD.

^{1}H NMR (400.1 MHz, C_6D_6): δ = 7.08 (m, 6H, DipH), 5.96 (s, 2H, NC*H*), 3.37 (sept, ^3J = 7 Hz, 2H, C*H*-^{i}Pr), 3.17 (sept, ^3J = 7 Hz, 2H, C*H*-^{i}Pr), 2.72 (bs, 1H, CH_2-ethylene), 1.78 (bs, 1H, CH_2-ethylene), 1.45 (d, ^3J = 7 Hz, 6H, CH_3-^{i}Pr), 1.39 (d, ^3J = 7 Hz, 6H, CH_3-^{i}Pr), 1.27 (s, 28H, Si^{t}Bu$_3$ + Si*H*), 1.21 (s, 9H, Si^{t}Bu$_2$), 1.13 (s, 9H, Si^{t}Bu$_2$), 1.09 (d, ^3J = 7 Hz, 12H, CH_3-^{i}Pr), 0.97 (bs, 1H, CH_2-ethylene), 0.43 (bs, 1H, CH_2-ethylene), 0.06 (s, 3H, SiCH_3). **^{13}C{^{1}H} NMR** (100.6 MHz, C_6D_6): δ = 149.2 (NHI-C_{aryl}), 149.1 (NHI-C_{aryl}), 144.0 (NHI-C_{aryl}), 135.9 (NHI-C_{aryl}), 129.7 (NHI-C_{aryl}), 125.1 (NHI-C_{aryl}), 124.3 (NHI-C_{aryl}), 114.4 (N*C*H), 32.1 (Si^{t}Bu$_3$), 31.1 (Si^{t}Bu$_2$), 30.9 (Si^{t}Bu$_2$), 29.0 (*C*H-^{i}Pr), 28.7 (*C*H-^{i}Pr), 25.4 (*C*H$_3$-^{i}Pr), 25.1 (*C*H$_3$-^{i}Pr), 24.2 (Si^{t}Bu$_3$), 23.6 (Si^{t}Bu$_2$), 22.8 (Si^{t}Bu$_2$), 22.7 (Si^{t}Bu$_2$), 20.7 (Si^{t}Bu$_2$), 18.4 (*C*H$_2$-ethylene), 7.3 (*C*H$_2$-ethylene), -4.9 (Si*C*H$_3$). **^{29}Si{^{1}H} NMR** (99.4 MHz, C_6D_6): δ = 17.0 (*Si*^{t}Bu$_3$), -0.2 (*Si*^{t}Bu$_2$), -82.3 (*Si*H).

FT-IR (neat, cm^{-1}): $\tilde{\nu}$ = 1973 (s) (Si-H).

Melting point: 134.5°C

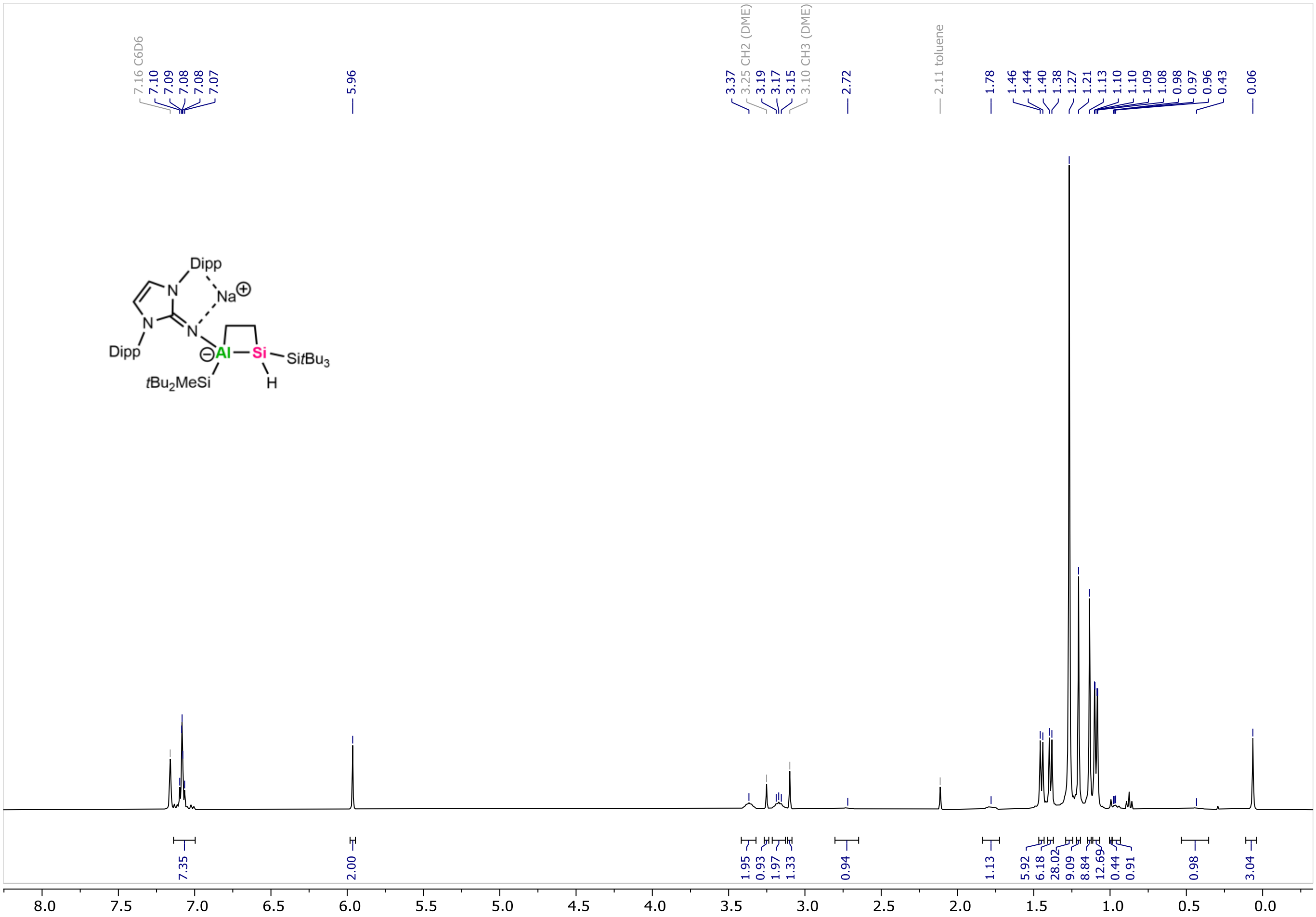

Figure S1: ^{1}H NMR spectrum (400.1 MHz, C_6D_6) of **2a**. Residual solvent and DME signals are marked in grey.

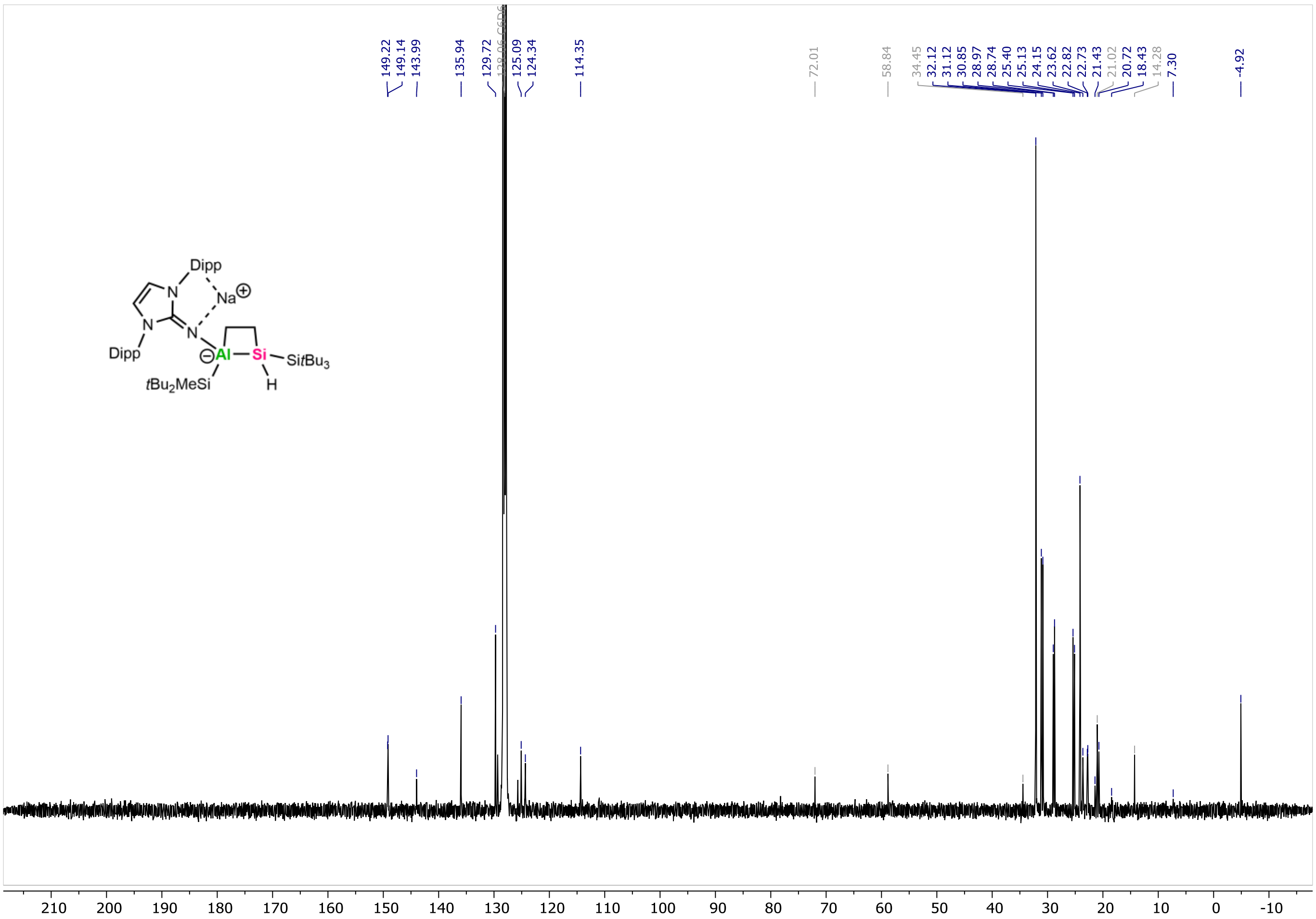

Figure S2: $^{13}C\{^{1}H\}$ NMR spectrum (100.6 MHz, C_6D_6) of **2a**. Residual solvent and DME signals are marked in grey.

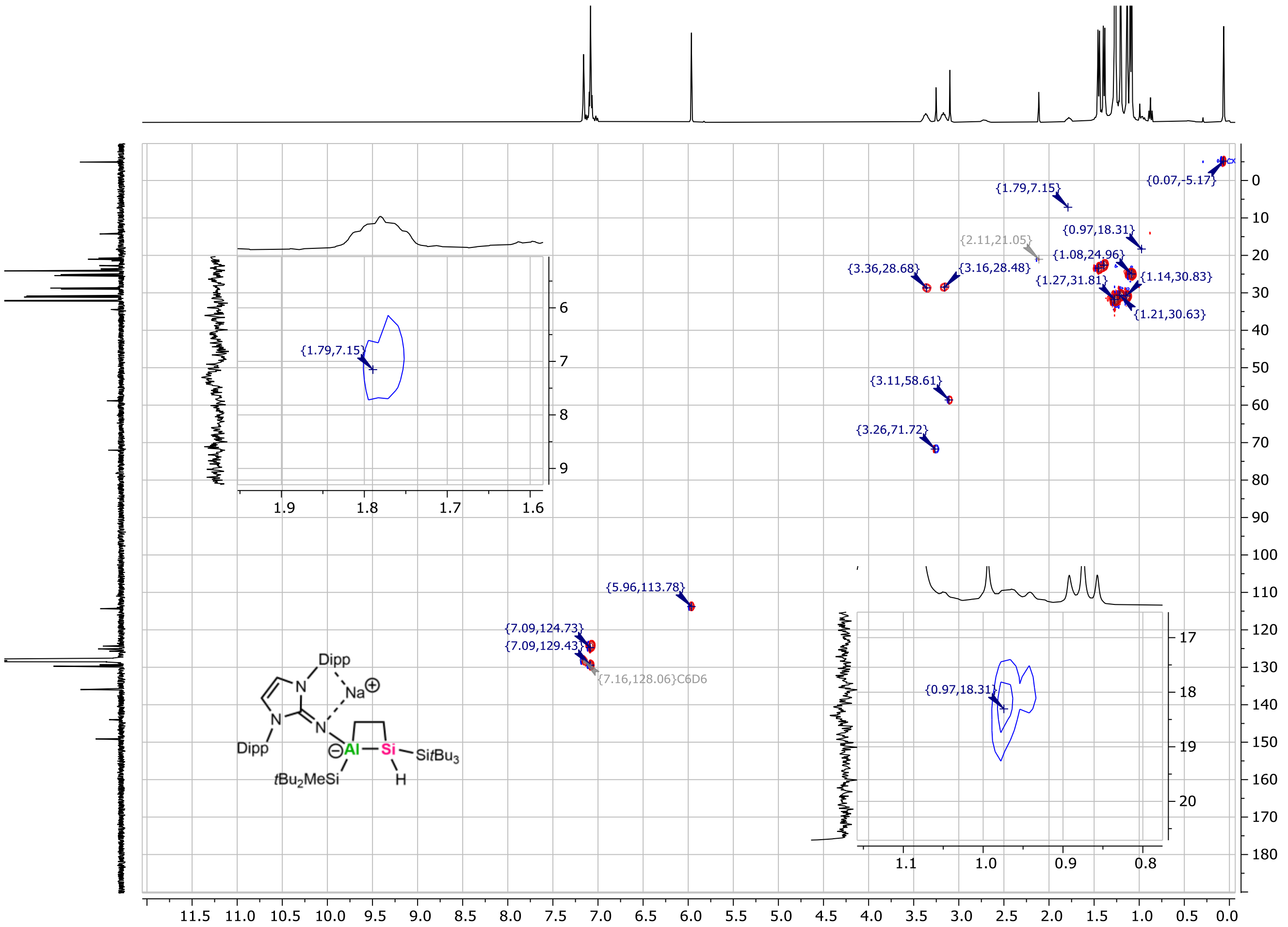

Figure S3: $^{1}H^{13}C$ HSQC NMR spectrum of **2a** with $^{13}C\{^{1}H\}$ spectrum as vertical trace and ^{1}H spectrum as horizontal trace including "zoom in" on the CH_2-ethylene signals.

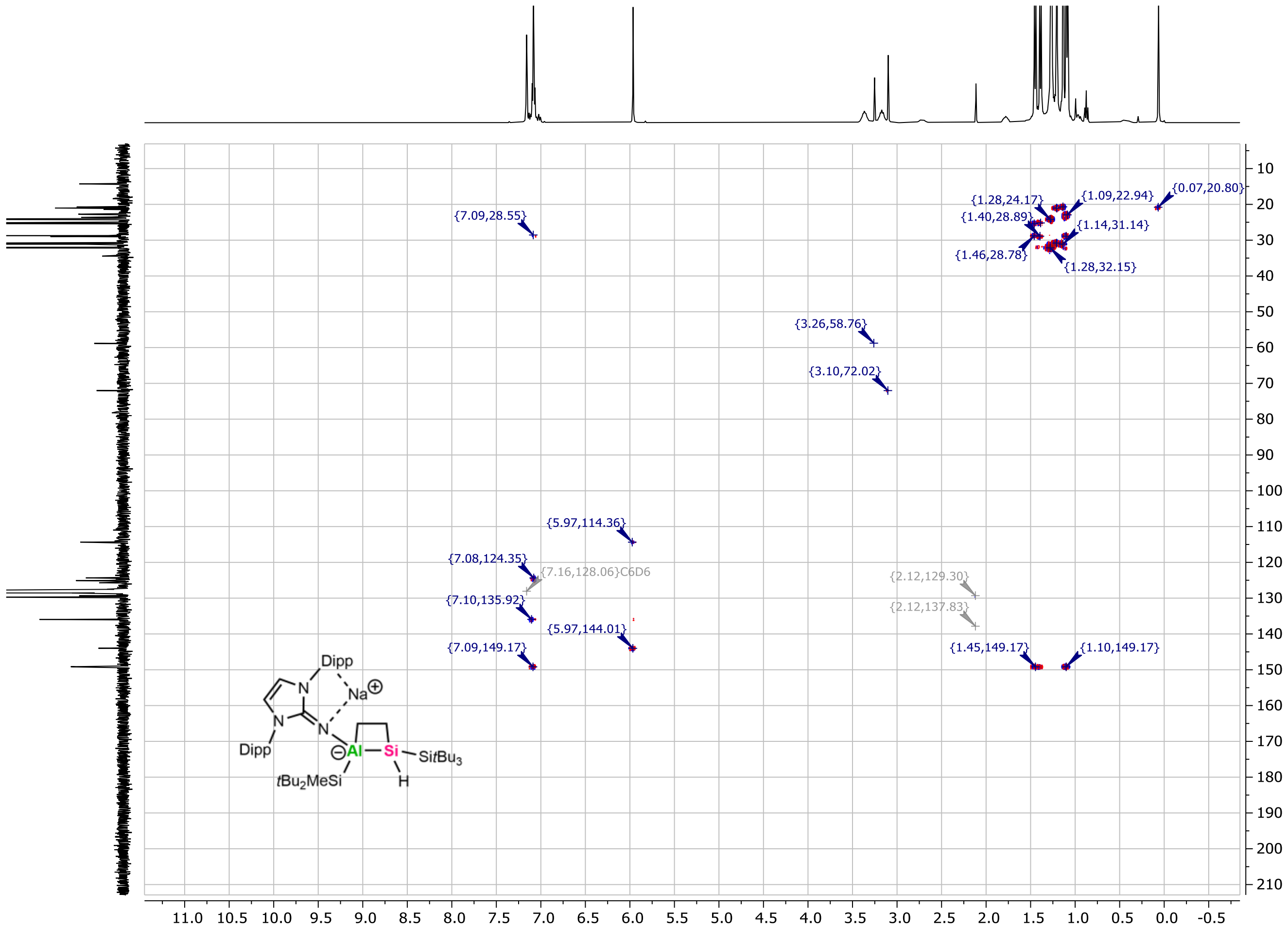

Figure S4: $^{1}H^{13}C$ HMBC NMR spectrum of **2a** with $^{13}C\{^{1}H\}$ spectrum as vertical trace and ^{1}H spectrum as horizontal trace.

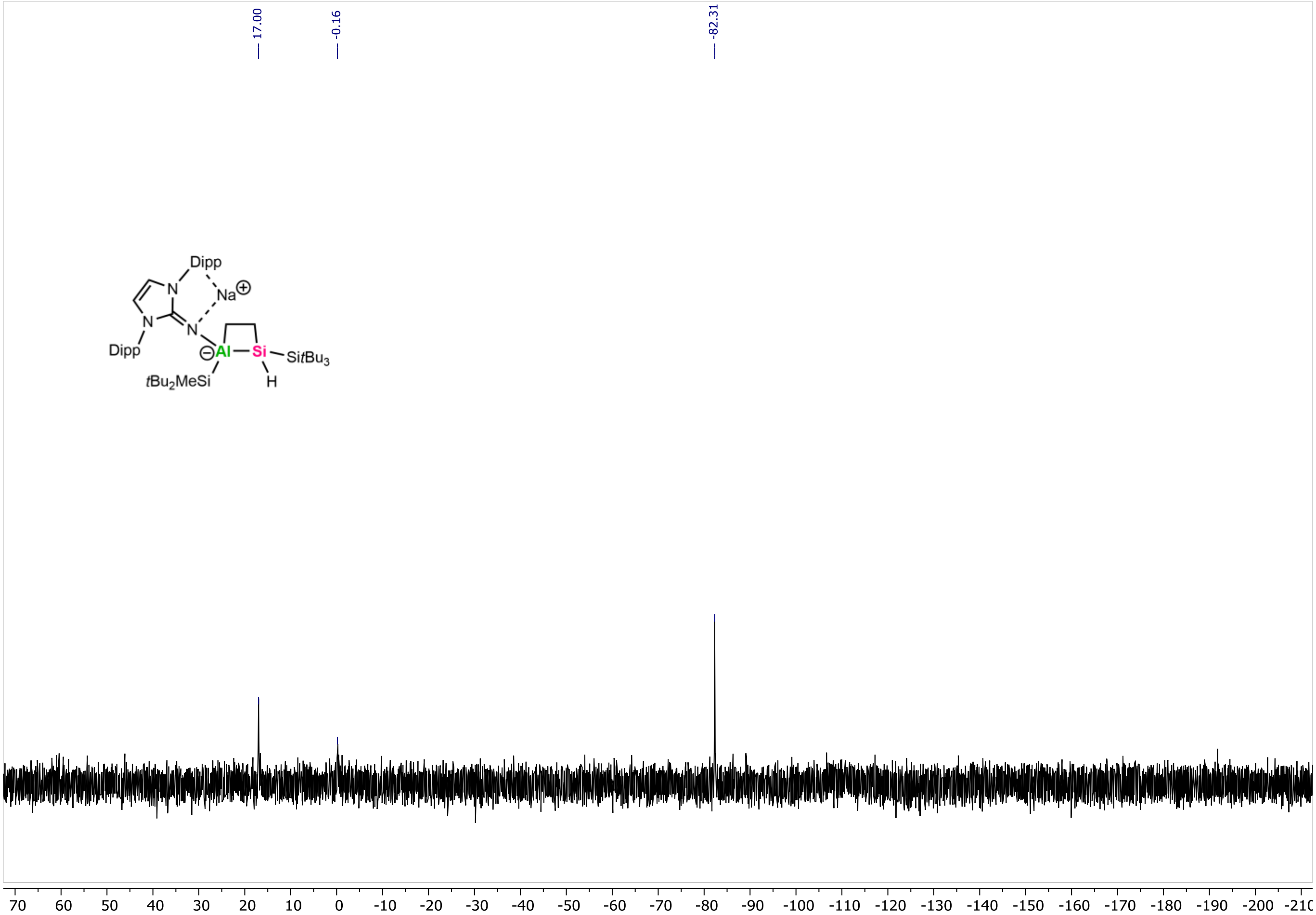

Figure S5: $^{29}Si\{^{1}H\}$ NMR spectrum (99.4 MHz, C_6D_6) of **2a**.

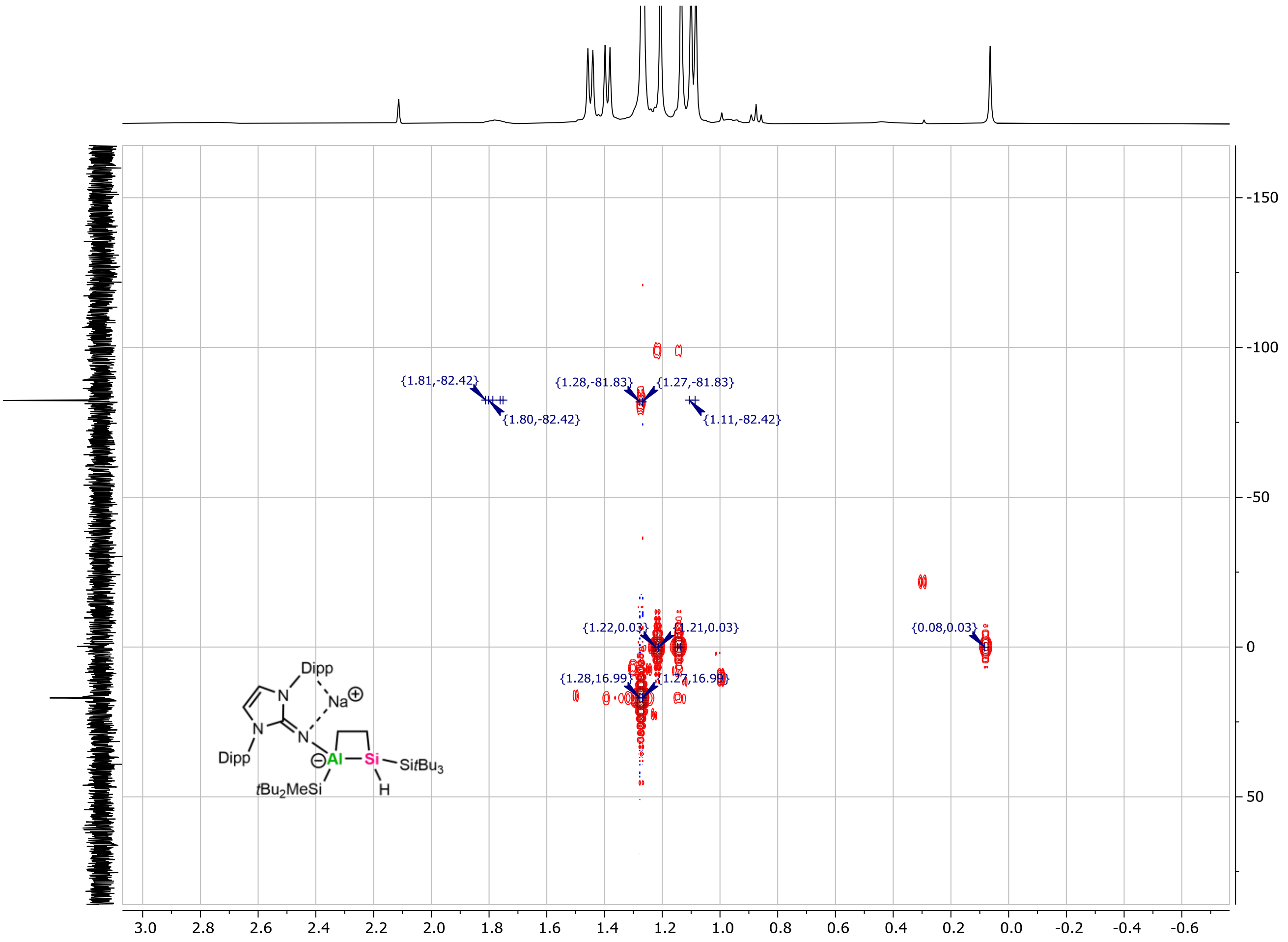

Figure S6: $^{1}H^{29}Si$ HMBC NMR spectrum of **2a** with $^{29}Si\{^{1}H\}$ spectrum as vertical trace and ^{1}H spectrum as horizontal trace. The spectrum is referenced to the ***H***Si***Si***^{t}Bu$_3$ cross-peak according to the ^{1}H and $^{29}Si\{^{1}H\}$ spectral data.

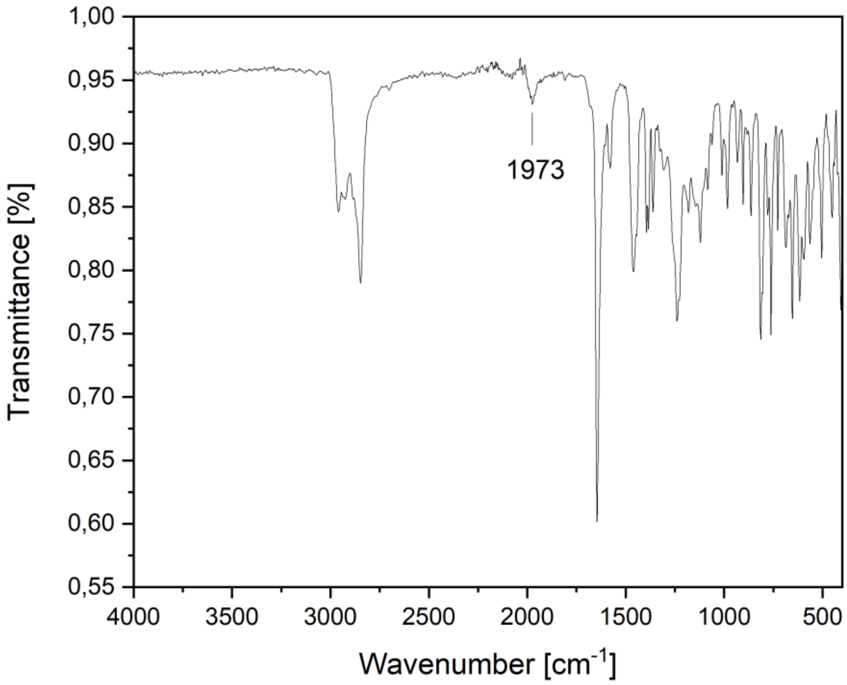

Figure S7: Solid-state FT-IR spectrum of **2a**. The position of the Si-H band is marked.

Synthesis of Cryptand Stabilized Ethylene Cycloaddition Product (2b)

Method A:

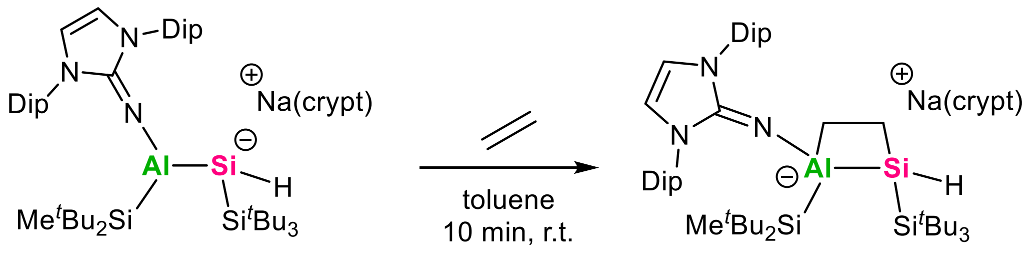

1b **2b**

A solution of aluminata silene **1b** (90 mg, 84.1 µmol, 1 eq) in toluene (4 mL) was degassed via three freeze-pump-thaw cycles. After pressurizing the reaction mixture with ethylene (1.5 bar), a color change from orange to colorless was observed within 10 min at r.t.. The volatiles were removed under reduced pressure, affording an off-white residue, which was washed with diethyl ether (1 mL) and dried to obtain **2b** as a white powder (59.6 mg, **65%**).

Method B:

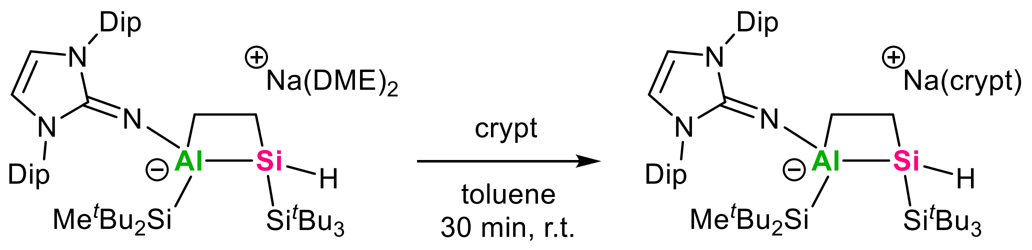

2a **2b**

Cycloaddition product **2a** (16.5 mg, 14.7 µmol, 1 eq) was dissolved in deuterated benzene (0.4 mL). After addition of [2.2.2]cryptand (5.53 mg, 14.7 µmol, 1 eq), the colorless solution was left at r.t. for 30 min. The product was obtained quantitatively, elucidated by NMR analysis.

^{1}H NMR (400.1 MHz, C_6D_6): δ = 7.37 (m, 6H, DipH), 6.23 (s, 2H, NC*H*), 3.91 (sept, ^3J = 7 Hz, 4H, C*H*-^{i}Pr), 3.10 (s, 1H, Si*H*), 3.07 (s, 12H, OC*H*$_2$-crypt), 3.07 (t, ^3J = 5 Hz, 12H, OC*H*$_2$-crypt), 2.53 (m, 1H, C*H*$_2$-ethylene), 2.04 (t, ^3J = 5 Hz, 12H, NC*H*$_2$-crypt), 1.77 (dd, ^3J = 3 Hz, ^3J = 7 Hz, 12H, C*H*$_3$-^{i}Pr), 1.59 (s, 27H, Si^{t}Bu$_3$), 1.45 (dd, ^3J = 2 Hz, ^3J = 7 Hz, 12H, C*H*$_3$-^{i}Pr), 1.41 (bs, 1H, C*H*$_2$-ethylene), 1.38 (s, 9H, Si^{t}Bu$_2$), 1.34 (s, 9H, Si^{t}Bu$_2$), 0.89 (m, 1H, C*H*$_2$-ethylene), 0.77 (m, 1H, C*H*$_2$-ethylene), 0.30 (s, 3H, SiCH$_3$), 0.24 (m, 1H, C*H*$_2$-ethylene). **^{13}C{^{1}H} NMR** (100.6 MHz, C_6D_6): δ = 148.8 (NHI-*C*$_{aryl}$), 148.6 (NHI-*C*$_{aryl}$), 138.7 (NHI-*C*$_{aryl}$), 136.6 (NHI-*C*$_{aryl}$), 123.8 (NHI-*C*$_{aryl}$), 113.8 (N*C*H), 68.6 (O*C*H$_2$-crypt), 67.8 (O*C*H$_2$-crypt), 53.0 (N*C*H$_2$-crypt), 33.2 (Si^{t}Bu$_3$), 32.0 (Si^{t}Bu$_2$), 28.9 (*C*H-^{i}Pr), 25.4 (*C*H$_3$-^{i}Pr), 24.7 (Si^{t}Bu$_3$), 24.5 (*C*H$_3$-^{i}Pr), 24.2 (*C*H$_3$-^{i}Pr), 22.0 (Si^{t}Bu$_2$), 21.5 (Si^{t}Bu$_2$), 20.6 (*C*H$_2$-ethylene), 10.6 (*C*H$_2$-ethylene), -3.5 (Si*C*H$_3$). **^{29}Si{^{1}H} NMR** (99.4 MHz, C_6D_6): δ = 16.8 (*Si*^{t}Bu$_3$), -1.0 (*Si*^{t}Bu$_2$), -80.2 (*Si*H).

FT-IR (neat, cm^{-1}): $\tilde{\nu}$ = 1996 (s) (Si-H).

Melting point: 185.0°C

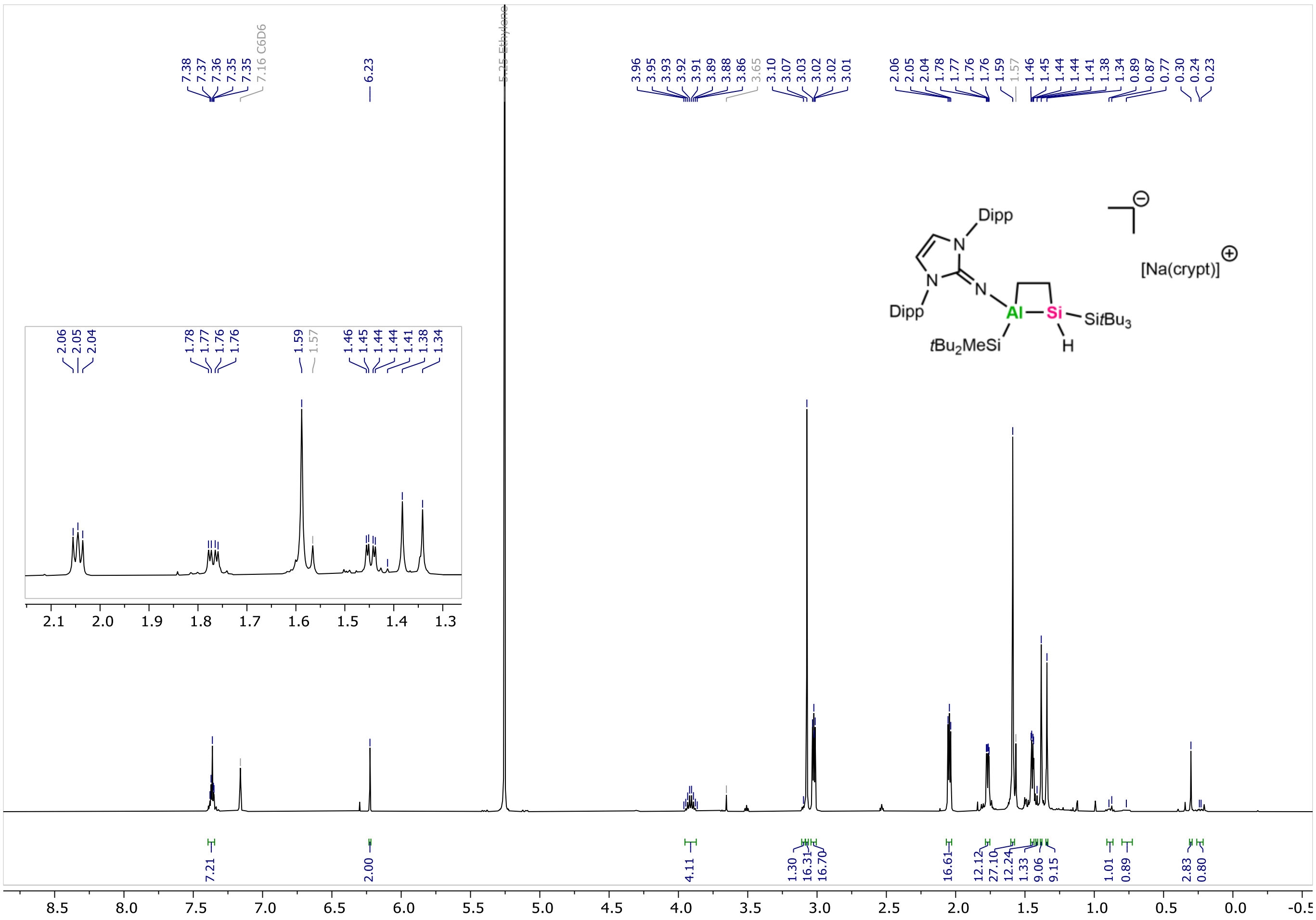

Figure S8: ^{1}H NMR spectrum (400.1 MHz, C_6D_6) of **2b** with “zoom in” on the aliphatic region. Residual solvent and DME signals are marked in grey.

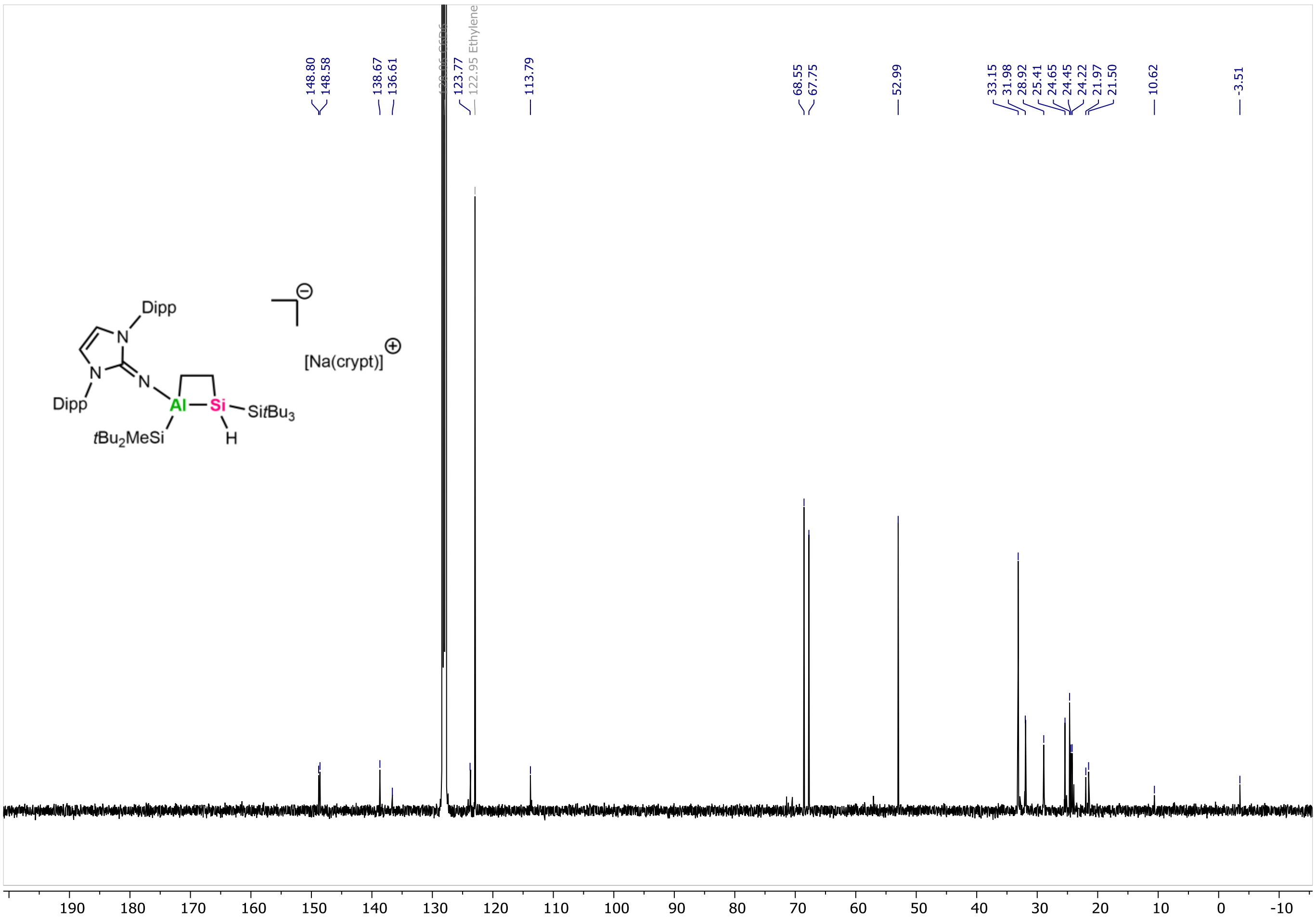

Figure S9: $^{13}C\ \{^1H\}$ NMR spectrum (100.6 MHz, C_6D_6) of **2b**. The signal of free ethylene is marked in grey.

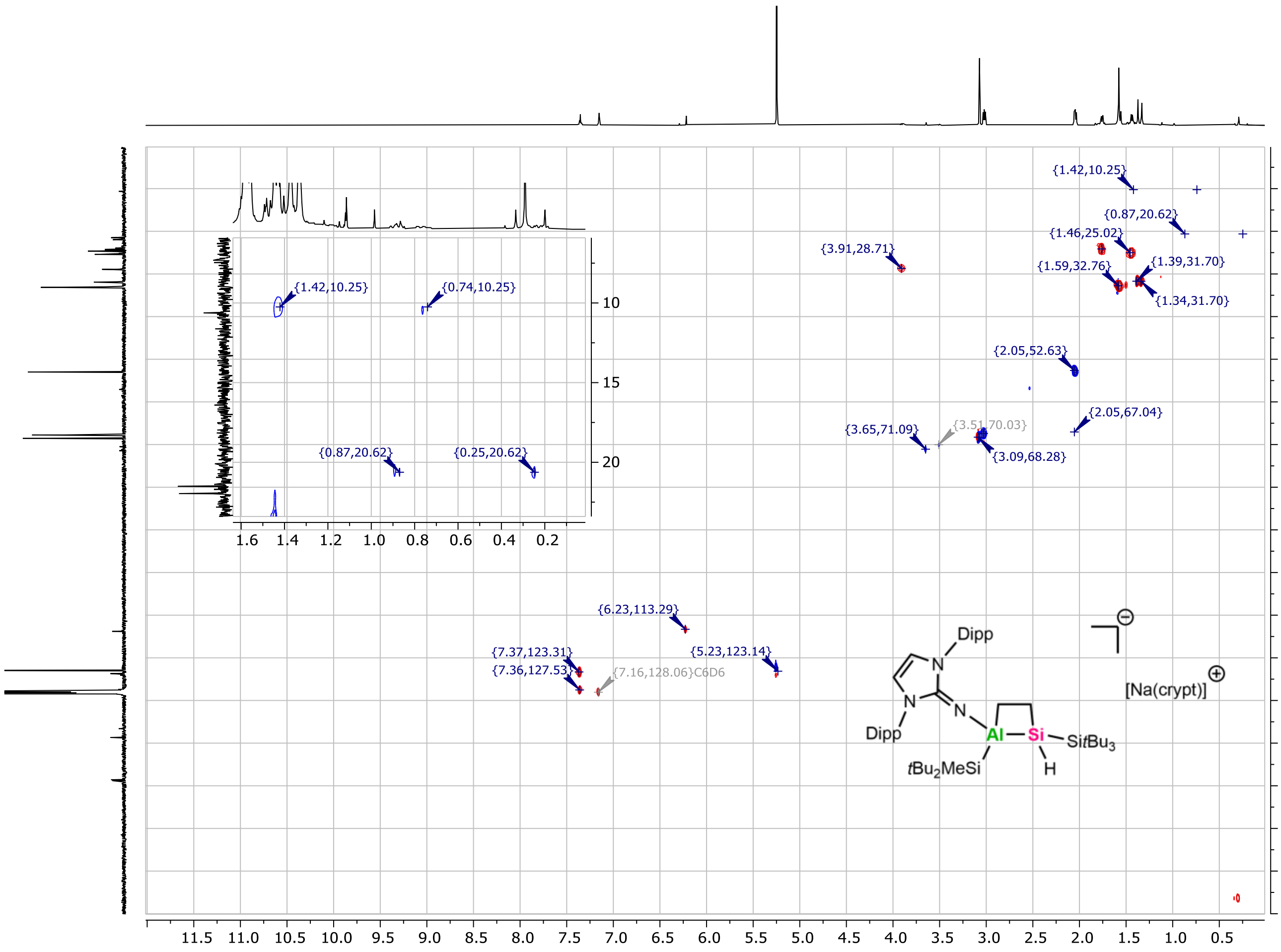

Figure S10: $^{1}H^{13}C$ HSQC NMR spectrum of **2b** with $^{13}C\{^{1}H\}$ spectrum as vertical trace and ^{1}H spectrum as horizontal trace including “zoom in” on the CH_2-ethylene signals.

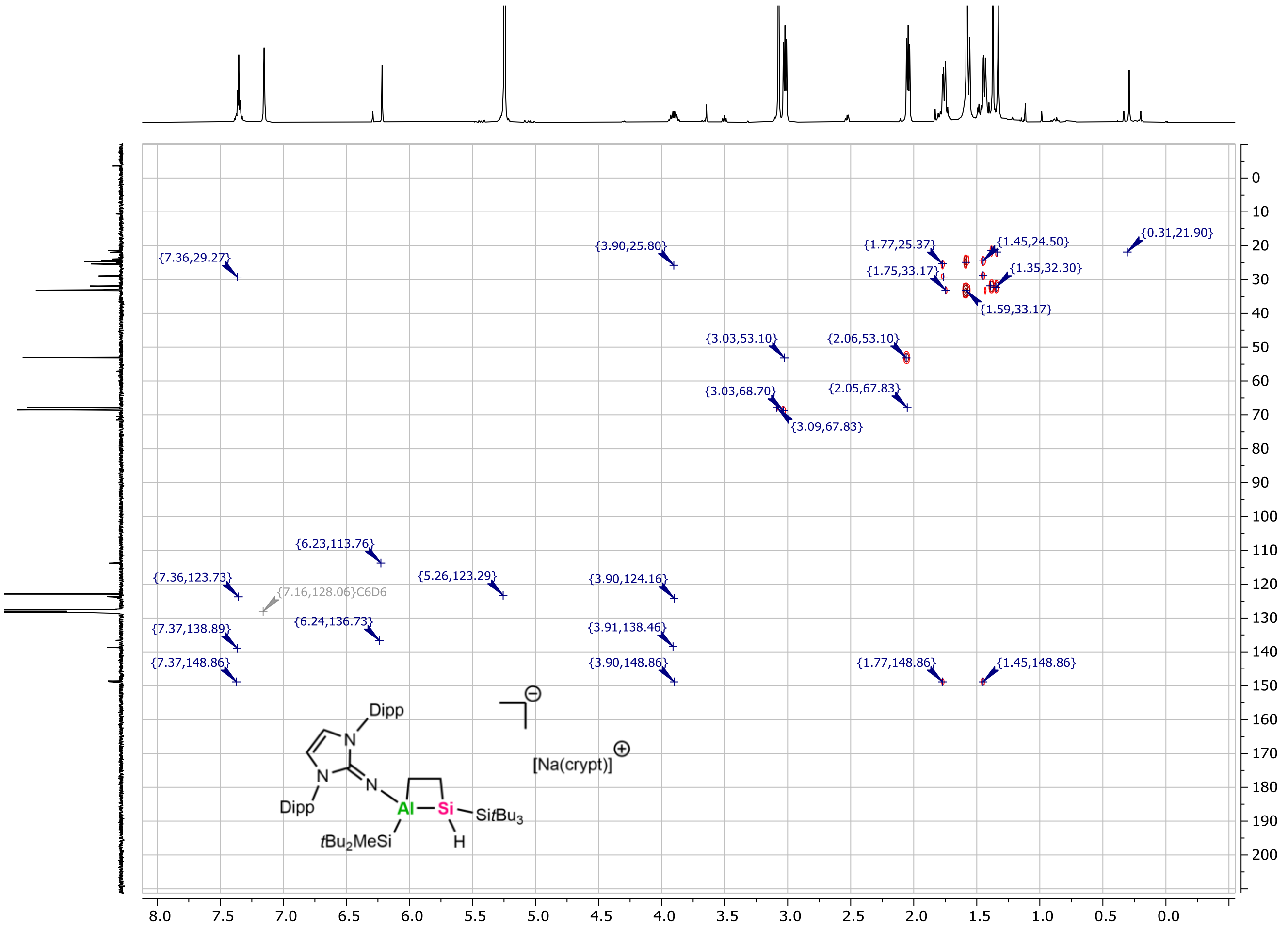

Figure S11: $^{1}H^{13}C$ HMBC NMR spectrum of **2b** with $^{13}C\{^{1}H\}$ spectrum as vertical trace and ^{1}H spectrum as horizontal trace.

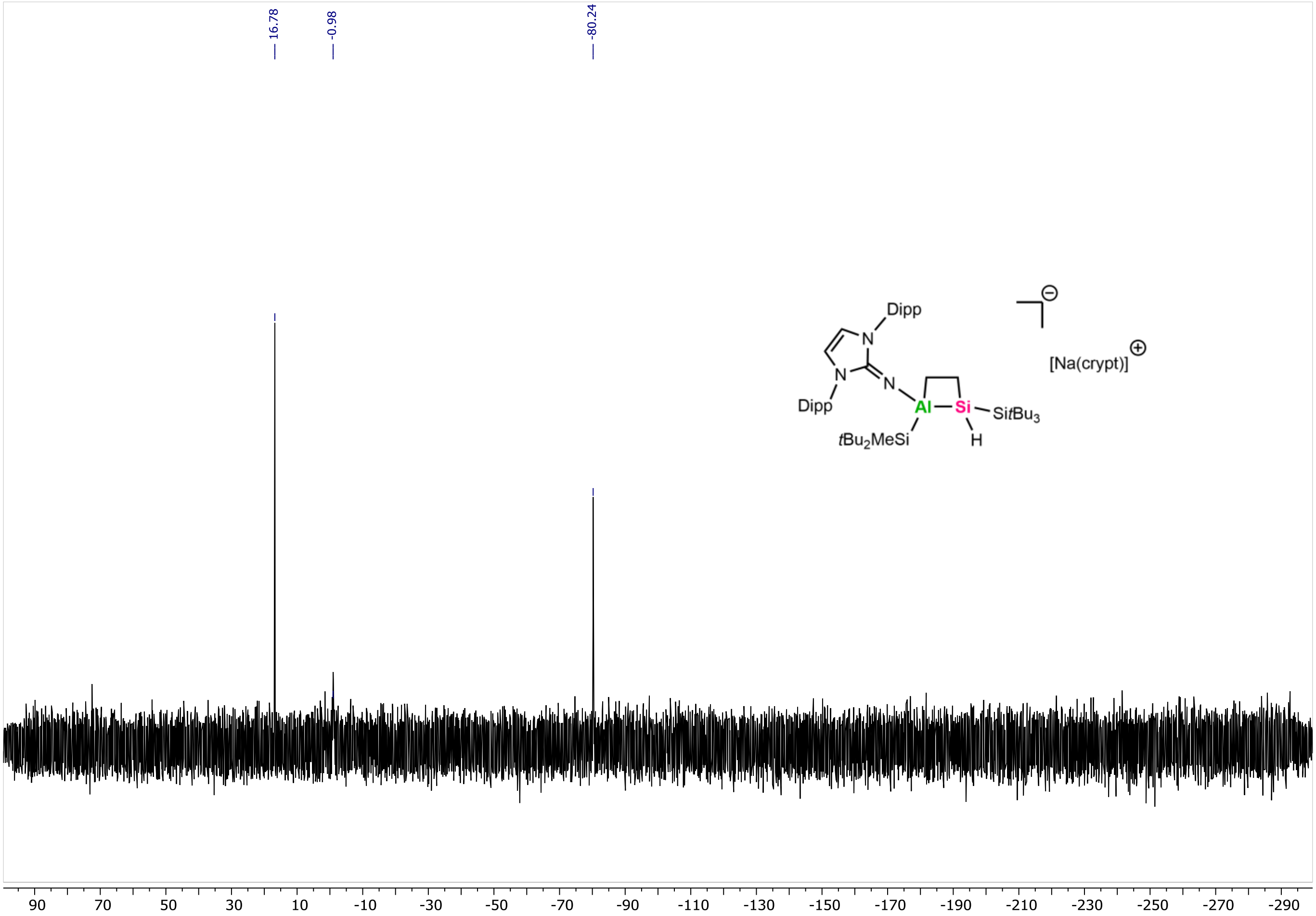

Figure S12: $^{29}Si\{^{1}H\}$ NMR spectrum (99.4 MHz, C_6D_6) of **2b**.

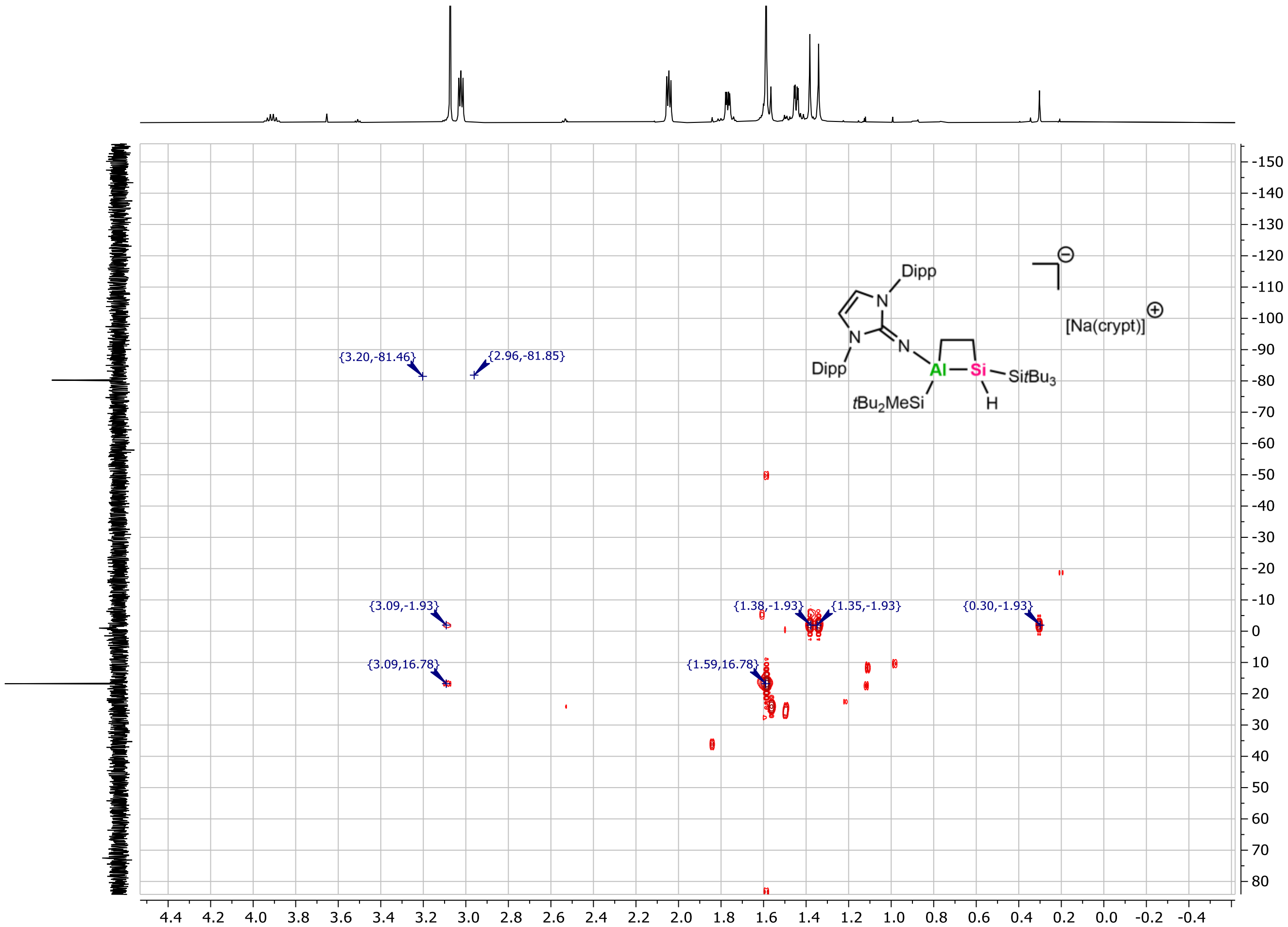

Figure S13: $^{1}H^{29}Si$ HMBC NMR spectrum of **2b** with $^{29}Si\{^{1}H\}$ spectrum as vertical trace and ^{1}H spectrum as horizontal trace. The spectrum is referenced to the ***H*Si*Si*^{t}Bu$_3$** cross-peak according to the ^{1}H and $^{29}Si\{^{1}H\}$ spectral data.

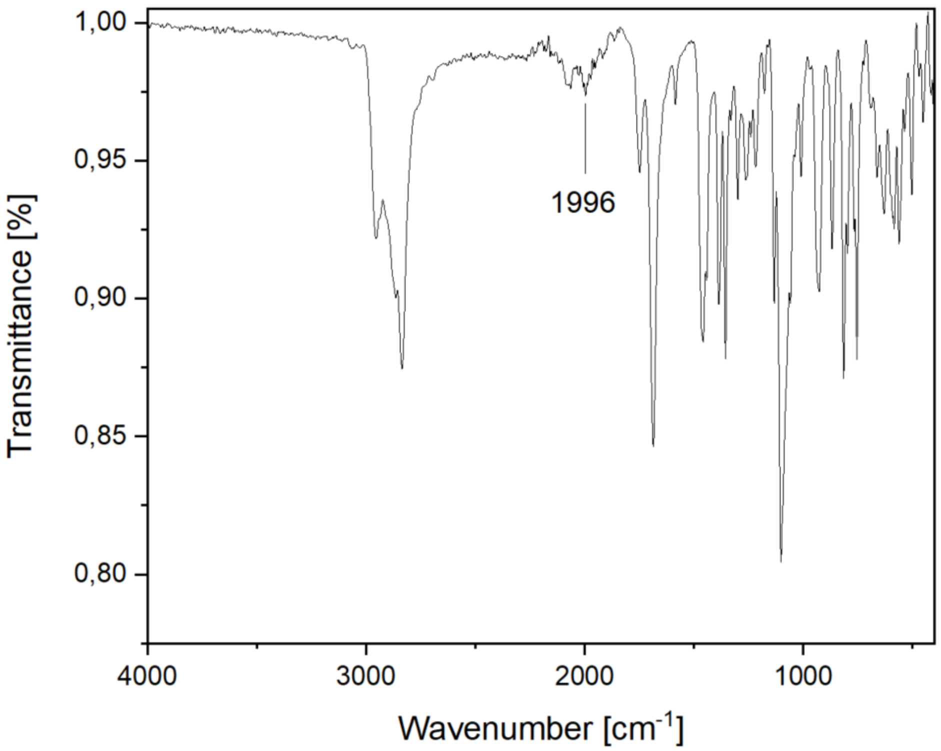

Figure S14: Solid-state FT-IR spectrum of **2b**. The position of the Si-H band is marked.

Synthesis of Styrene Cycloaddition Product (3a)

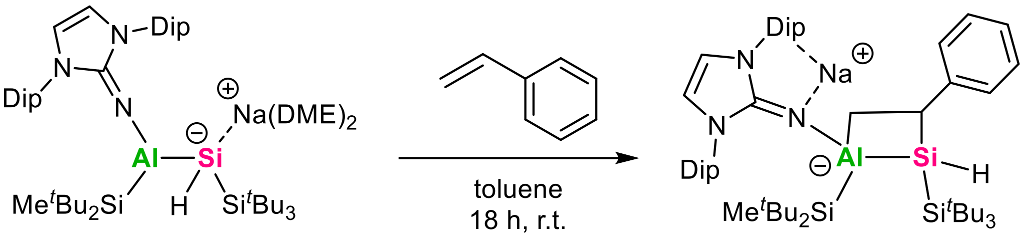

A solution of styrene (27.0 µL, 236 µmol, 1.2 eq) in toluene (1 mL) was added dropwise to an orange solution of alumanyl silanide **1a** (200 mg, 196 µmol, 1.0 eq) in toluene (5 mL) with stirring at r.t.. The reaction mixture was stirred at r.t. for 18 h, during which the color changed from orange to yellow. Then all volatiles were removed in vacuum to afford a yellow residue which was washed with *n*-pentane (2 x 4 mL) and dried to obtain **3a** as off-white powder (148 mg, **67%**).

^{1}H NMR (400.1 MHz, C_6D_6): δ = 7.22 (m, 2H, styrene)[a], 7.07 (m, 6H, DipH), 7.01 (m, 4H, styrene), 6.81 (t, ^{3}J = 7 Hz, 1H, C*H*-styrene), 6.25 (d, ^{3}J = 8 Hz, 2H, CH_2-styrene), 5.98 (s, 2H, NC*H*), 3.59 (sept, ^{3}J = 7 Hz, 2H, C*H*-^{i}Pr), 3.30 (bs, 2H, C*H*-^{i}Pr), 3.03 (s, 1H, Si*H*), 1.50 (d, ^{3}J = 7 Hz, 6H, CH_3-^{i}Pr), 1.45 (d, ^{3}J = 7 Hz, 6H, CH_3-^{i}Pr), 1.36 (s, 27H, Si^{t}Bu$_3$), 1.14 (m, 12H, CH_3-^{i}Pr), 1.02 (s, 9H, Si^{t}Bu$_2$), 0.96 (s, 9H, Si^{t}Bu$_2$), 0.06 (s, 3H, SiCH_3). **^{13}C{^{1}H} NMR** (100.6 MHz, C_6D_6): δ = 155.2 (*C*H$_{aryl}$-styrene), 149.3 (NHI-C_{aryl}), 143.7 (NHI-C_{aryl}), 136.7 (NHI-C_{aryl}), 127.5 (*C*H$_{aryl}$-styrene), 125.3 (CH_2-styrene), 119.6 (*C*H-styrene), 114.1 (N*C*H), 32.4 (Si^{t}Bu$_3$), 31.2 (Si^{t}Bu$_2$), 31.0 (Si^{t}Bu$_2$), 29.1 (*C*H-^{i}Pr), 28.6 (*C*H-^{i}Pr), 26.0 (CH_3-^{i}Pr), 25.7 (CH_3-^{i}Pr), 24.3 (Si^{t}Bu$_3$), 23.8 (CH_3-^{i}Pr), 23.4 (CH_3-^{i}Pr), 22.7 (CH_3-^{i}Pr), 22.3 (CH_3-^{i}Pr), 20.6 (Si^{t}Bu$_2$), 20.5 (Si^{t}Bu$_2$), -3.8 (SiCH_3). **^{29}Si{^{1}H} NMR** (99.4 MHz, C_6D_6): δ = 19.1 (*Si*^{t}Bu$_3$), 1.0 (*Si*^{t}Bu$_2$), -91.5 (*Si*H).

FT-IR (neat, cm^{-1}): $\tilde{\nu}$ = 1967 (s) (Si-H).

Melting point: 172.0°C

Notes: **a**: The signal overlaps with the signal of residual deuterated C_6D_6.

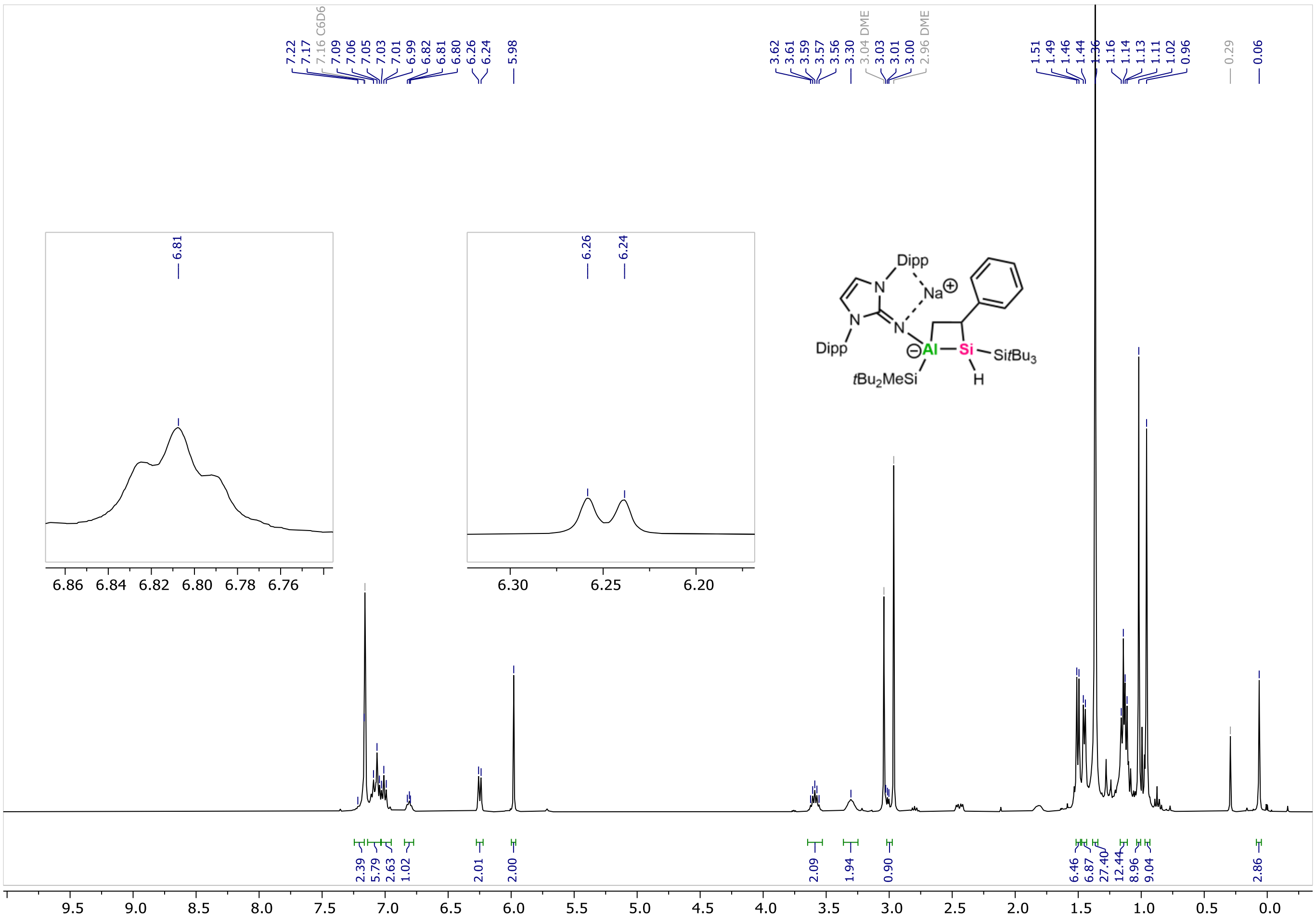

Figure S15: ^{1}H NMR spectrum (400.1 MHz, C_6D_6) of **3a** including "zoom in" on the C***H***-styrene and the C***H***$_2$-styrene signal. Residual solvent and DME signals are marked in grey.

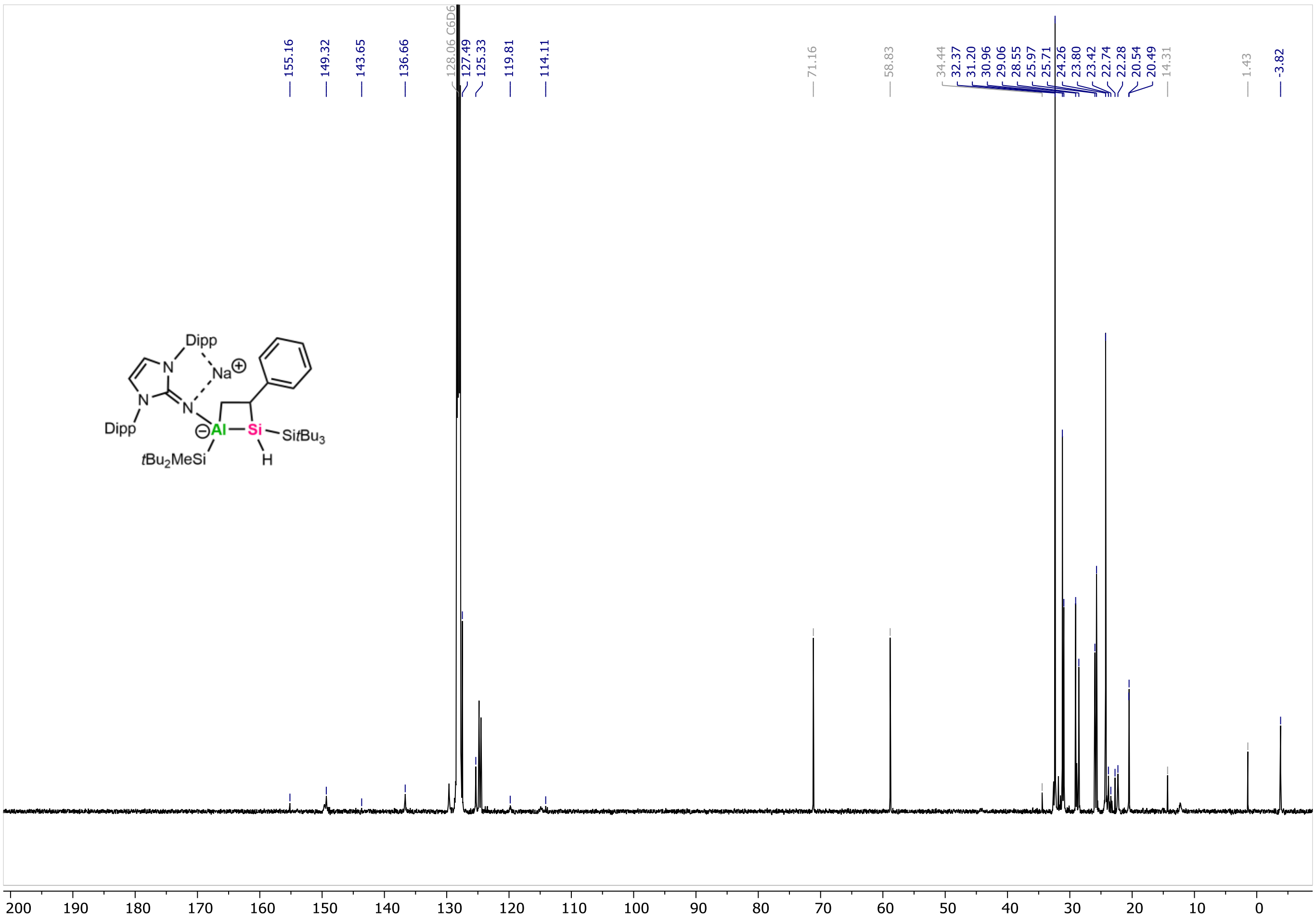

Figure S16: ^{13}C {^{1}H} NMR spectrum (100.6 MHz, C_6D_6) of **3a**. Residual solvent and DME signals are marked in grey.

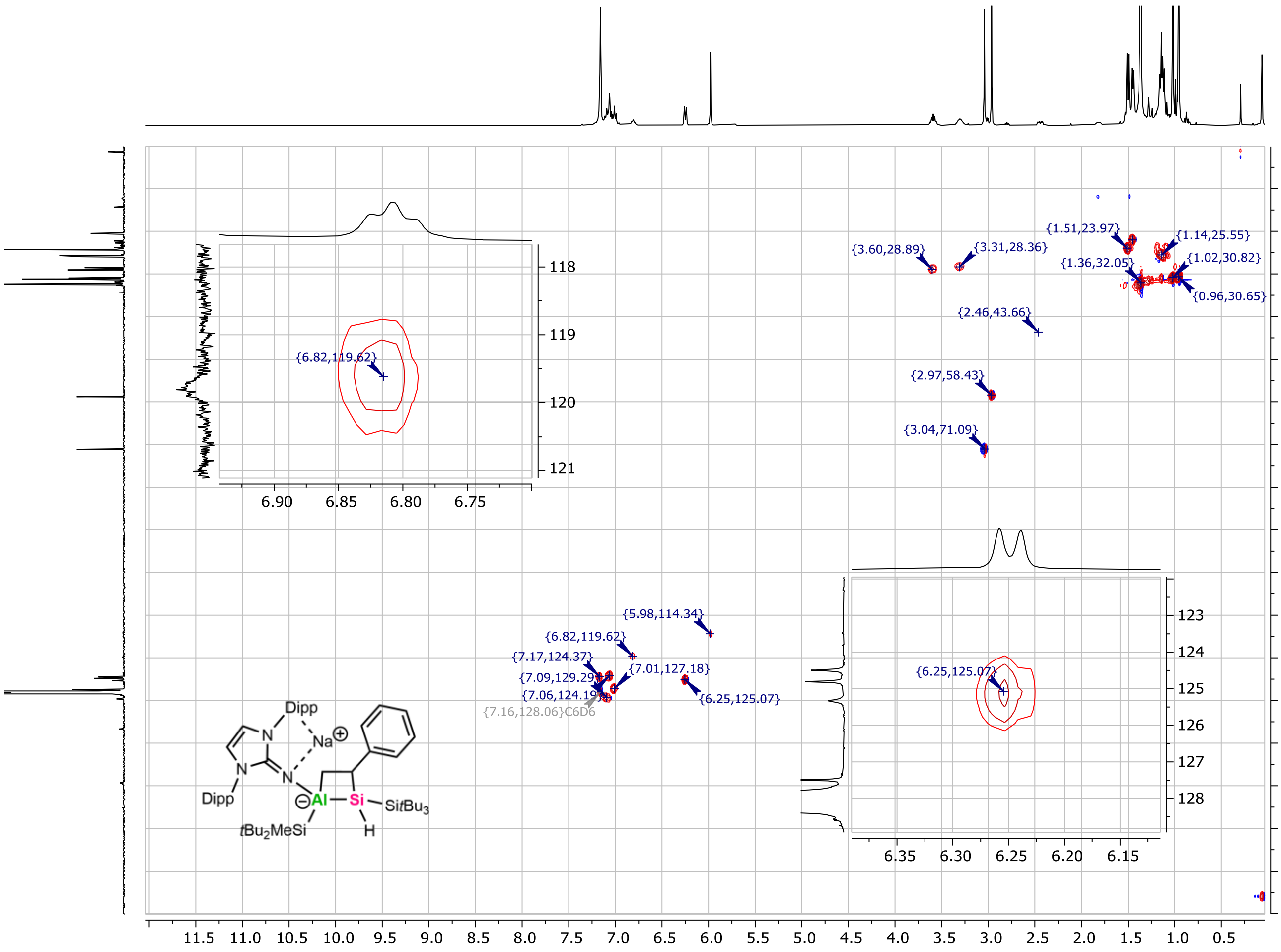

Figure S17: $^{1}H^{13}C$ HSQC NMR spectrum of **3a** with $^{13}C\{^{1}H\}$ spectrum as vertical trace and ^{1}H spectrum as horizontal trace including “zoom in” on the CH_2-styrene and CH-styrene signal.

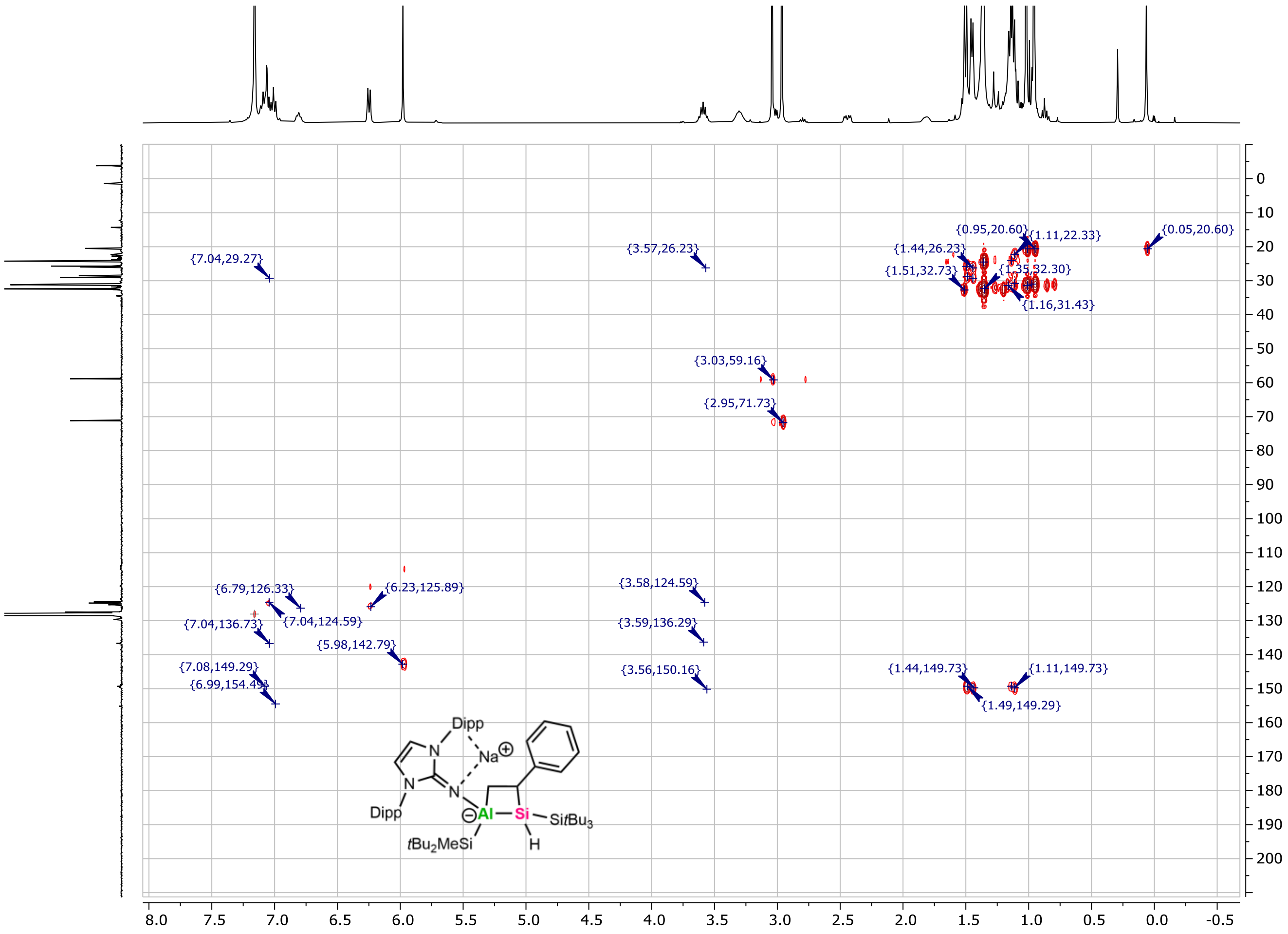

Figure S18: $^{1}H^{13}C$ HMBC NMR spectrum of **3a** with $^{13}C\{^{1}H\}$ spectrum as vertical trace and ^{1}H spectrum as horizontal trace.

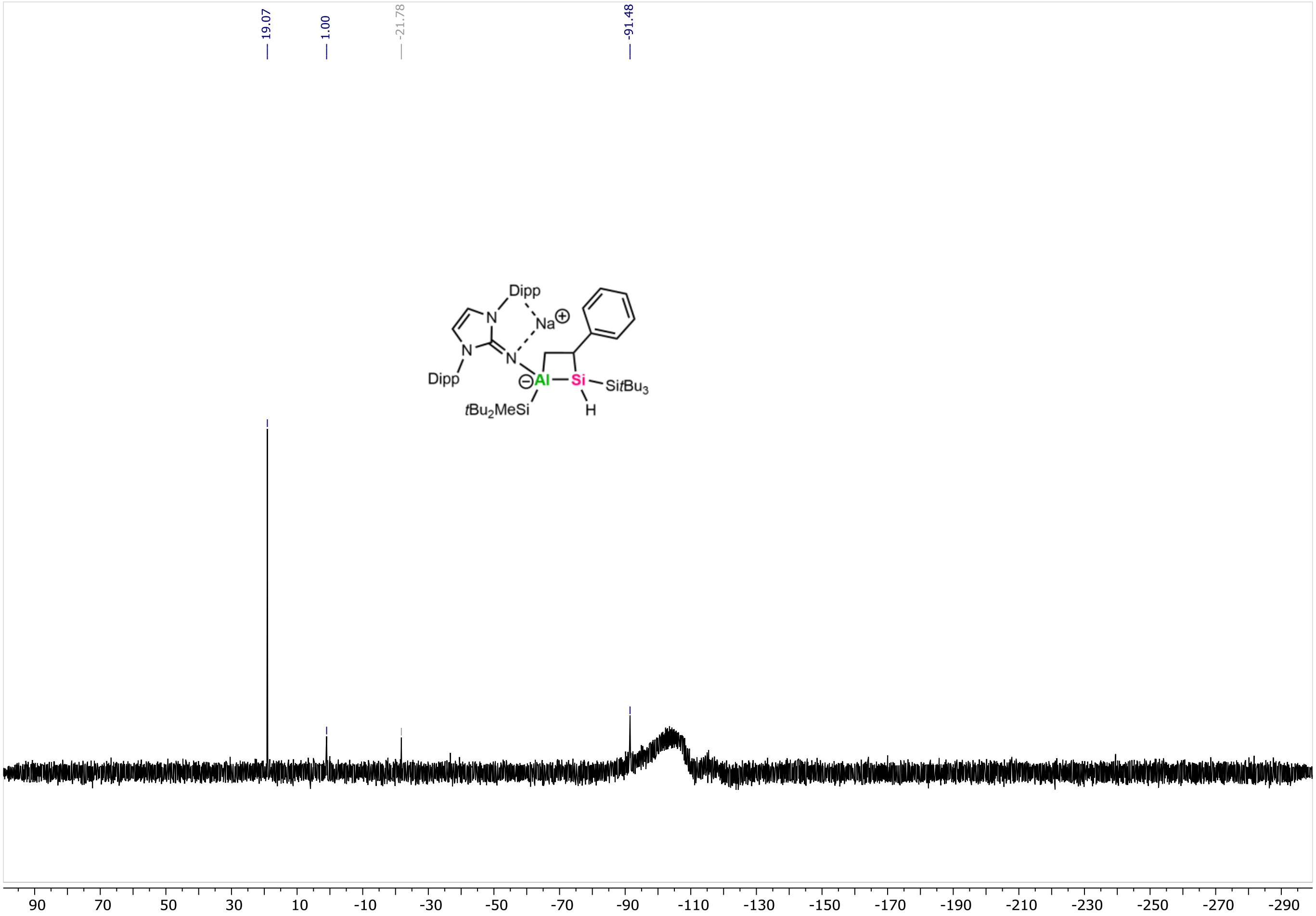

Figure S19: $^{29}Si\{^{1}H\}$ NMR spectrum (99.4 MHz, C_6D_6) of **3a**.

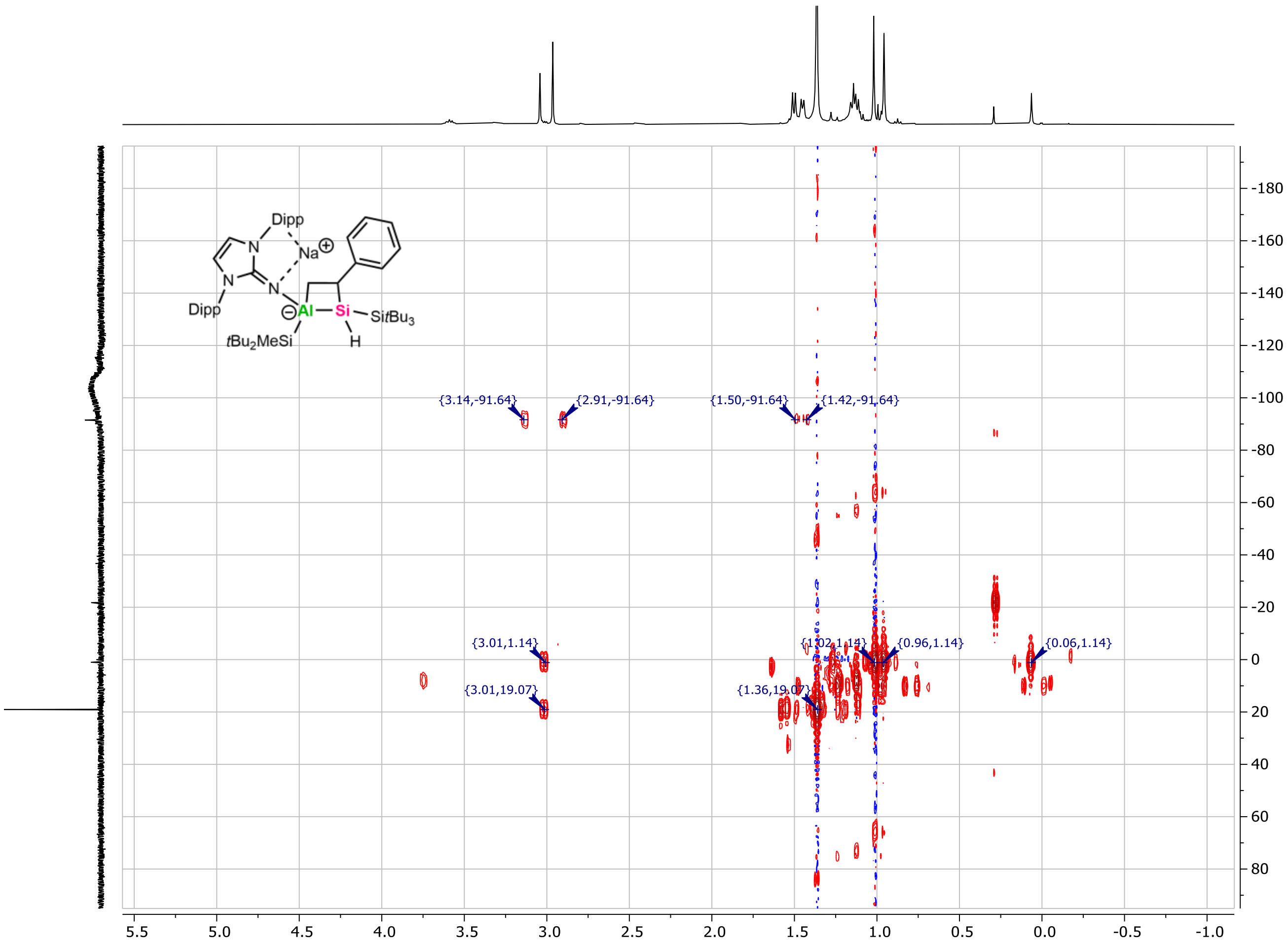

Figure S20: $^{1}H^{29}Si$ HMBC NMR spectrum of **3a** with $^{29}Si\{^{1}H\}$ spectrum as vertical trace and ^{1}H spectrum as horizontal trace. The spectrum is referenced to the ***H***Si***Si***^{t}Bu$_3$ cross-peak according to the ^{1}H and $^{29}Si\{^{1}H\}$ spectral data.

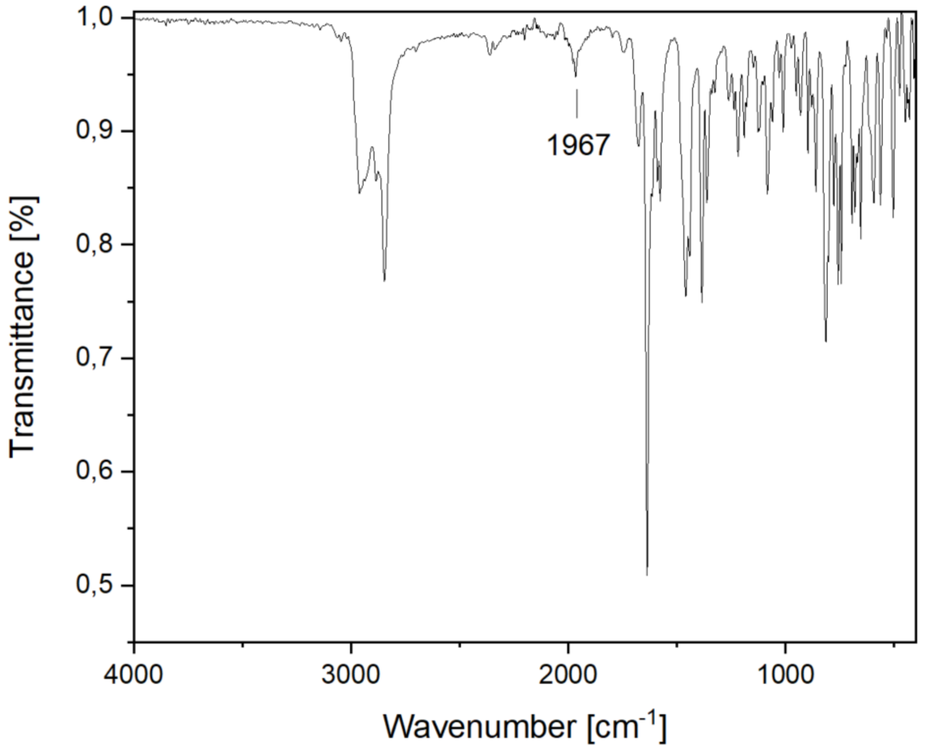

Figure S21: Solid-state FT-IR spectrum of **3a**. The position of the Si-H band is marked.

Synthesis of Cryptand stabilized Styrene Cycloaddition Product (3b)

Method A:

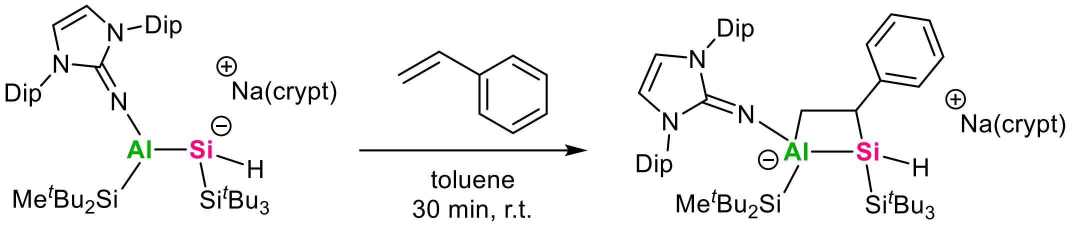

A solution of styrene (4.62 µL, 40.3 µmol, 1 eq) in toluene (1 mL) was added dropwise to an orange solution of aluminata-silene **1b** (29.0 mg, 40.3 µmol, 1 eq) in toluene (3 mL) with stirring. The reaction was stirred at r.t. for 30 min until the solution completely decolorized. After that, all volatiles were removed under reduced pressure and the residue was washed with *n*-pentane (2 mL) and dried to obtain **3b** as off-white powder (24.3 mg, **48%**).

Method B:

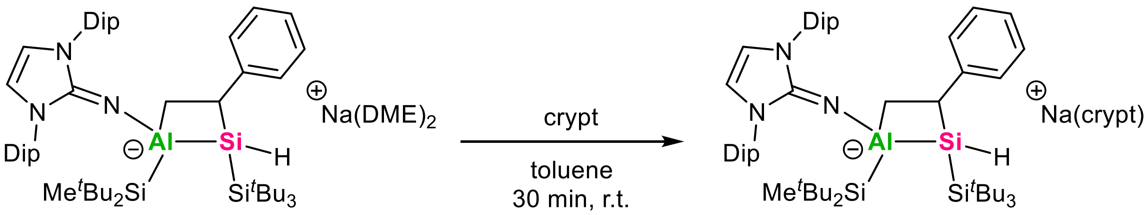

Cycloaddition product **3a** (24.7 mg, 22.0 µmol, 1 eq) was dissolved in toluene (3 mL). After addition of [2.2.2]cryptand (8.28 mg, 22.0 µmol, 1 eq), the reaction was stirred for 30 min at r.t.. After that, all volatiles were removed under reduced pressure. The residue was washed with *n*-pentane (2 mL) and dried to obtain **3b** as off-white powder (24.8 mg, **86%**).

^{1}H NMR (400.1 MHz, C_6D_6): δ = 7.46 (m, 2H, styrene), 7.36 (t, ^3J = 8 Hz, 4H, DipH), 7.31 (m, 3H, styrene), 7.10 (t, ^3J = 8 Hz, 2H, DipH), 6.77 (t, ^3J = 7 Hz, 1H, C*H*-styrene), 6.66 (d, ^3J = 8 Hz, 2H, CH_2-styrene), 6.21 (s, 2H, NC*H*), 4.01 (sept, ^3J = 7 Hz, 2H, C*H*-^{i}Pr), 3.88 (bs, 2H, C*H*-^{i}Pr), 2.99 (s, 12H, OCH_2-crypt), 2.96 (t, ^3J = 6 Hz, 12H, OCH_2-crypt), 1.98 (t, ^3J = 6 Hz, 12H, NCH_2-crypt), 1.79 (d, ^3J = 7 Hz, 6H, CH_3-^{i}Pr), 1.76 (d, ^3J = 7 Hz, 6H, CH_3-^{i}Pr), 1.66 (s, 27H, Si^{t}Bu$_3$), 1.47 (d, ^3J = 7 Hz, 6H, CH_3-^{i}Pr), 1.24 (s, 9H, Si^{t}Bu$_2$), 1.05 (s, 9H, Si^{t}Bu$_2$), 0.32 (s, 3H, SiCH_3). **^{13}C{^{1}H} NMR** (100.6 MHz, C_6D_6): δ = 158.4 (*C*H$_{aryl}$-styrene), 149.4 (NHI-*C*$_{aryl}$), 148.9 (NHI-*C*$_{aryl}$), 139.0 (NHI-*C*$_{aryl}$), 137.0 (NHI-*C*$_{aryl}$), 127.2 (NHI-*C*$_{aryl}$), 125.3 (*C*H$_2$-styrene), 124.0 (*C*H$_{aryl}$-styrene), 123.8 (*C*H$_{aryl}$-styrene), 117.4 (*C*H$_2$-styrene), 114.5 (N*C*H), 68.4 (O*C*H$_2$-crypt), 67.6 (O*C*H$_2$-crypt), 52.7 (N*C*H$_2$-crypt), 33.1 (Si^{t}Bu$_3$), 32.5 (Si^{t}Bu$_2$), 31.0 (Si^{t}Bu$_2$), 29.1 (*C*H-^{i}Pr), 29.0 (*C*H-^{i}Pr), 26.2 (*C*H$_3$-^{i}Pr), 25.6 (*C*H$_3$-^{i}Pr), 24.8 (*C*H$_3$-^{i}Pr), 24.7 (Si^{t}Bu$_3$), 24.1 (*C*H$_3$-^{i}Pr), 21.2 (Si^{t}Bu$_2$), 21.0 (Si^{t}Bu$_2$), -3.2 (Si*C*H$_3$). **^{29}Si{^{1}H} NMR** (99.4 MHz, C_6D_6): δ = 19.3 (*Si*^{t}Bu$_3$), 0.2 (*Si*^{t}Bu$_2$), -91.2 (*Si*H).

FT-IR (neat, cm^{-1}): $\tilde{\nu}$ = 1994 (s) (Si-H).

Melting point: 71.5°C

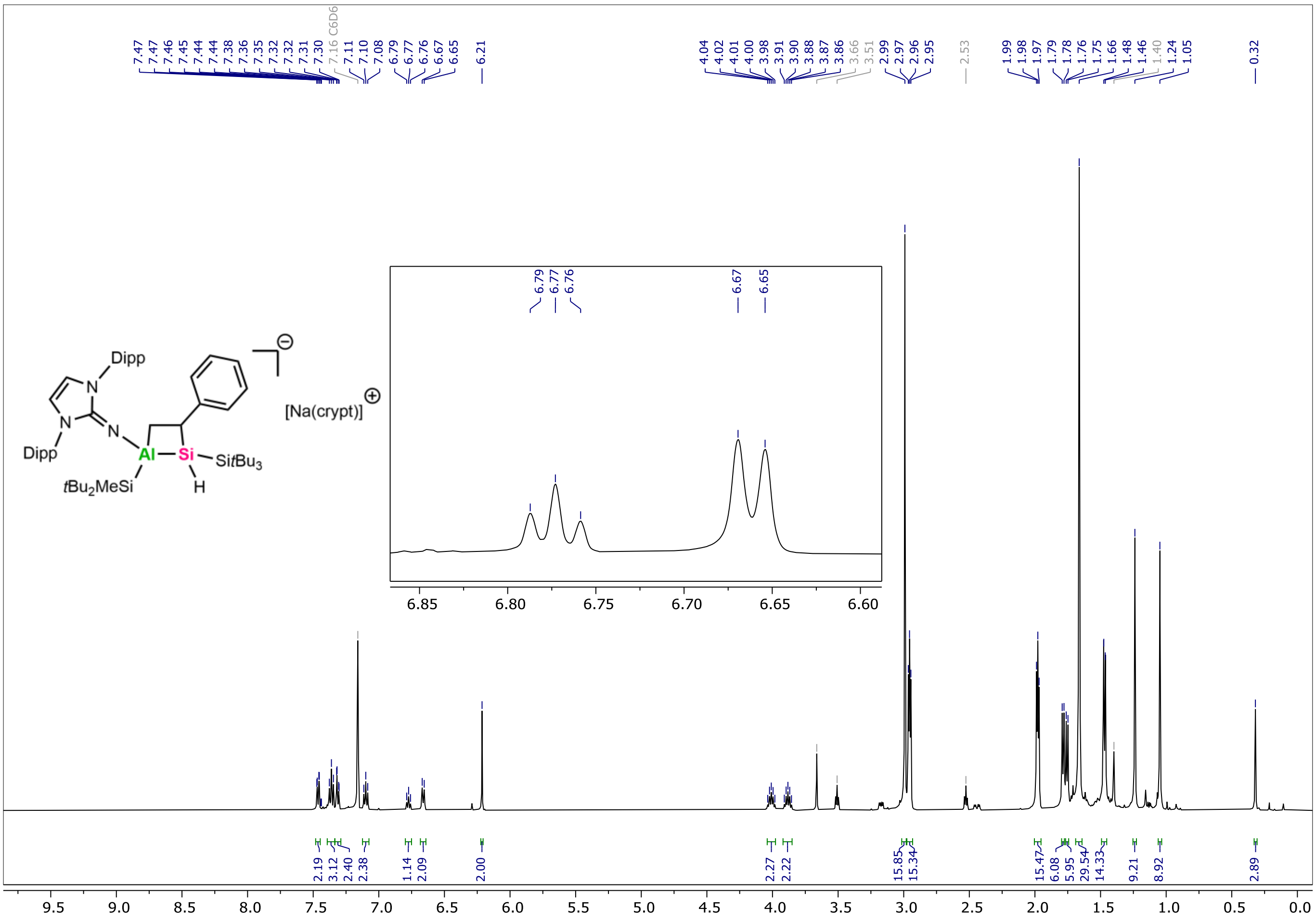

Figure S22: ^{1}H NMR spectrum (400.1 MHz, C_6D_6) of **3b** including "zoom in" on the C***H***-styrene and the C***H***$_2$-styrene.

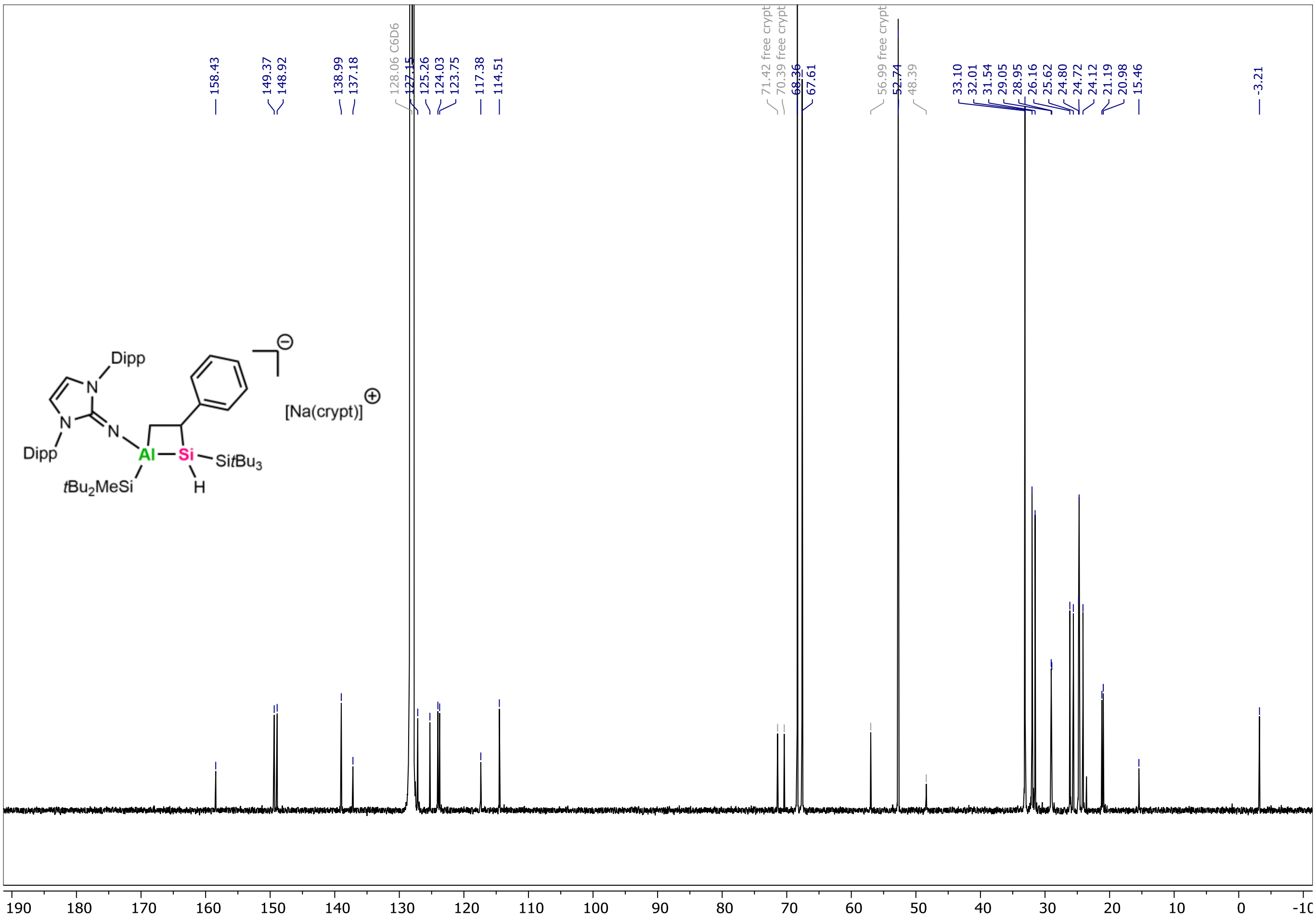

Figure S23: ^{13}C {^{1}H} NMR spectrum (100.6 MHz, C_6D_6) of **3b**.

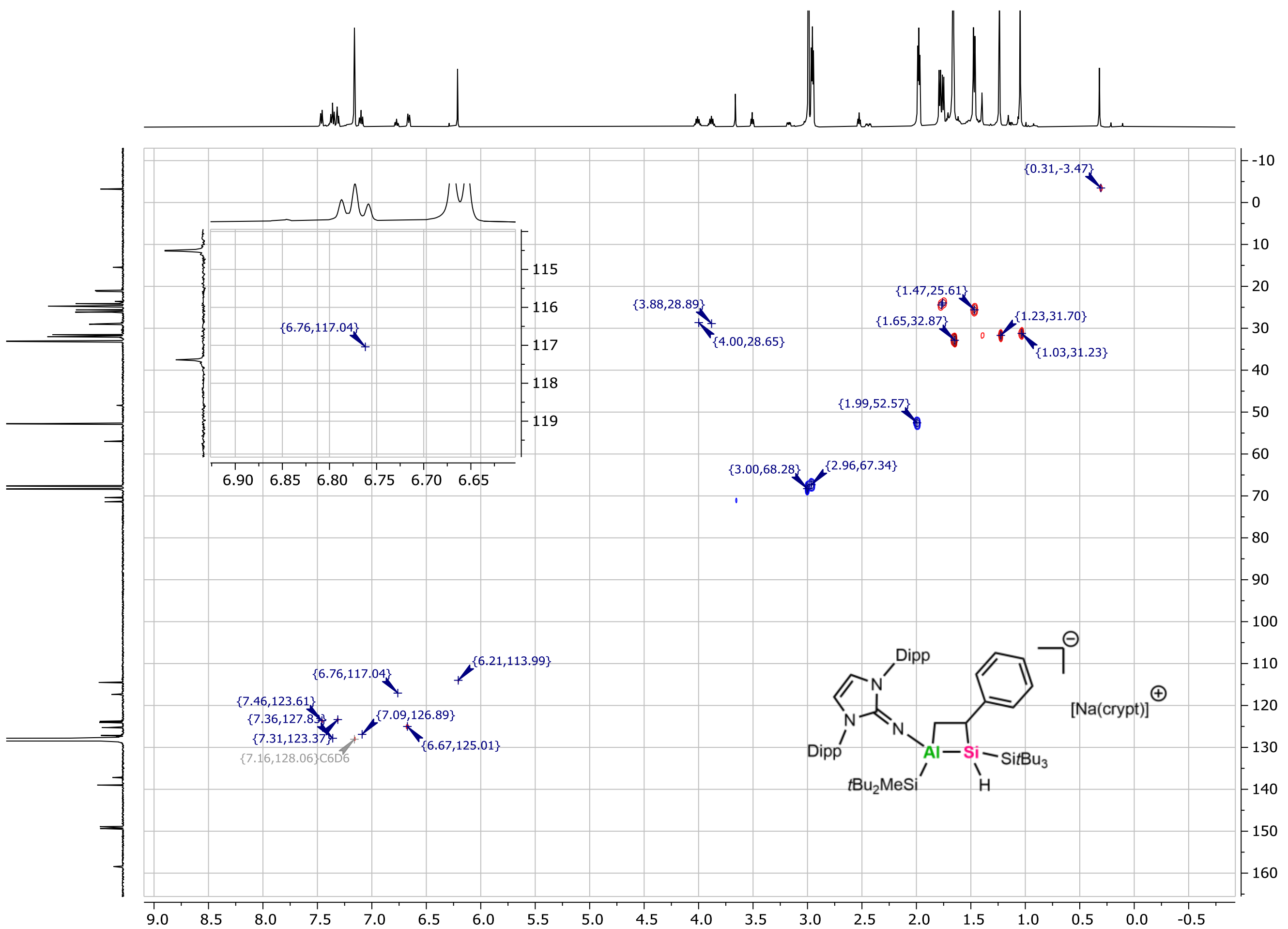

Figure S24: $^1H^{13}C$ HSQC NMR spectrum of **3b** with $^{13}C\{^1H\}$ spectrum as vertical trace and ^1H spectrum as horizontal trace including "zoom in" on the CH-styrene signal.

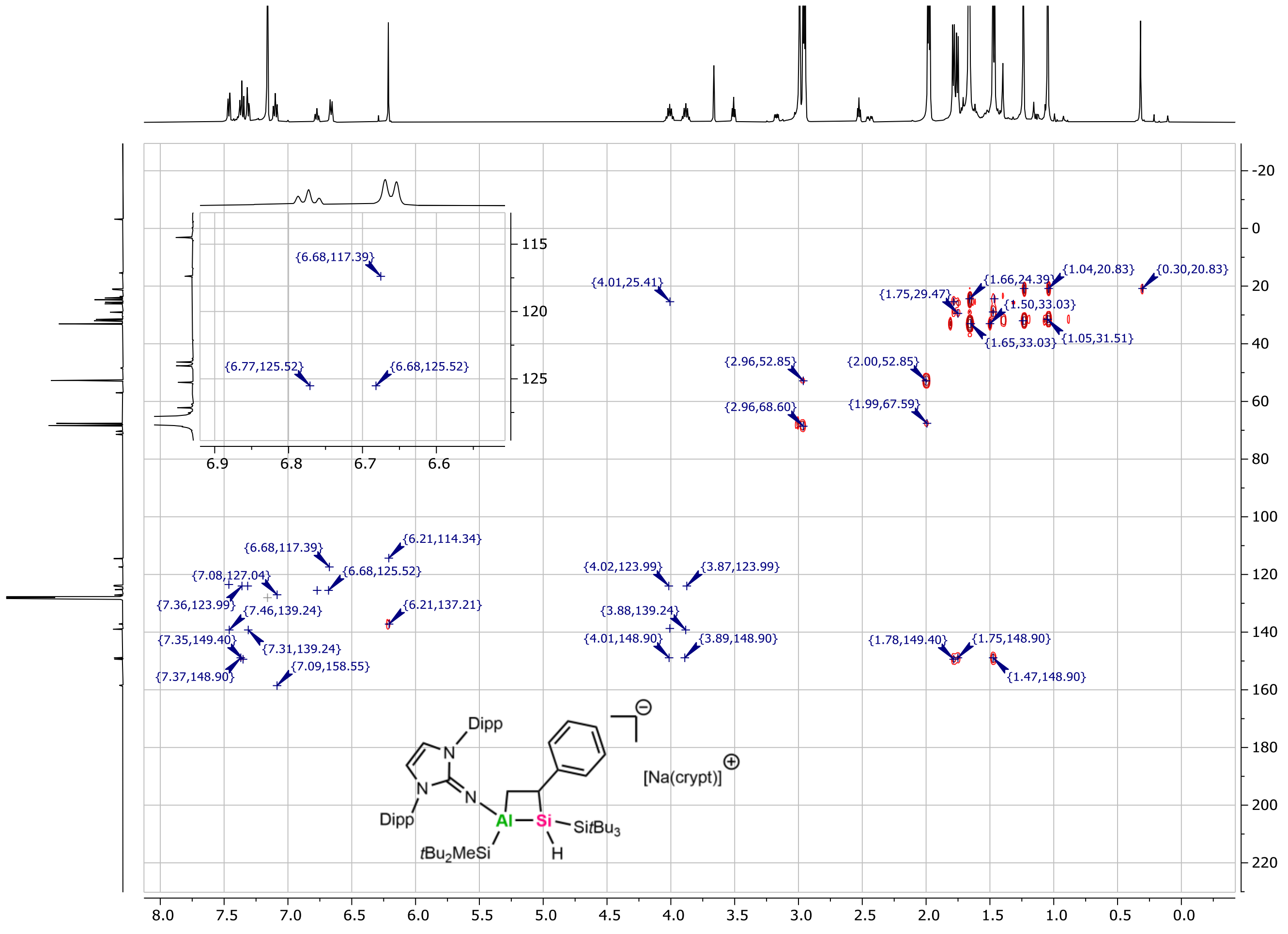

Figure S25: $^1H^{13}C$ HMBC NMR spectrum of **3b** with $^{13}C\{^1H\}$ spectrum as vertical trace and ^1H spectrum as horizontal trace including "zoom in" on the CH_2-styrene and the CH-styrene signal.

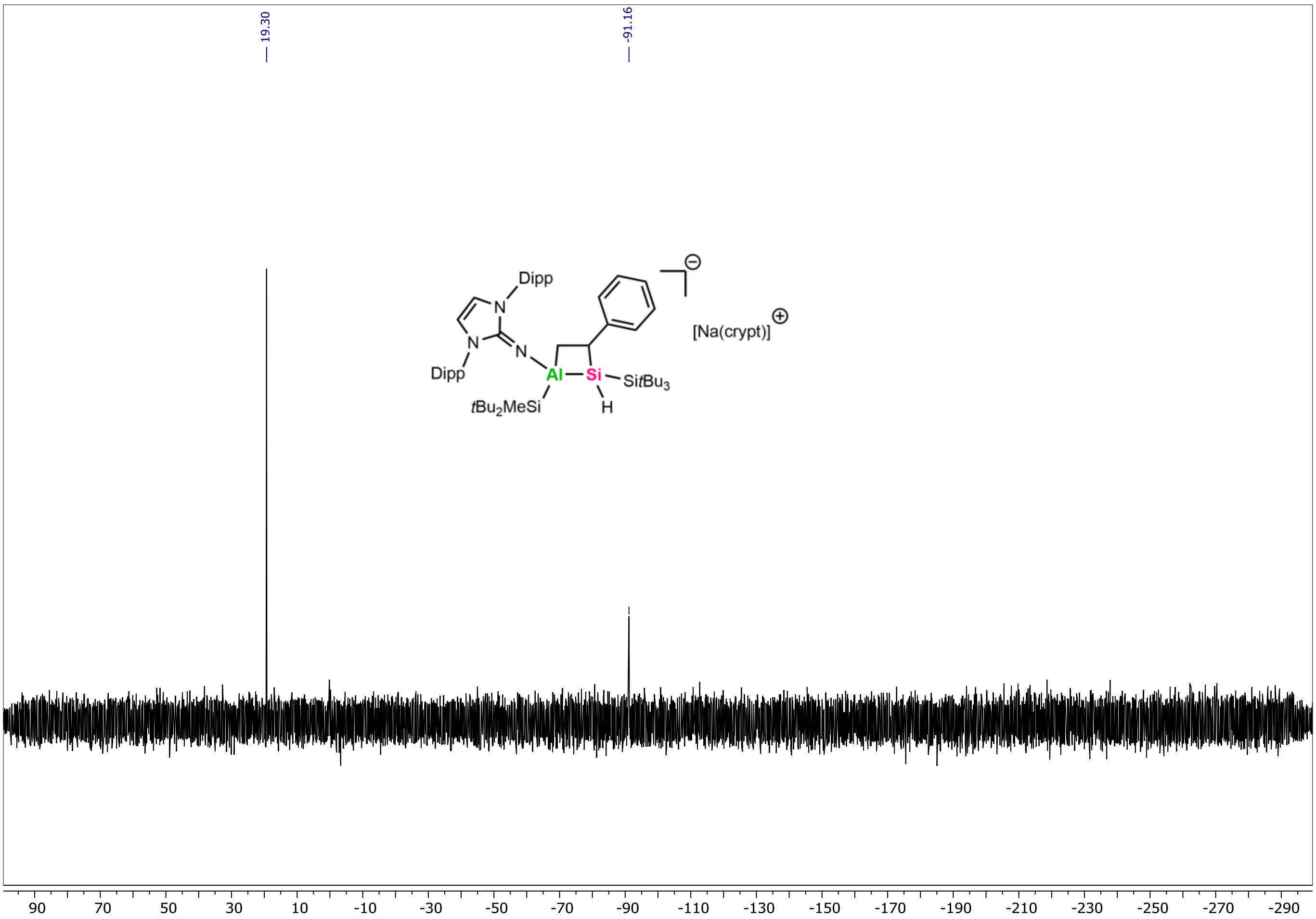

Figure S26: $^{29}Si\{^{1}H\}$ NMR spectrum (99.4 MHz, C_6D_6) of **3b**.

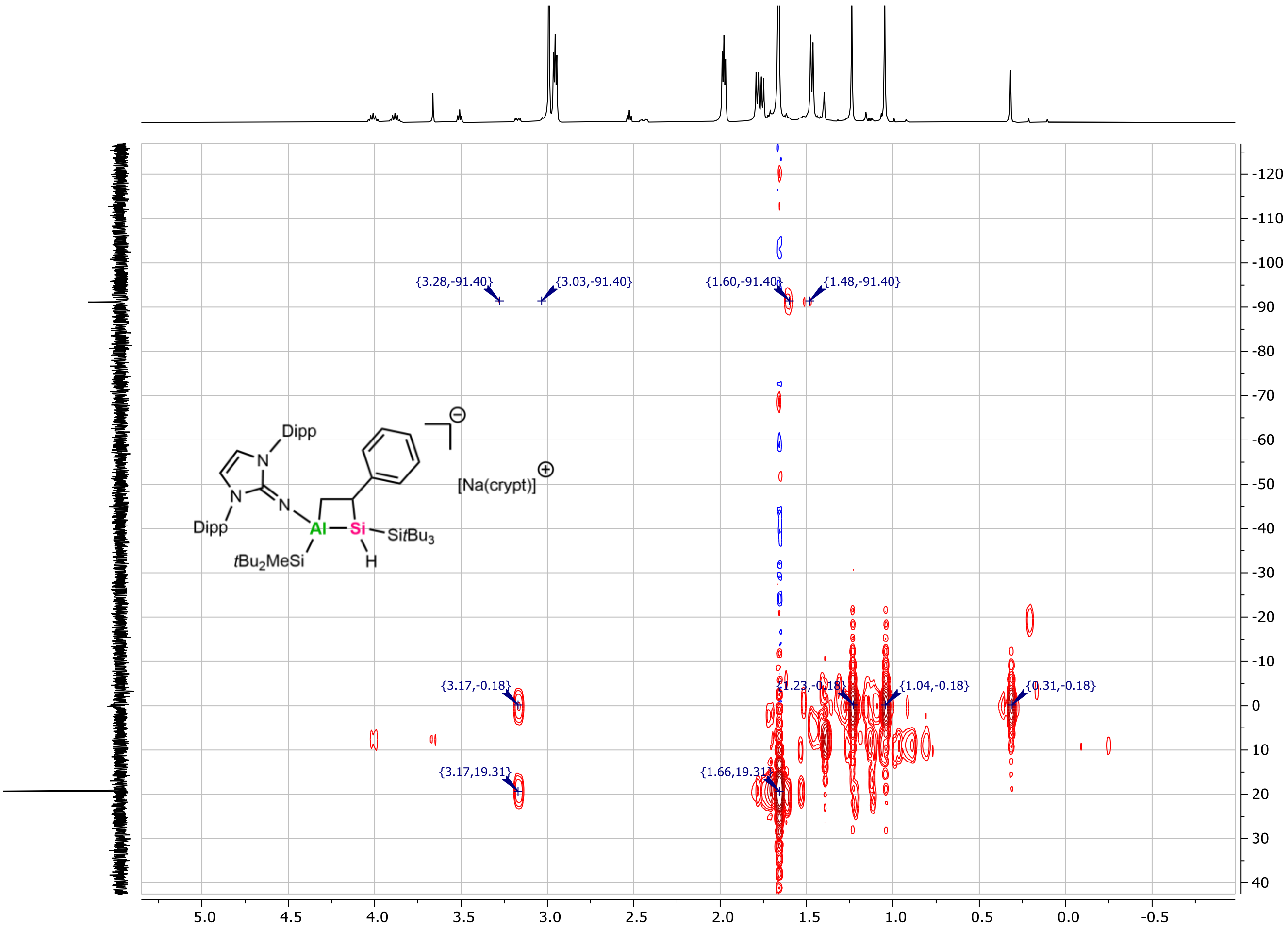

Figure S27: $^{1}H^{29}Si$ HMBC NMR spectrum of **3b** with $^{29}Si\{^{1}H\}$ spectrum as vertical trace and ^{1}H spectrum as horizontal trace. The spectrum is referenced to the ***H*Si*Si*** $^{t}Bu_3$ cross-peak according to the ^{1}H and $^{29}Si\{^{1}H\}$ spectral data.

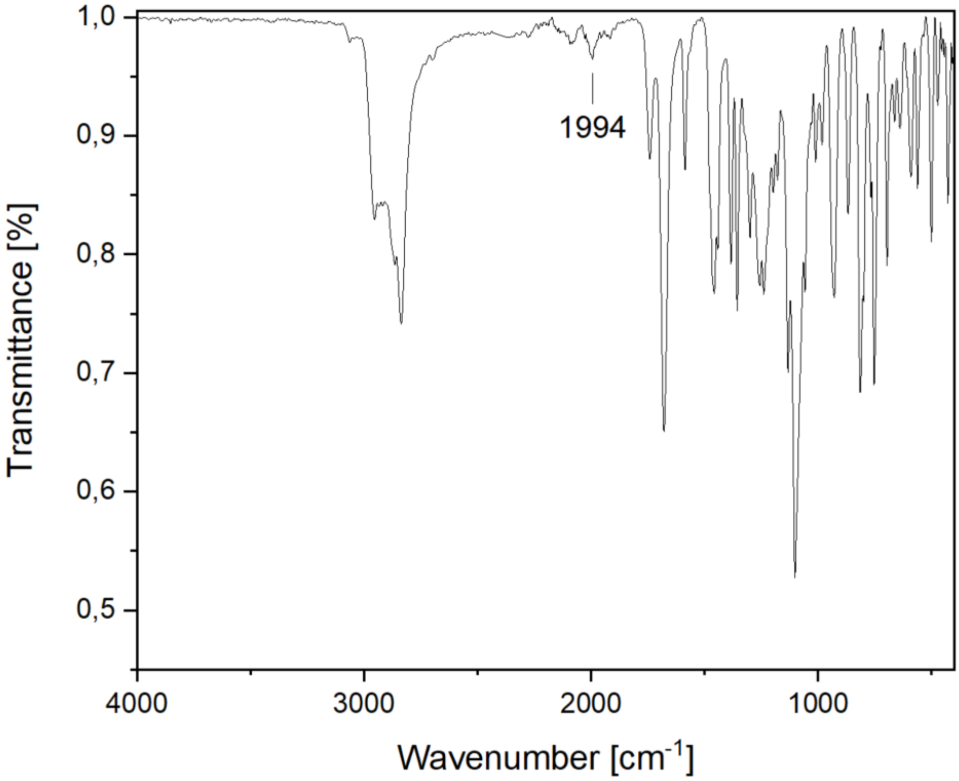

Figure S28: Solid-state FT-IR spectrum of **3b**. The position of the Si-H band is marked.

Synthesis of Carbon Dioxide Cycloaddition Product (4)

Method A (NMR scale):

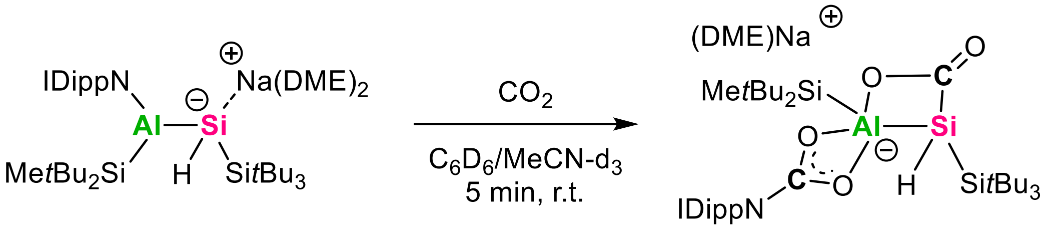

1a **4**

In a J-Young NMR tube, alumanyl silanide **1a** (11.0 mg, 10.8 µmol, 1 eq) was dissolved in C_6D_6 (0.3 mL). After freezing the reaction mixture and removing the argon atmosphere in dynamic vacuum, CO_2 (1.5 bar) was added to the frozen reaction mixture. After thawing and leaving the reaction mixture for 5 min at r.t., the reaction color changed from orange to colorless, with **4** precipitating as fine white powder from the reaction solution. Subsequently, MeCN-d_3 (0.1 mL) was added to dissolve **4**. NMR analysis shows the selective formation of **4**.

Method B (reaction in toluene):

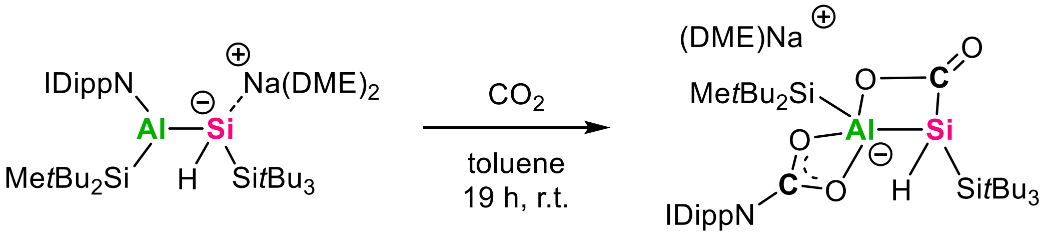

1a **4**

A solution of alumanyl silanide **1a** (100 mg, 98.2 µmol, 1 eq) in toluene (5 mL) was frozen with subsequent removal of the argon atmosphere in dynamic vacuum. After pressurizing the reaction mixture with CO_2 (1.5 bar), the color changed from an initially orange to colorless over the course of 5 min at r.t., with **4** precipitating as fine white powder from the reaction solution. The reaction was stirred for an additional 19 h to ensure full conversion. After drying in dynamic vacuum, the white solid was washed with toluene (1 mL) and pentane (1 mL). After drying in dynamic vacuum, the product was obtained in form of a white powder (70.7 mg, **68%**). NMR analysis shows ill-defined signals, possibly resulting from partial decomposition of **4** in solid state.[a]

Notes: **a**: Ill-defined signals can be found at δ = 5.99, 3.00 and 0.13 ppm.

Method C (neat reaction):

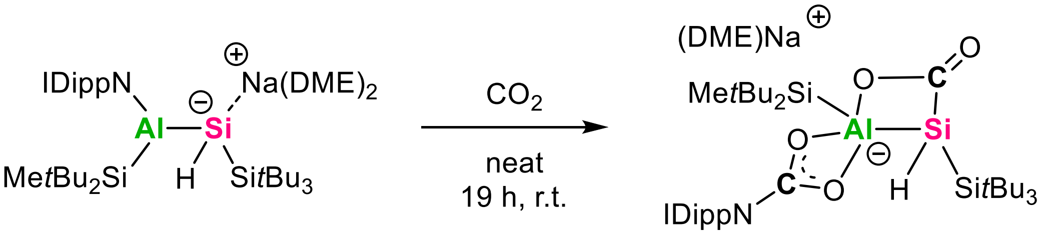

1a **4**

Alumanyl silanide **1a** (10.0 mg, 9.82 µmol, 1 eq) was weighted into a J-Young NMR tube. After removing the argon atmosphere in dynamic vacuum, CO_2 (1.5 bar) was added. After 1 h at r.t., the color changed from orange to colorless. After leaving the white powder for an additional 19 h at r.t. to ensure complete conversion, the product was obtained in quantitative yield. NMR analysis shows ill-defined signals, possibly resulting from partial decomposition of **4** in solid state.[a]

Notes: **a**: Ill-defined signals can be found at δ = 5.99, 3.00 and 0.13 ppm.

^{1}H NMR (400.1 MHz, C_6D_6): δ = 7.24-7.28 (t, ^{3}J = 7.7 Hz, 2H, Dipp-*H*), 7.1-7.13 (m, 2H, Dipp-*H*), 6.31 (s, 2H, NC*H*), 4.22 (s, 1H, Si*H*), 3.26 (s, 10H, C*H*$_2$-DME), 3.10 (s, 15H, C*H*$_3$-DME), 3.80-3.89 (sept, ^{3}J = 6.9 Hz, 2H, C*H*-^{i}Pr), 1.26-1.28 (d, ^{3}J = 6.8 Hz, 12H, C*H*$_3$-^{i}Pr), 1.20 (s, 27H, Si*t*Bu$_3$), 1.05-1.07 (d, ^{3}J = 6.9 Hz, 12H, C*H*$_3$-^{i}Pr), 0.97 (s, 18H, Si*t*Bu$_2$), -0.10 (s, 3H, SiC*H*$_3$). **^{13}C{^{1}H} NMR** (100.6 MHz, C_6D_6): δ = 188.4 (Si-*C*O$_2$), 162.2 (NHI-*C*O$_2$), 146.5 (NHI-*C*$_{aryl}$), 133.7 (*C*$_{aryl}$), 130.0 (*C*$_{aryl}$), 125.0 (free *C*O$_2$), 124.4 (NHI-*C*$_{aryl}$), 116.8 (N*C*H), 72.0 (*C*H$_3$-DME), 58.5 (*C*H$_2$-DME), 31.6 (Si*t*Bu$_3$), 30.3 (Si*t*Bu$_2$), 29.0 (Si*t*Bu$_2$), 24.4 (Mes-*C*H$_3$), 24.2 (Mes-*C*H$_3$), 23.5 (*C*H$_3$-^{i}Pr), 20.0 (Si*t*Bu$_2$), 0.40 (MeCN), -6.3 (Si*t*Bu$_2$). **^{29}Si{^{1}H} NMR** (99.4 MHz, C_6D_6): δ = 16.3 (*Si*^{t}Bu$_3$), 0.3 (*Si*^{t}Bu$_2$)[a], -75.8 (*Si*H).[b]

FT-IR (neat, cm^{-1}): $\tilde{\nu}$ = 2160 (m) (Si-CO$_2$), 1988 (w) (Si-H).

Melting point: 111.5°C

Notes: **a**: The assessment of the Si^{t}Bu$_2$ signal was verified by ^{1}H^{29}Si HMBC experiment. **b**: Although the NMR was taken in a mixture of C_6D_6 and MeCN-d_3 (3:1 mix.), the spectra were referenced using C_6D_6.

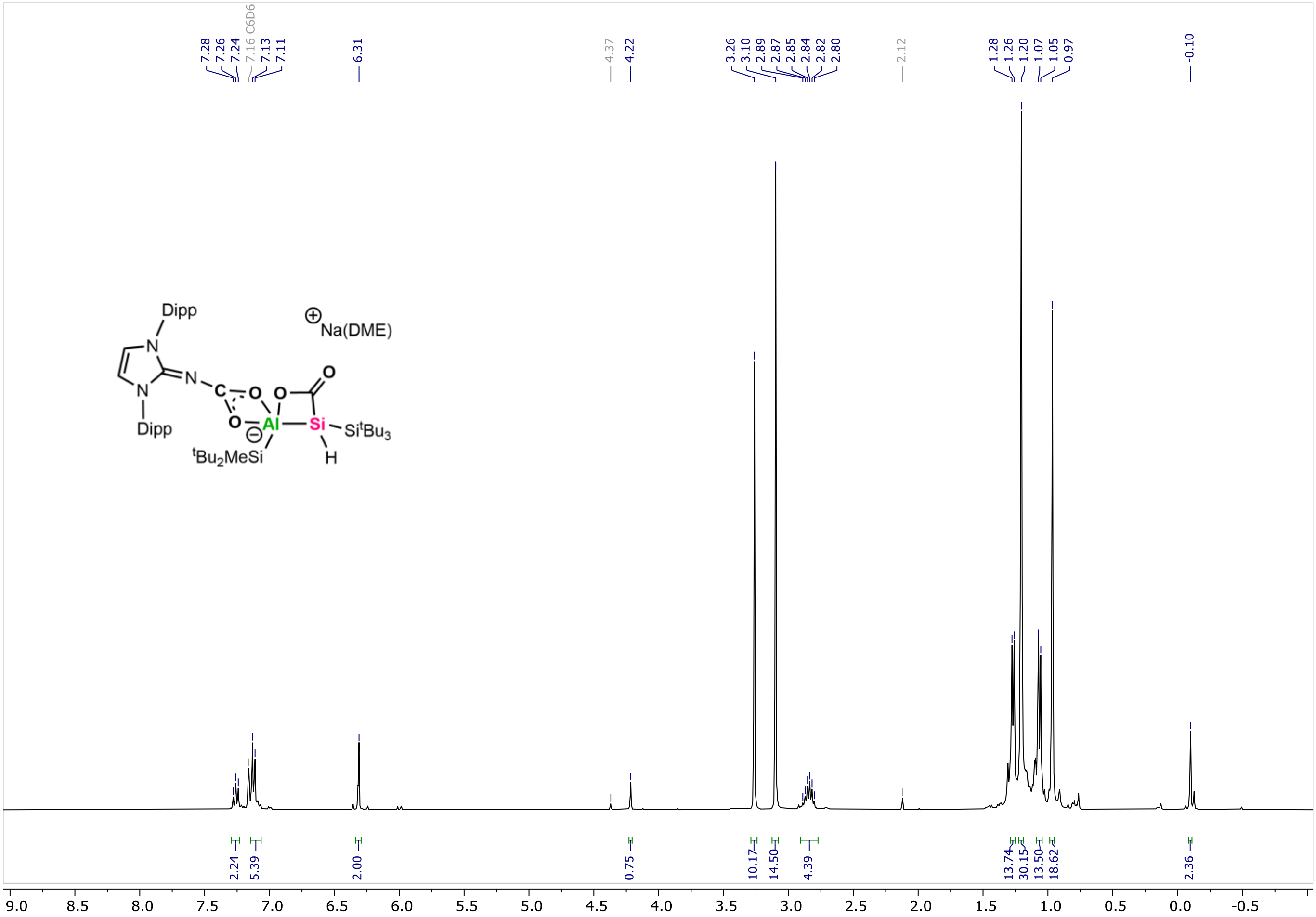

Figure S29: ^{1}H NMR spectrum (400.1 MHz, C_6D_6) of **4**.

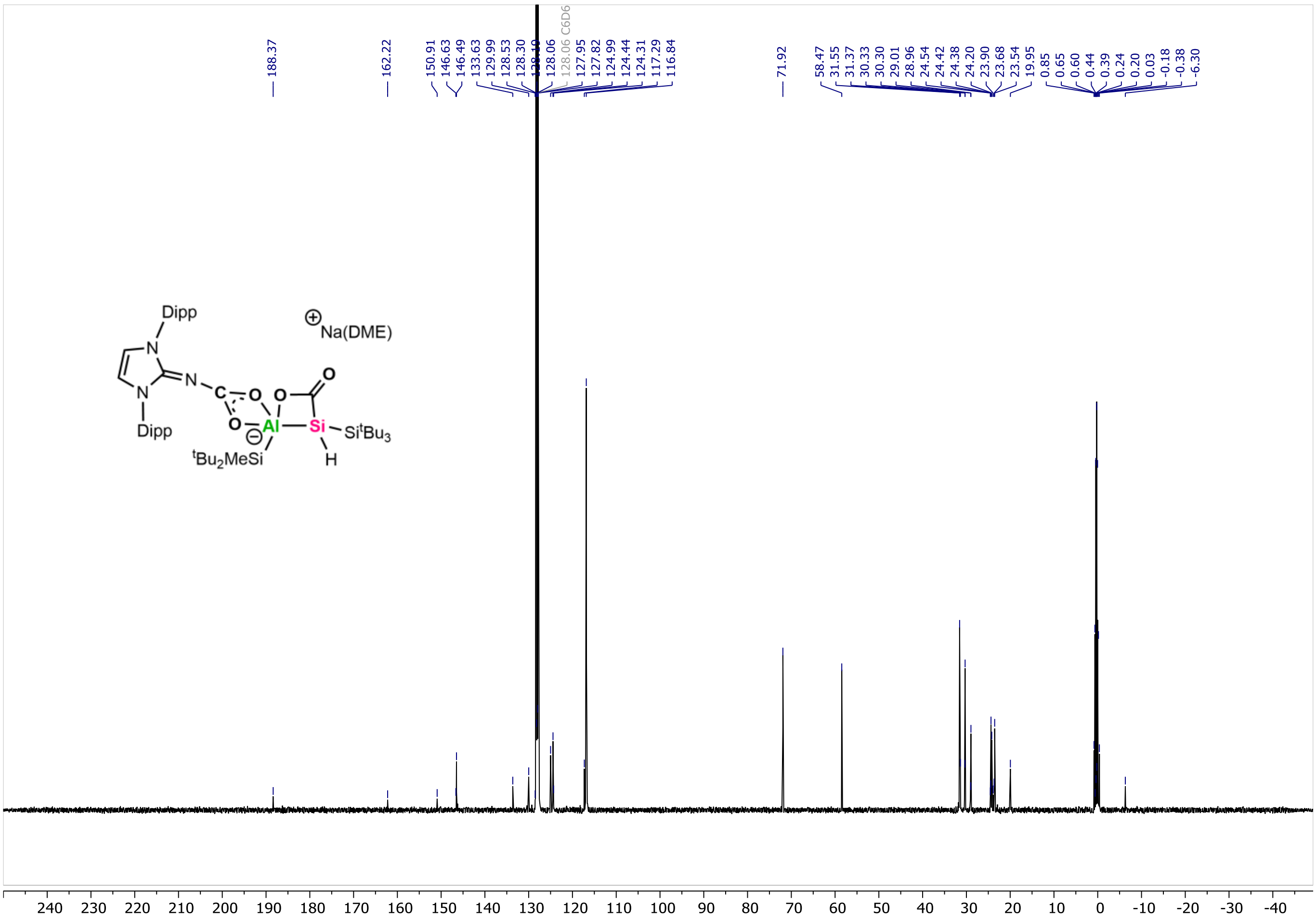

Figure S30: $^{13}C\ \{^1H\}$ NMR spectrum (100.6 MHz, C_6D_6) of **4**.

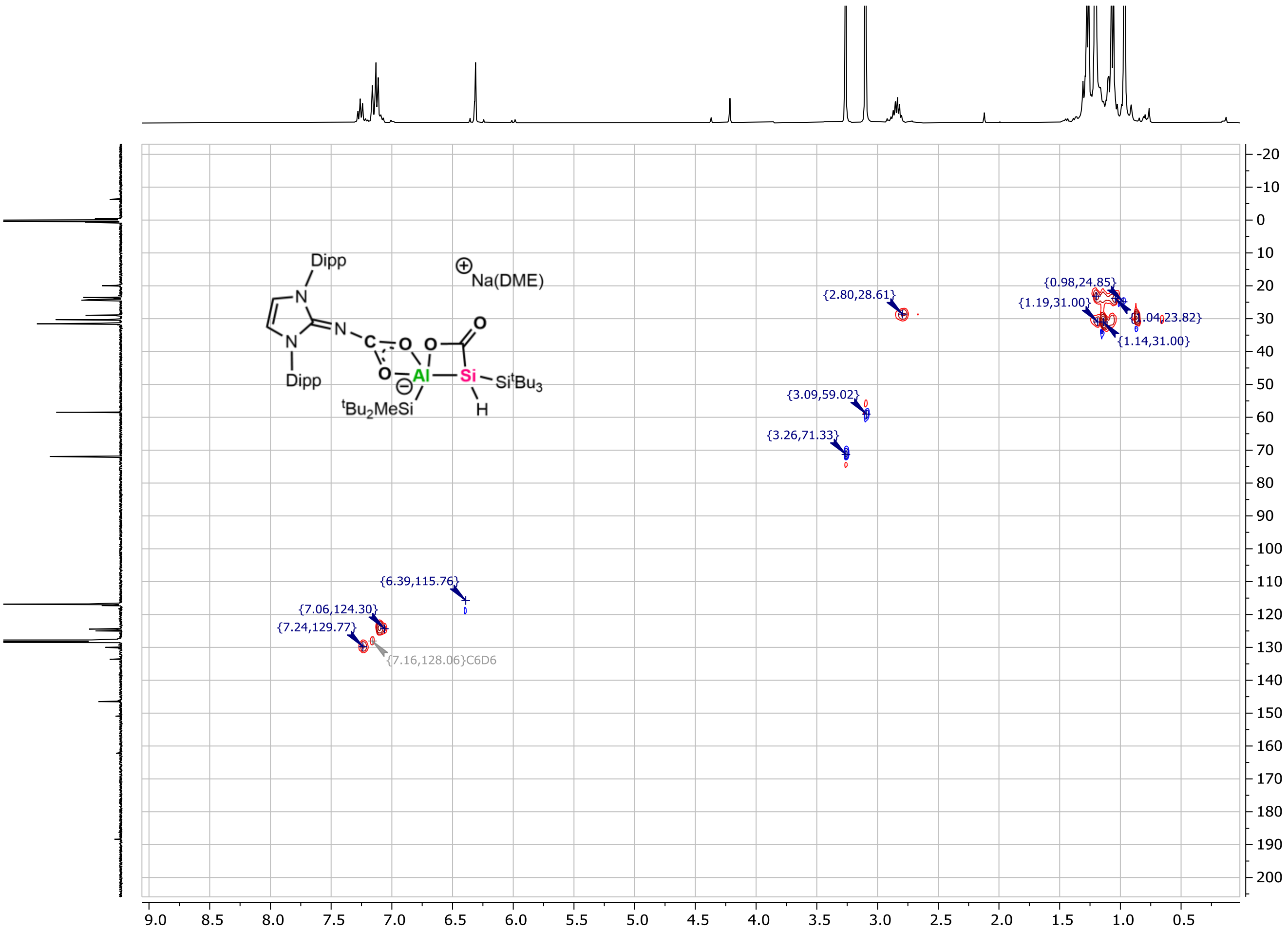

Figure S31: $^{1}H^{13}C$ HSQC NMR spectrum of **4** with $^{13}C\{^{1}H\}$ spectrum as vertical trace and ^{1}H spectrum as horizontal trace.

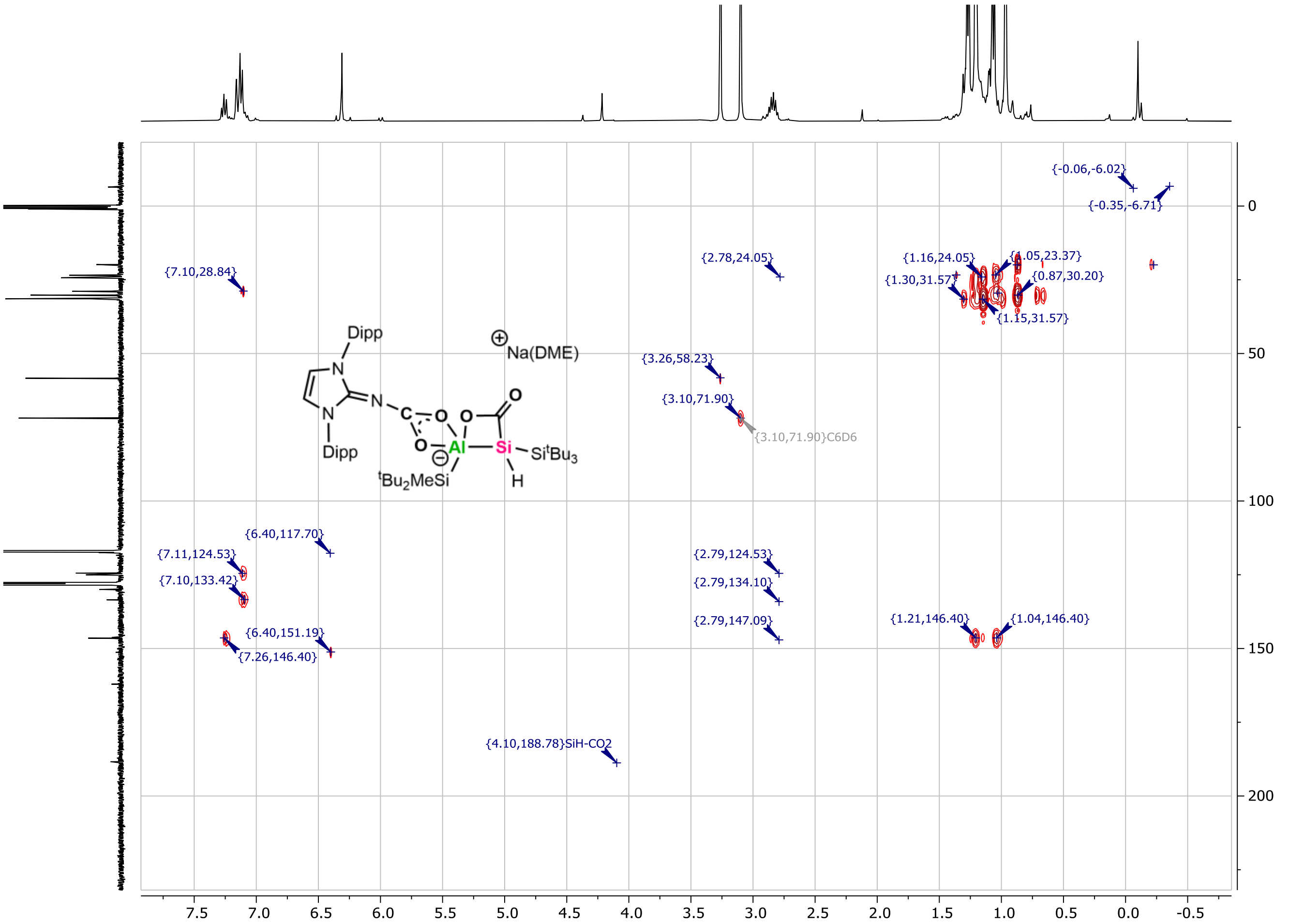

Figure S32: $^{1}H^{13}C$ HMBC NMR spectrum of **4** with $^{13}C\{^{1}H\}$ spectrum as vertical trace and ^{1}H spectrum as horizontal trace.

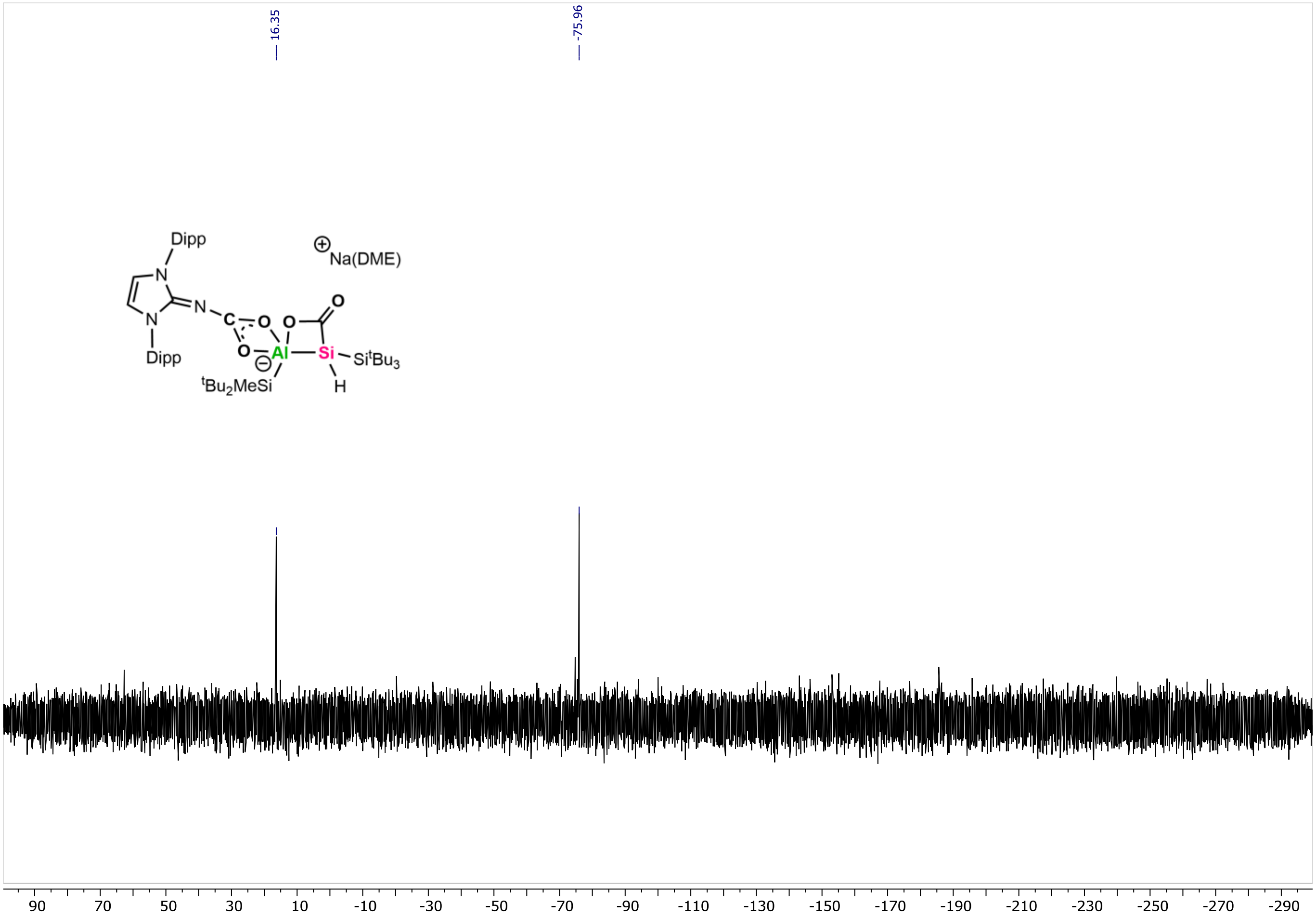

Figure S33: $^{29}Si\{^1H\}$ NMR spectrum (99.4 MHz, C_6D_6) of **4** including “zoom in” on the Si*H* signal. The satellite signals originate from the ***Si***H-***C***O_2 coupling (^1J = 70.8 Hz).

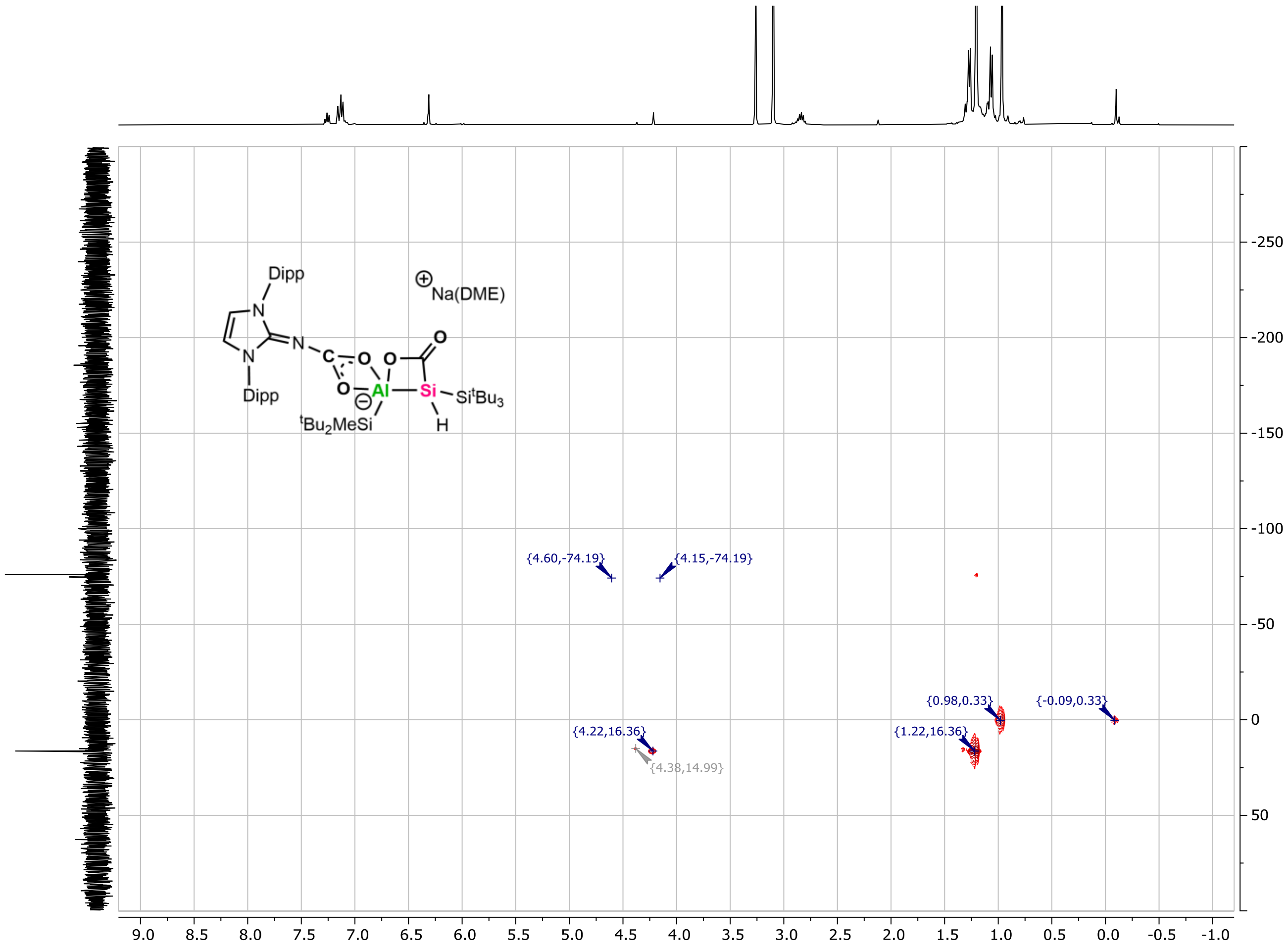

Figure S34: $^{1}H^{29}Si$ HMBC NMR spectrum of **4** with $^{29}Si\{^{1}H\}$ spectrum as vertical trace and ^{1}H spectrum as horizontal trace. The spectrum is referenced to the ***H***Si***Si***^{t}Bu3 cross-peak according to the ^{1}H and $^{29}Si\{^{1}H\}$ spectral data.

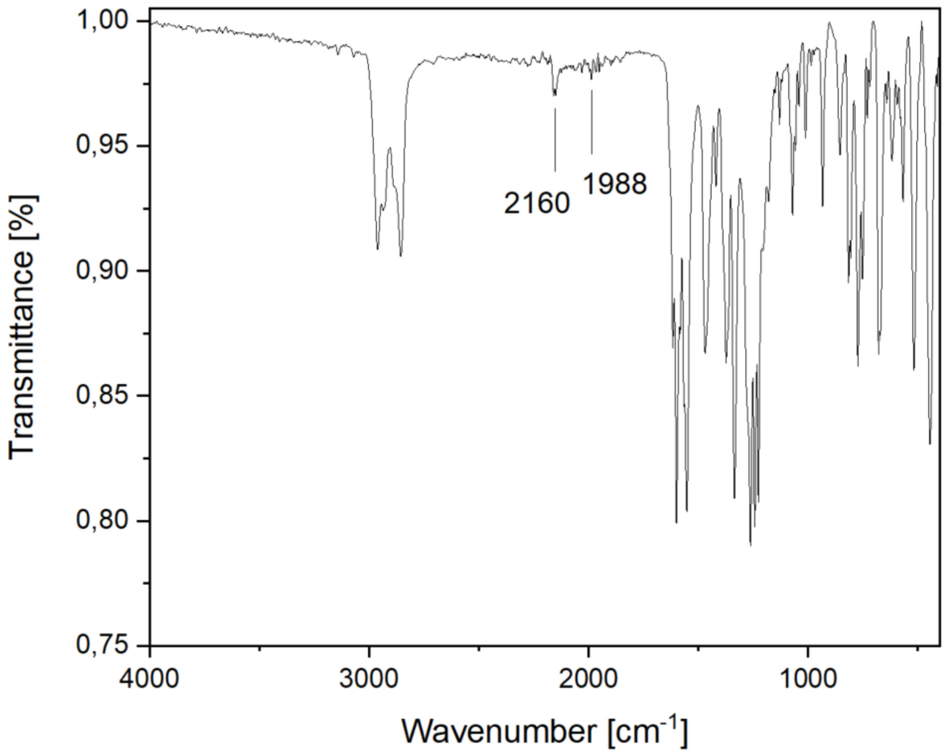

Figure S35: Solid-state FT-IR spectrum of **4**. The positions of the Si-CO_2 and Si-H band are marked.

Synthesis of ^{13}*C*-labeled Carbon Dioxide Cycloaddition Product (4^{13C})

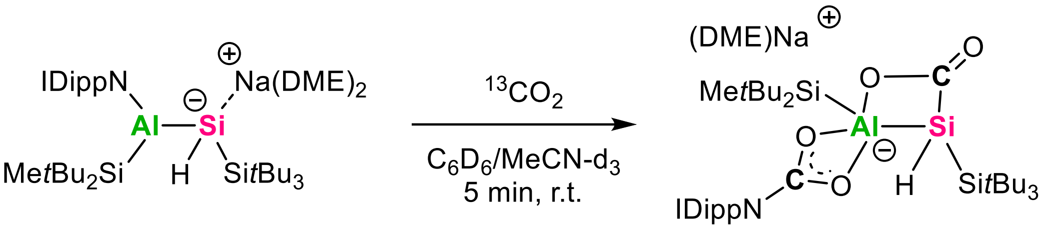

1a **4**^{13C}

For additional characteristic analytical data, the ^{13}*C*-labeled isotopomer **4**^{13C} was synthesized using the same procedure and $^{13}CO_2$ as described for method A. In a J-Young NMR tube, alumanyl silanide **1a** (11.0 mg, 10.8 µmol, 1 eq) was dissolved in C_6D_6 (0.3 mL). After freezing the reaction mixture and removing the argon atmosphere in dynamic vacuum, ^{13}CO2 (1.5 bar) was added to the frozen reaction mixture. After thawing and leaving the reaction mixture for 5 min at r.t., the reaction color changed from orange to colorless, with **4**^{13C} precipitating as fine white powder from the reaction solution. Subsequently, MeCN-d_3 (0.1 mL) was added to dissolve **4**^{13C}. NMR analysis shows the selective formation of **4**^{13C}.

FT-IR (neat, cm^{-1}): $\tilde{\nu}$ = 2361 (m) (Si-CO_2), 2078 (m) (Si-H).

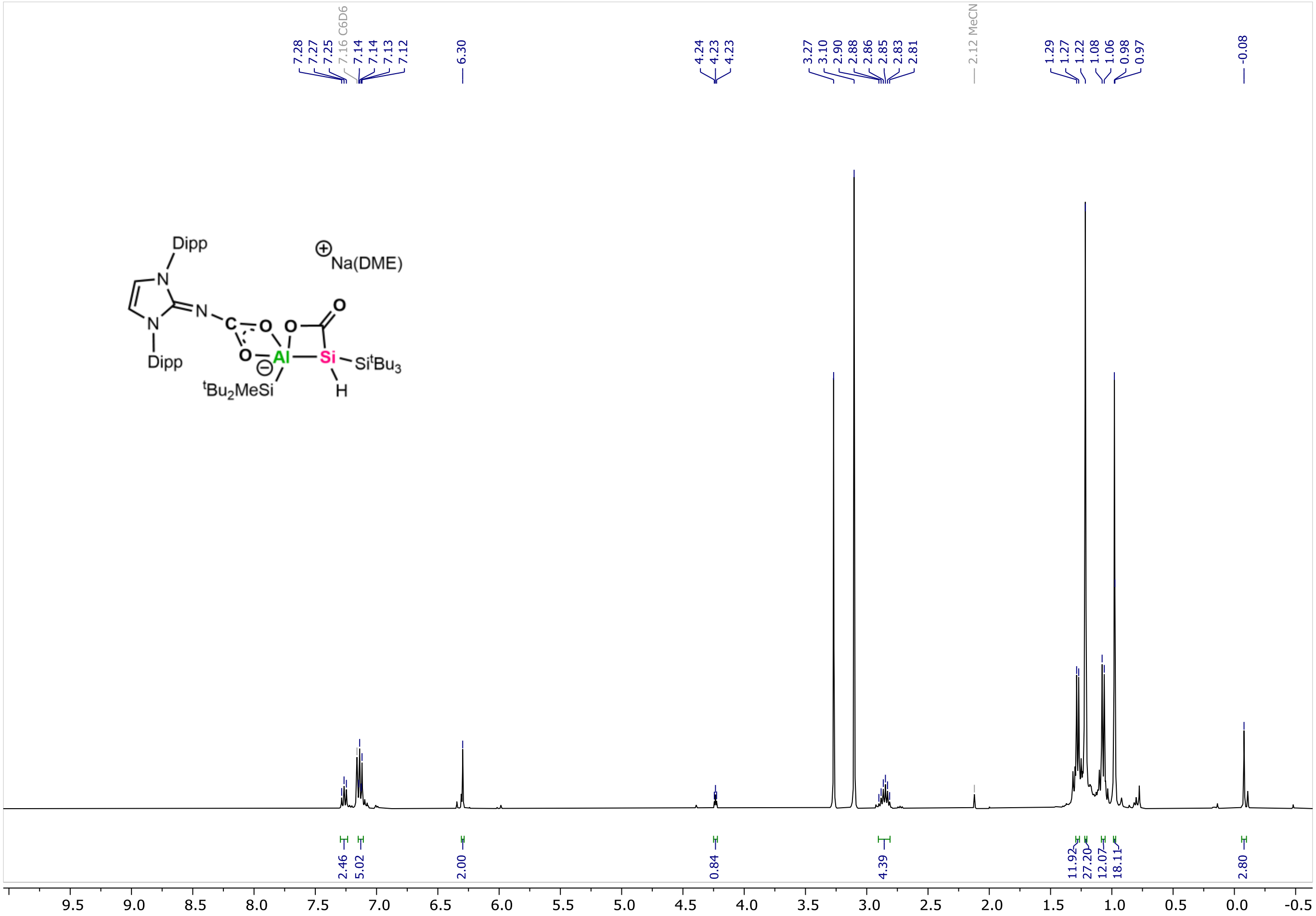

Figure S36: ^{1}H NMR spectrum (400.1 MHz, C_6D_6) of **4**^{13C}.

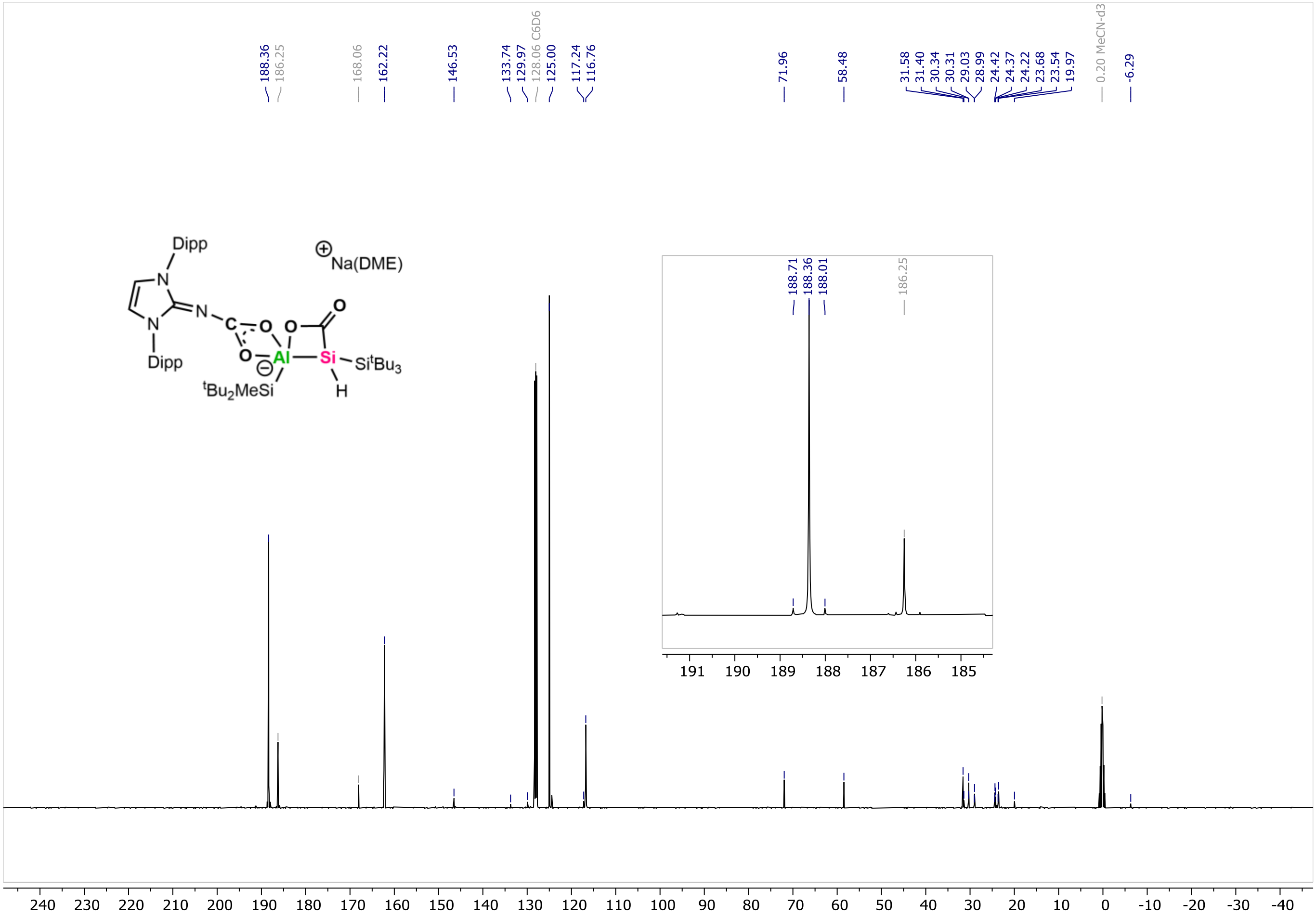

Figure S37: ^{13}C {^{1}H} NMR spectrum (100.6 MHz, C_6D_6) of **4**^{13C} including "zoom in" on the SiH-*C*O_2 signal. The satellite signals originate from the ***C***O_2-***Si***H coupling (^1J = 35.2 Hz).

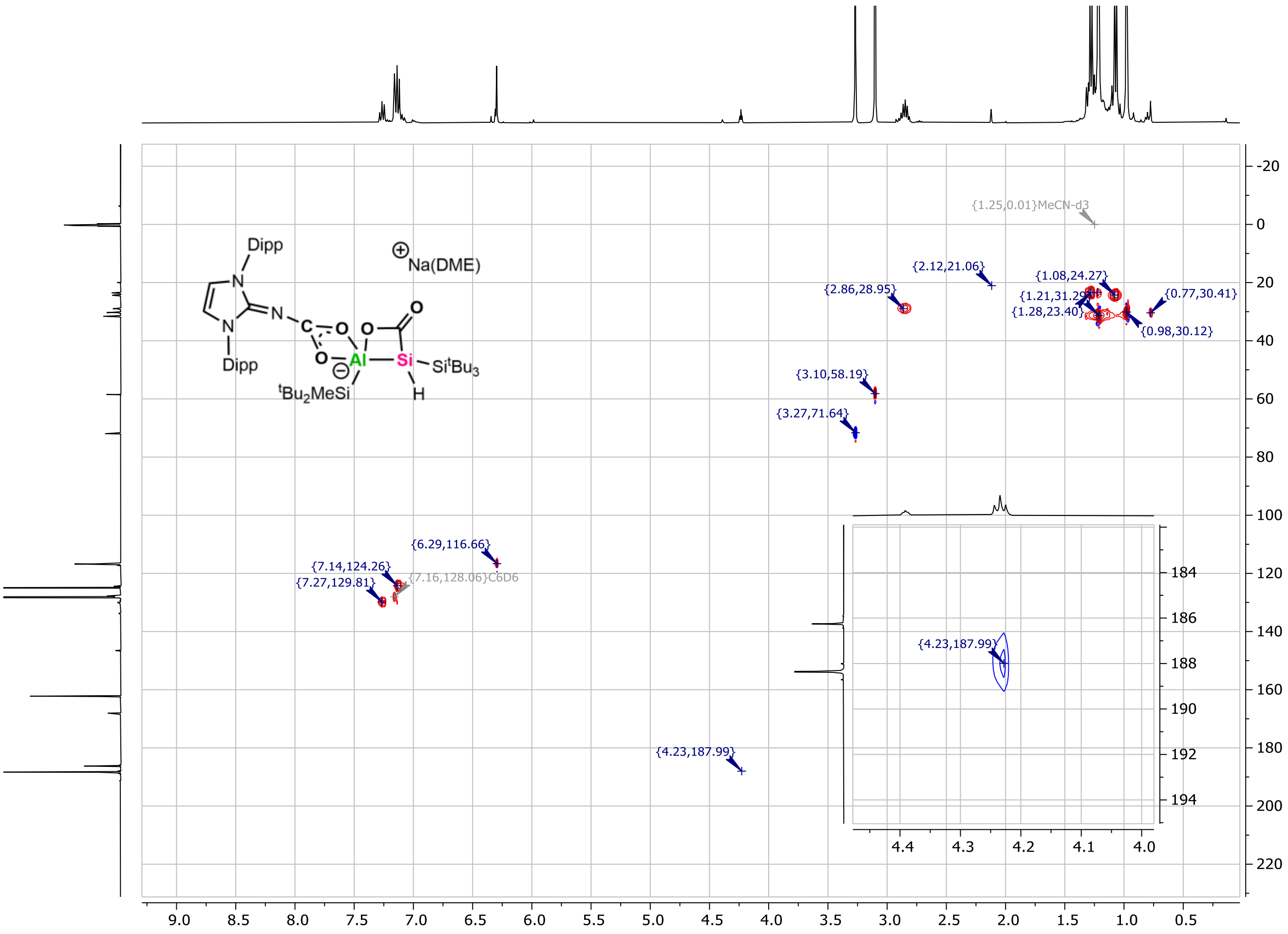

Figure S38: $^{1}H^{13}C$ HSQC NMR spectrum of **4**^{13C} with $^{13}C\{^{1}H\}$ spectrum as vertical trace and ^{1}H spectrum as horizontal trace including “zoom in” on Si***H***-***C***O_2 cross peak.

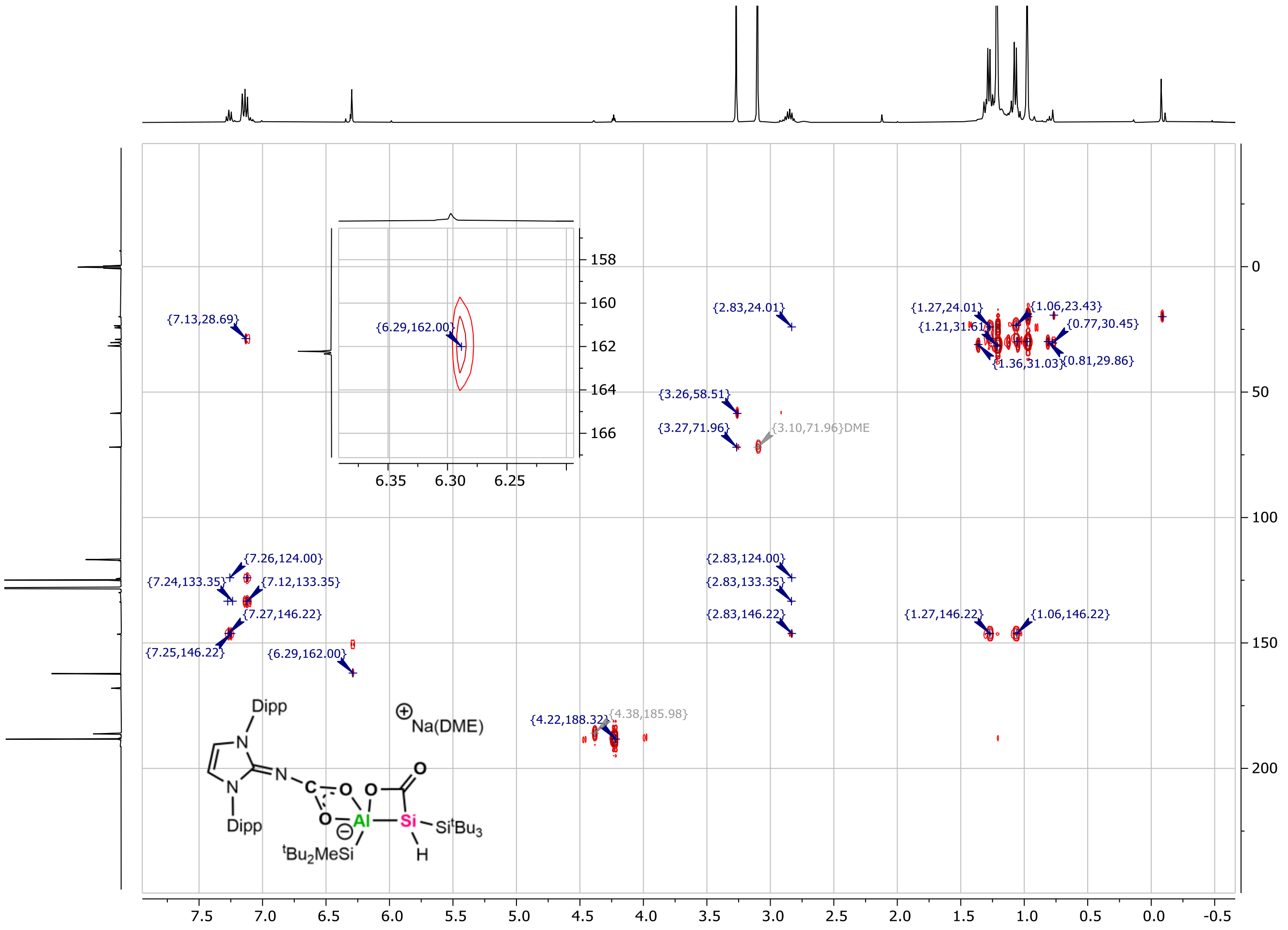

Figure S39: $^{1}H^{13}C$ HMBC NMR spectrum of **4**^{13C} with $^{13}C\{^{1}H\}$ spectrum as vertical trace and ^{1}H spectrum as horizontal trace including "zoom in" on NC***H***-***C***O$_2$ cross peak.

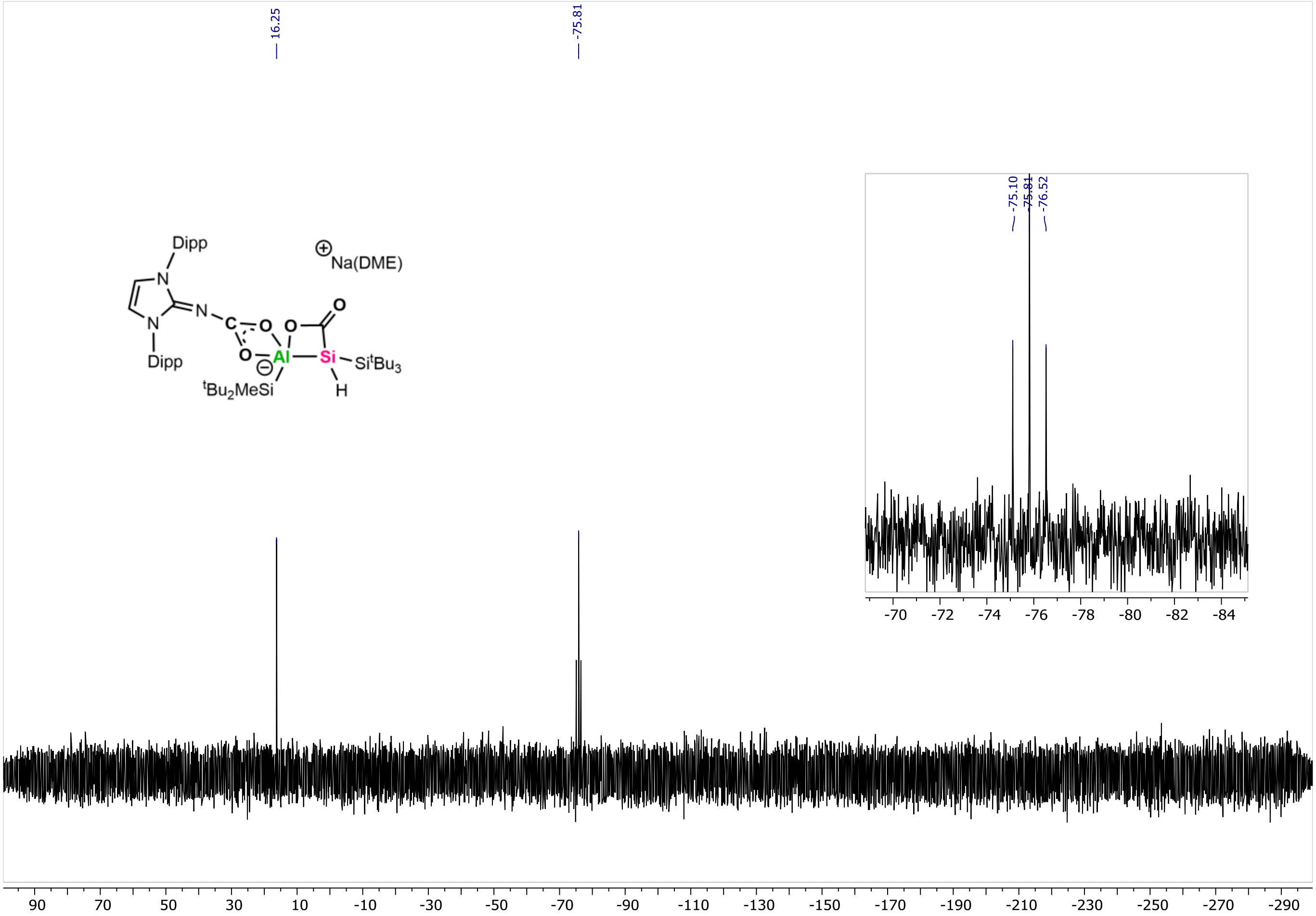

Figure S40: $^{29}Si\{^{1}H\}$ NMR spectrum (99.4 MHz, C_6D_6) of $\mathbf{4}^{13C}$ including “zoom in” on the Si*H* signal. The satellite signals originate from the ***Si***H-***C***O_2 coupling (^{1}J = 70.8 Hz).

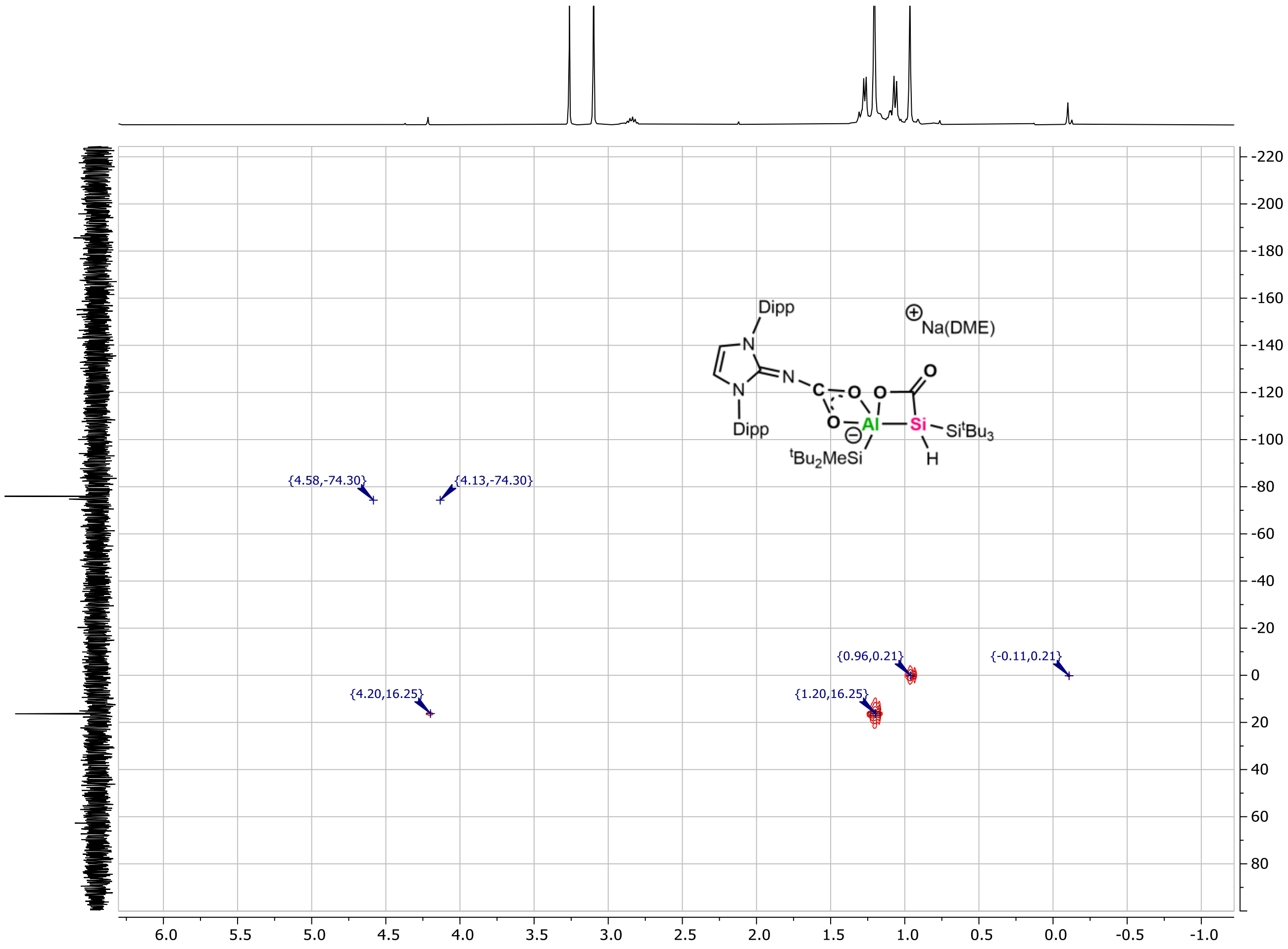

Figure S41: $^{1}H^{29}Si$ HMBC NMR spectrum of **4**^{13C} with $^{29}Si\{^{1}H\}$ spectrum as vertical trace and ^{1}H spectrum as horizontal trace. The spectrum is referenced to the ***H***Si***Si***^{t}Bu$_3$ cross-peak according to the ^{1}H and $^{29}Si\{^{1}H\}$ spectral data.

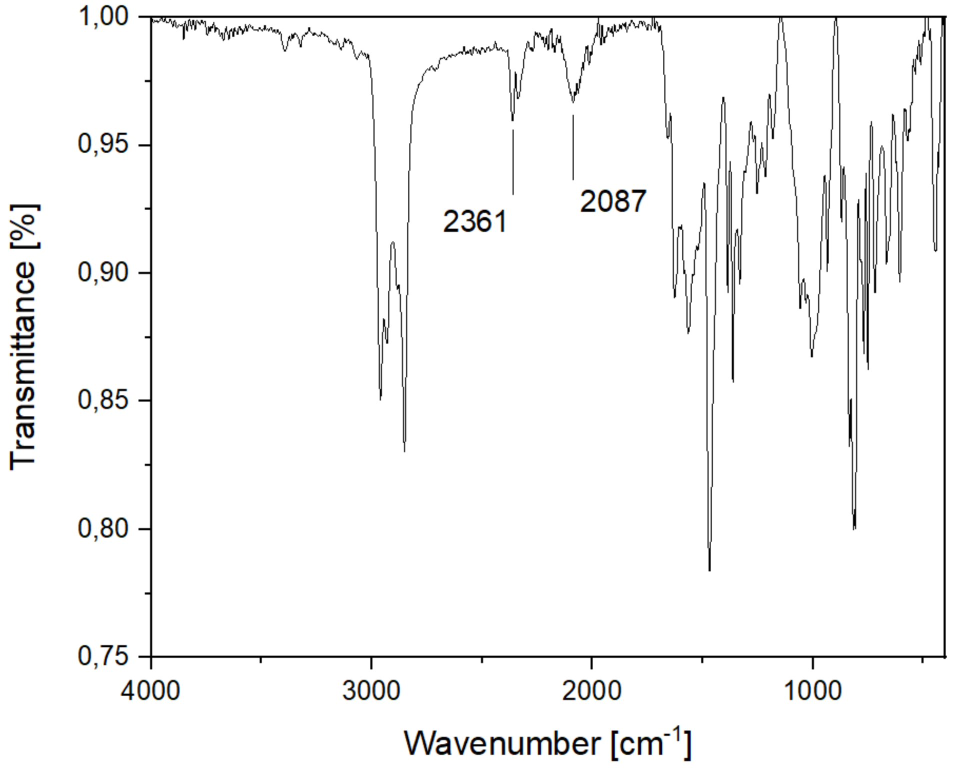

Figure S42: Solid-state FT-IR spectrum of **4**^{13C}. The positions of the Si-CO_2 and Si-H band are marked.

Synthesis of Mesitylaldehyde Cycloaddition Product (5)

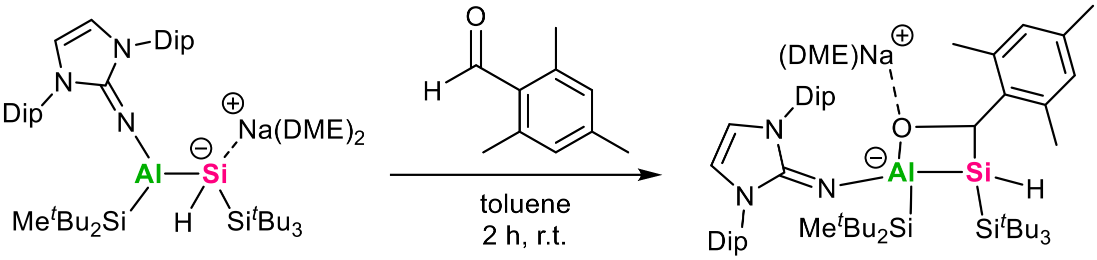

1a **5**

A solution of mesitylaldehyde (14.6 µL, 98.2 µmol, 1 eq) in toluene (1.5 mL) was added dropwise to an orange solution of alumanyl silanide **1a** (100 mg, 98.2 µmol, 1 eq) in toluene (6 mL) with stirring. The reaction mixture was stirred at r.t. for 2 h, during which the color changed from orange to yellow. After that, all volatiles were removed in vacuum. The solid was subsequently washed with n-pentane (3 mL) and dried in vacuum. The product was obtained in form of a white powder (84.6 mg, **74%**). Crystals suitable for SC-XRD analysis were obtained by slow crystallization in a concentrated benzene solution under inert atmosphere at r.t..

^{1}H NMR (400.1 MHz, C_6D_6): δ = 6.99 (m, 6H, DipH), 6.69 (d, ^{4}J = 2 Hz, 1H, MesH), 6.78 (d, ^{4}J = 2 Hz, 1H, MesH), 6.09 (s, 2H, NC*H*), 5.65 (d, ^{3}J = 8 Hz, 1H, Si-C*H*-O), 4.44 (d, ^{3}J = 8 Hz, 1H, Si*H*), 3.58 (sept, ^{3}J = 7 Hz, 2H, C*H*-^{i}Pr), 3.29 (sept, ^{3}J = 7 Hz, 2H, C*H*-^{i}Pr), 2.89 (s, 6H, CH_3-DME), 2.83 (s, 4H, CH_2-DME), 2.60 (s, 3H, Mes-CH_3), 2.53 (s, 3H, Mes-CH_3), 2.15 (s, 3H, Mes-CH_3), 1.47 (d, ^{3}J = 7 Hz, 6H, CH_3-^{i}Pr), 1.45 (d, ^{3}J = 7 Hz, 6H, CH_3-^{i}Pr), 1.32 (s, 9H, Si^{t}Bu$_2$), 1.30 (s, 27H, Si^{t}Bu$_3$), 1.23 (s, 9H, Si^{t}Bu$_2$), 1.10 (d, ^{3}J = 7 Hz, 6H, CH_3-^{i}Pr), 1.07 (d, ^{3}J = 7 Hz, 6H, CH_3-^{i}Pr), 0.27 (s, 3H, SiCH_3). **^{13}C{^{1}H} NMR** (100.6 MHz, C_6D_6): δ = 148.5 (NHI-C_{aryl}), 148.2 (NHI-C_{aryl}), 142.7 (Mes-C_{aryl}), 137.3 (Mes-C_{aryl}), 136.0 (NHI-C_{aryl}), 135.6 (NHI-C_{aryl}), 133.7 (Mes-C_{aryl}), 130.2 (Mes-CH), 129.7 (Mes-CH), 129.2 (Mes-C_{aryl}), 124.8 (NHI-C_{aryl}), 124.0 (NHI-C_{aryl}), 114.3 (N*C*H), 71.0 (DME-*C*H_2), 65.8 (Si-*C*H-O), 58.5 (DME-*C*H_3), 32.4 (Si^{t}Bu$_3$), 31.5 (Si^{t}Bu$_2$), 31.3 (Si^{t}Bu$_2$), 29.0 (*C*H-^{i}Pr), 28.4 (*C*H-^{i}Pr), 26.7 (*C*H_3-^{i}Pr), 25.8 (*C*H_3-^{i}Pr), 24.0 (NHI), 23.5 (Mes-*C*H_3), 22.8 (*C*H_3-^{i}Pr), 22.5 (Mes-*C*H_3), 21.3 (Mes-*C*H_3), 20.9 (Mes-*C*H_3), 20.4 (Mes-*C*H_3), -4.6 (Si*C*H_3). **^{29}Si{^{1}H} NMR** (99.4 MHz, C_6D_6): δ = 19.9 (*Si*^{t}Bu$_3$), -0.5 (*Si*^{t}Bu$_2$)[a], -59.9 (*Si*H).

FT-IR (neat, cm^{-1}): $\tilde{\nu}$ = 2091 (s) (Si-H).

Melting point: 141.0°C

Notes: **a**: The assessment of the Si^{t}Bu$_2$ signal was verified by ^{1}H^{29}Si HMBC experiment.

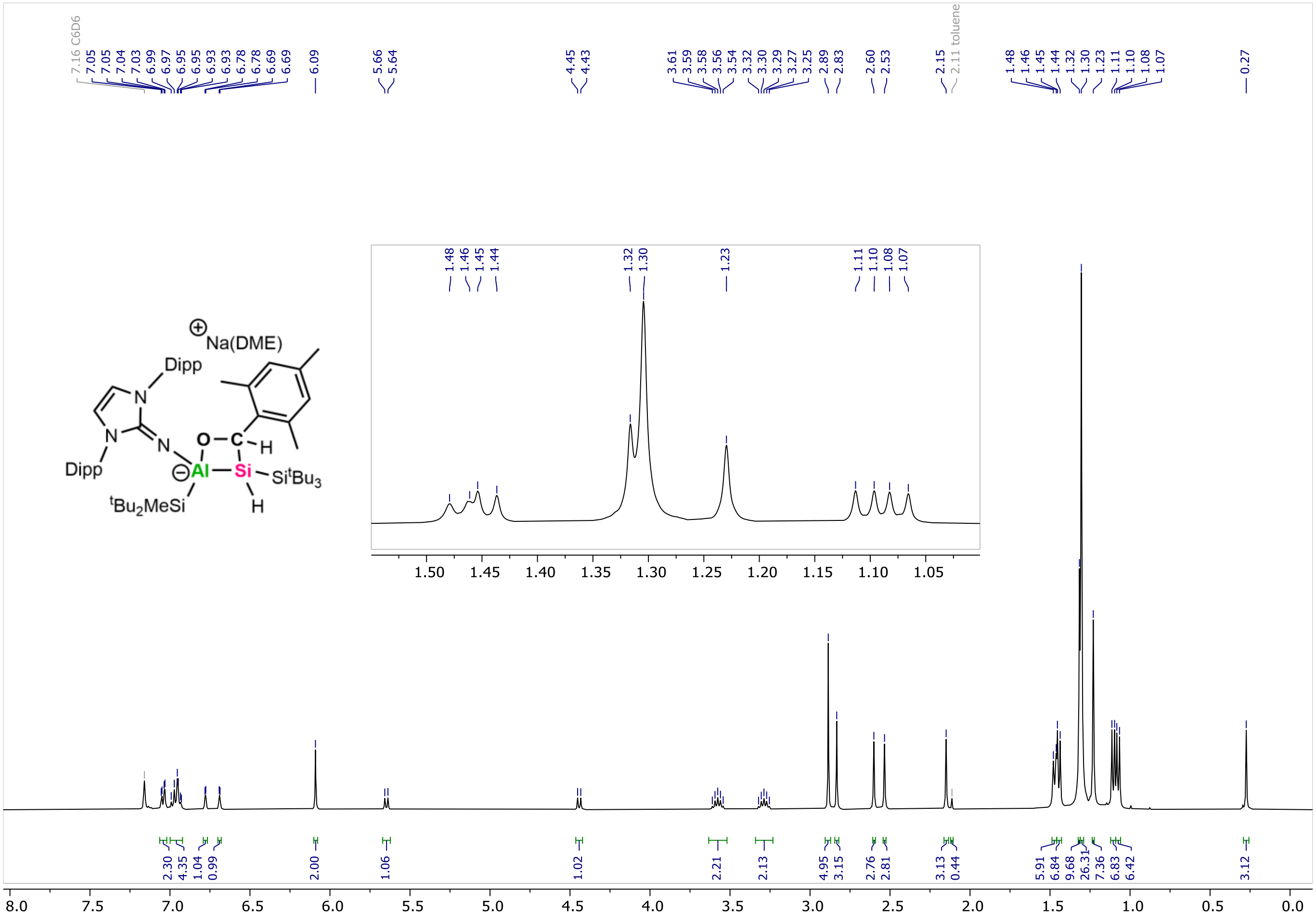

Figure S43: ^1H NMR spectrum (400.1 MHz, C_6D_6) of **5** including “zoom in” on the aliphatic region.

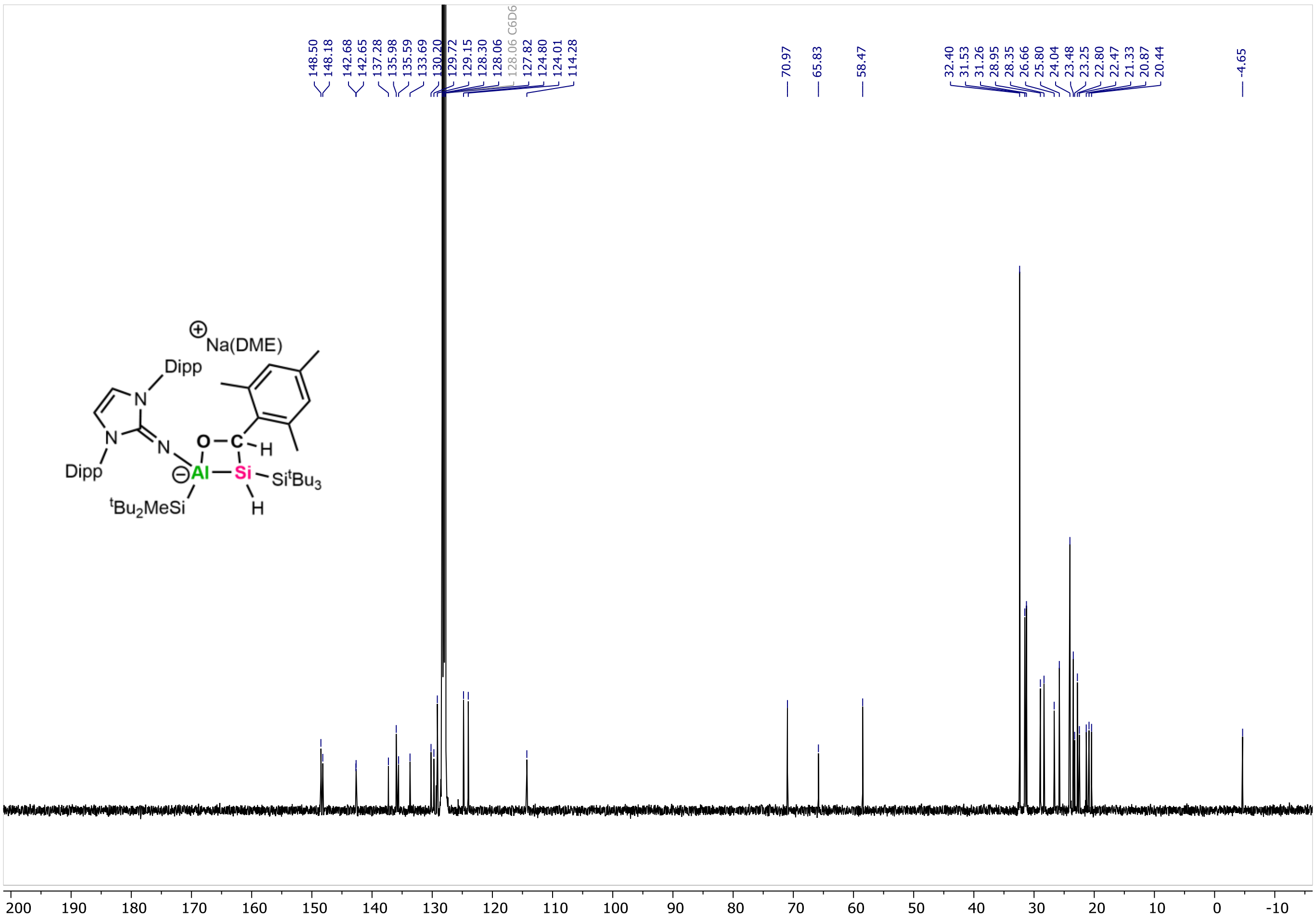

Figure S44: ^{13}C {^{1}H} NMR spectrum (100.6 MHz, C_6D_6) of **5**.

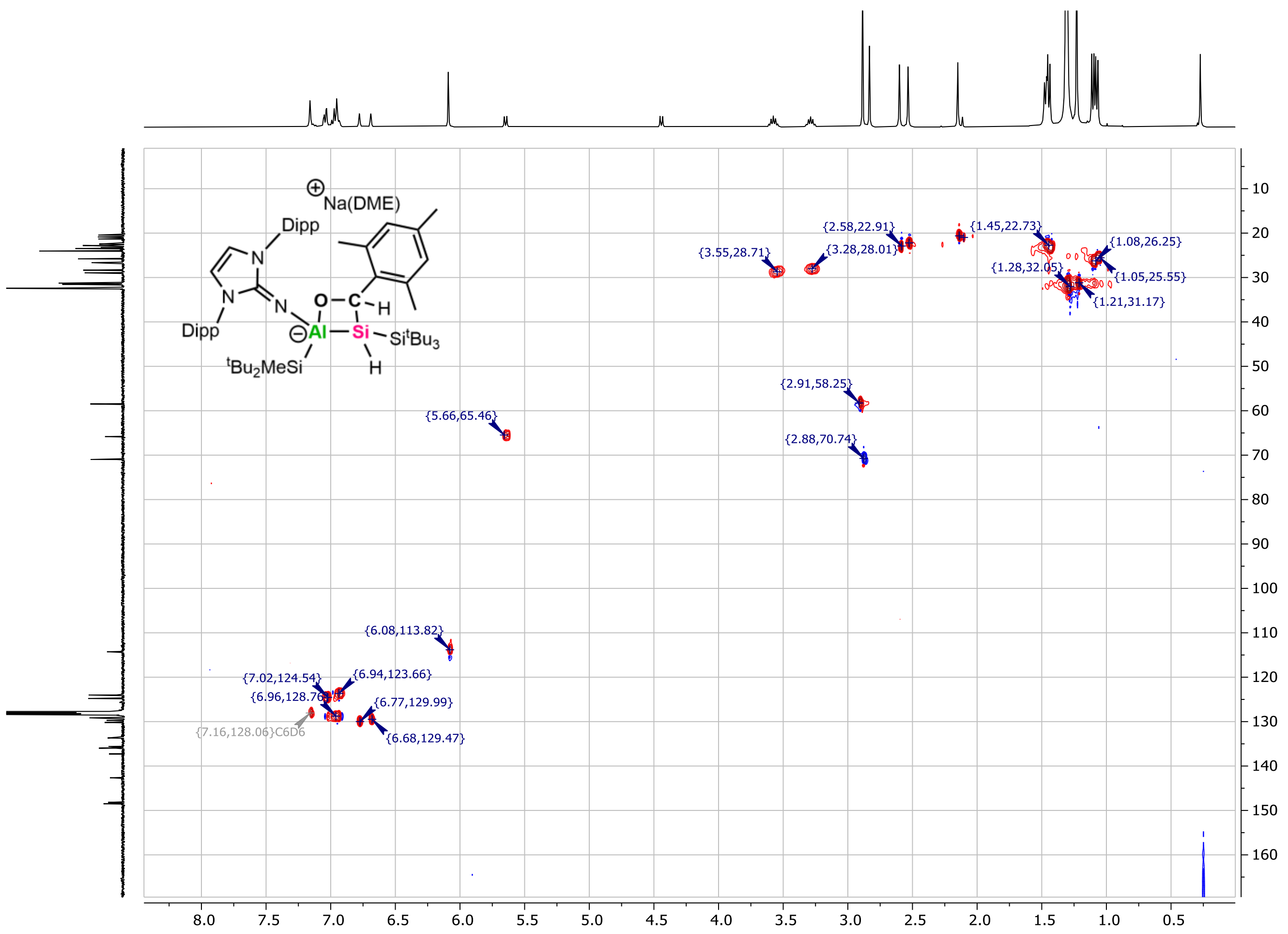

Figure S45: $^{1}H^{13}C$ HSQC NMR spectrum of **5** with $^{13}C\{^{1}H\}$ spectrum as vertical trace and ^{1}H spectrum as horizontal trace.

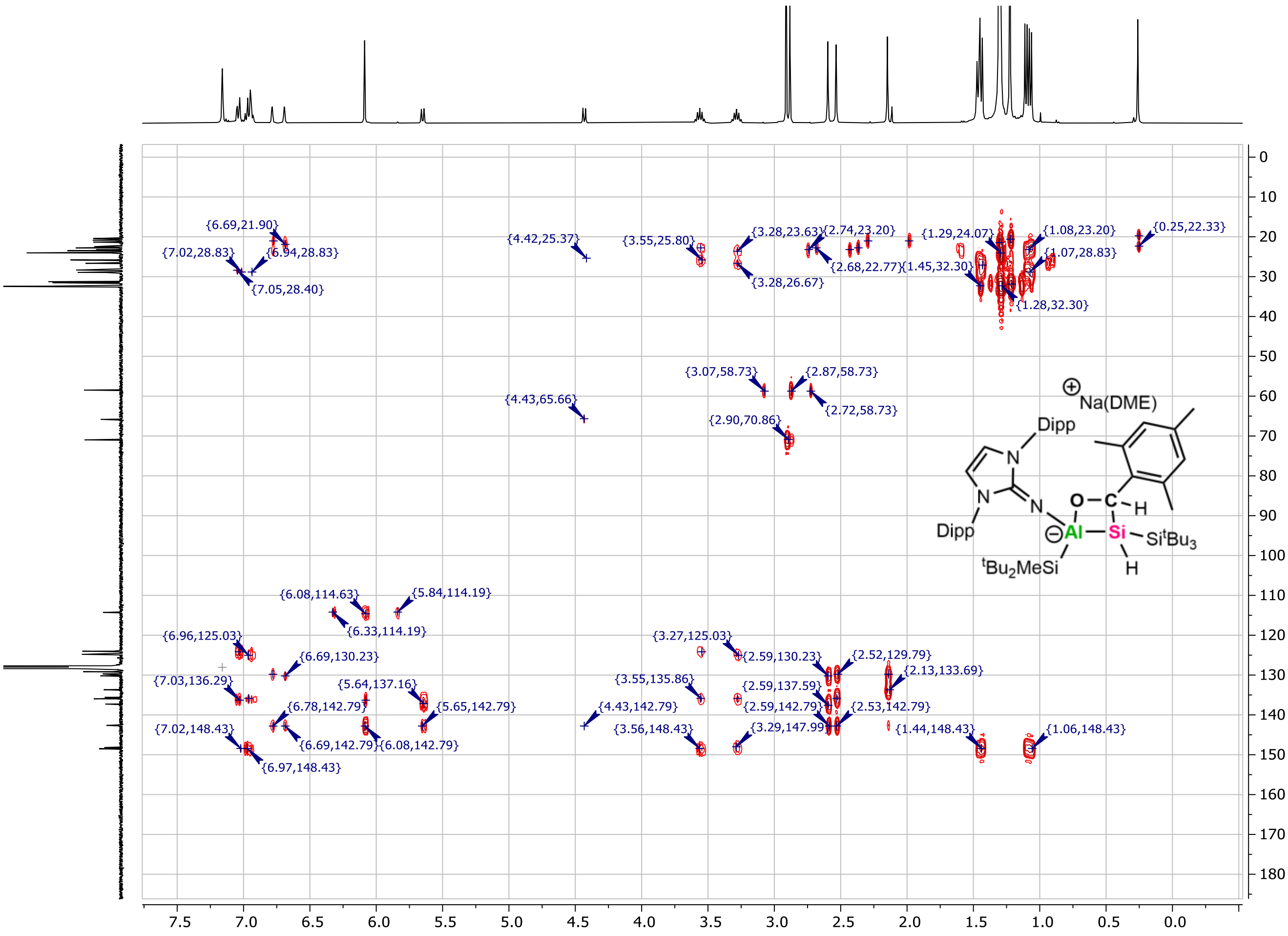

Figure S46: $^1H^{13}C$ HMBC NMR spectrum of **5** with $^{13}C\{^1H\}$ spectrum as vertical trace and ^1H spectrum as horizontal trace.

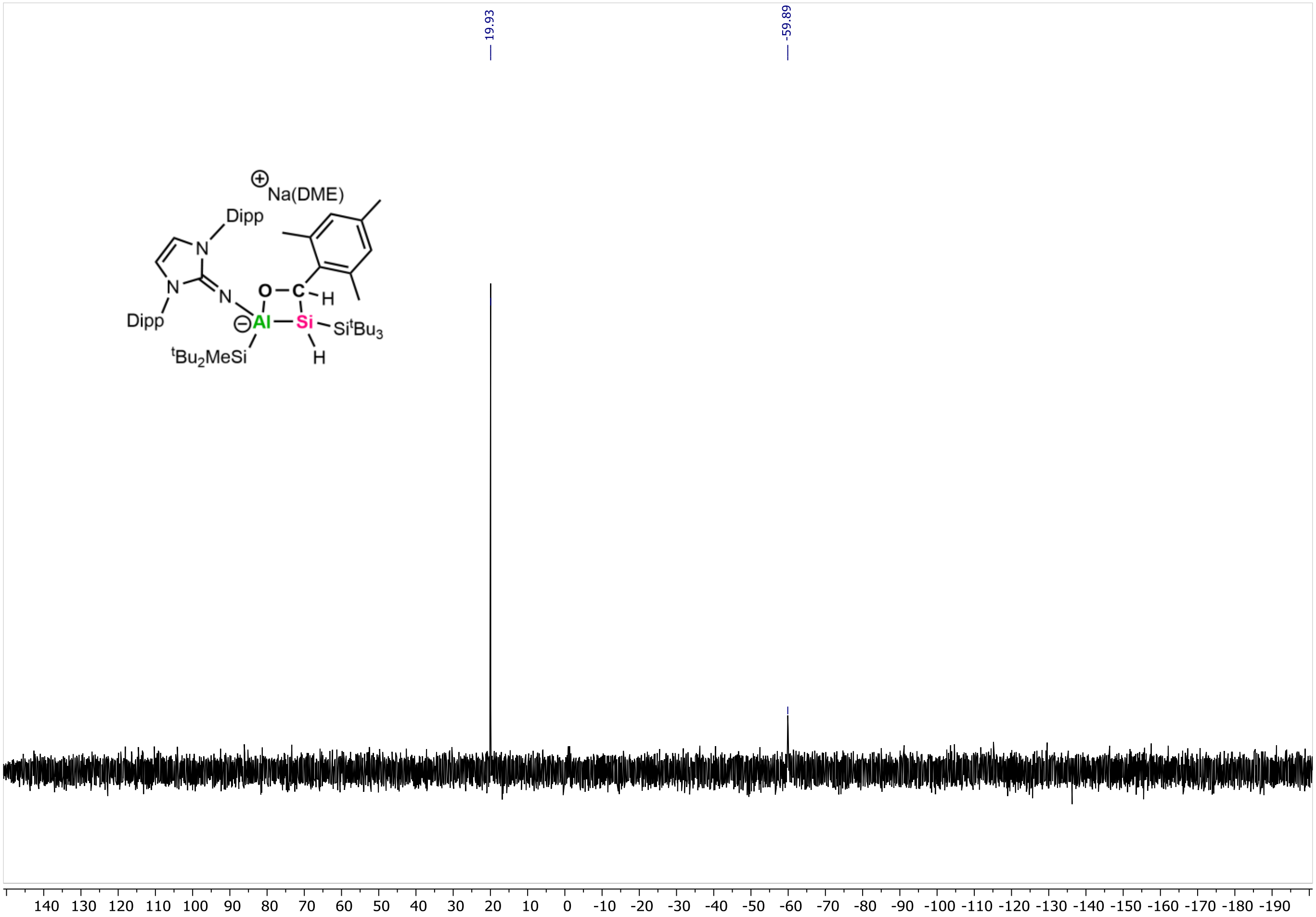

Figure S47: $^{29}Si\{^1H\}$ NMR spectrum (99.4 MHz, C_6D_6) of **5**.

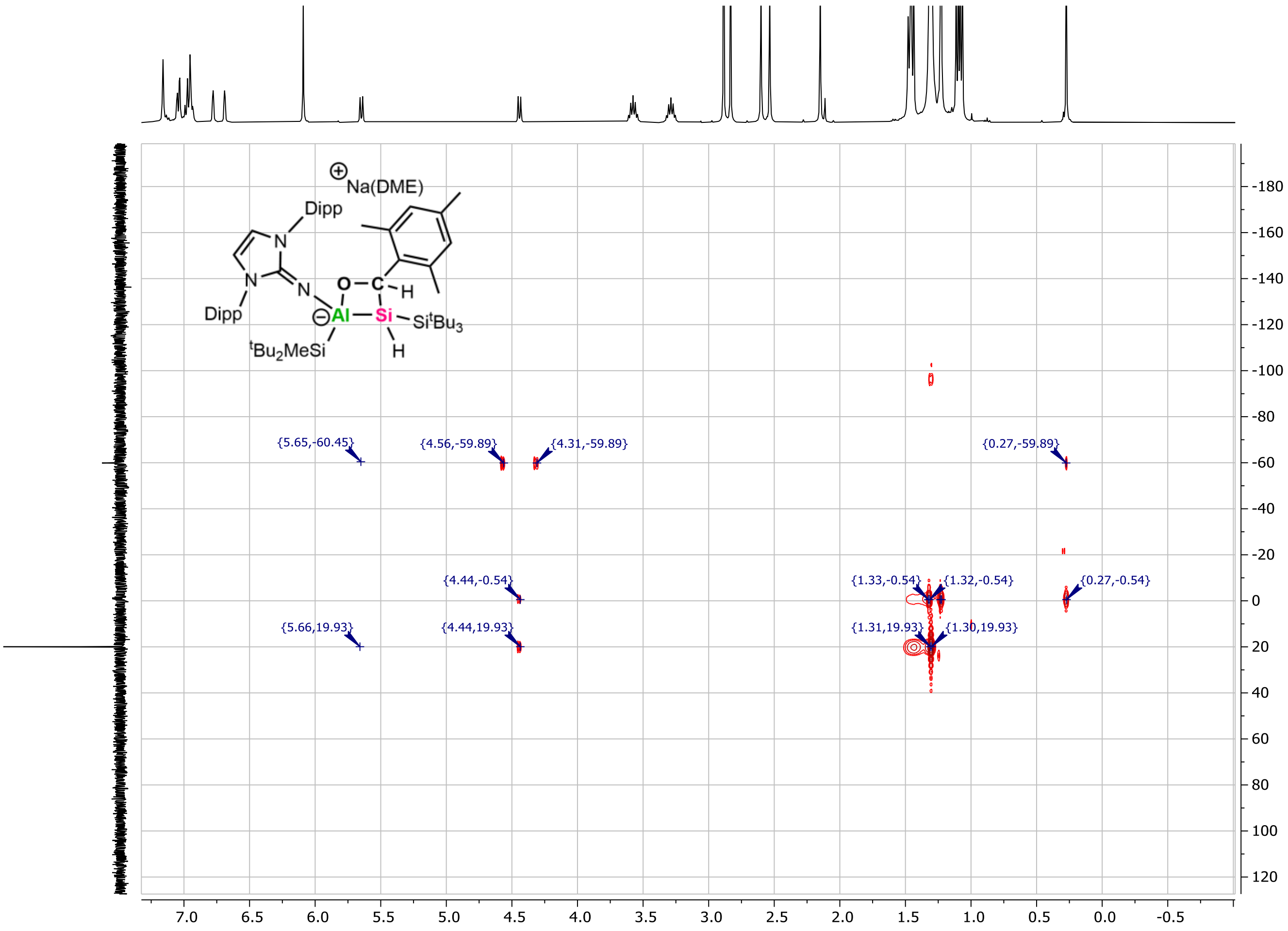

Figure S48: ^{1}H^{29}Si HMBC NMR spectrum of **5** with ^{29}Si{^{1}H} spectrum as vertical trace and ^{1}H spectrum as horizontal trace. The spectrum is referenced to the ***H***Si***Si***^{t}Bu$_3$ cross-peak according to the ^{1}H and ^{29}Si{^{1}H} spectral data.

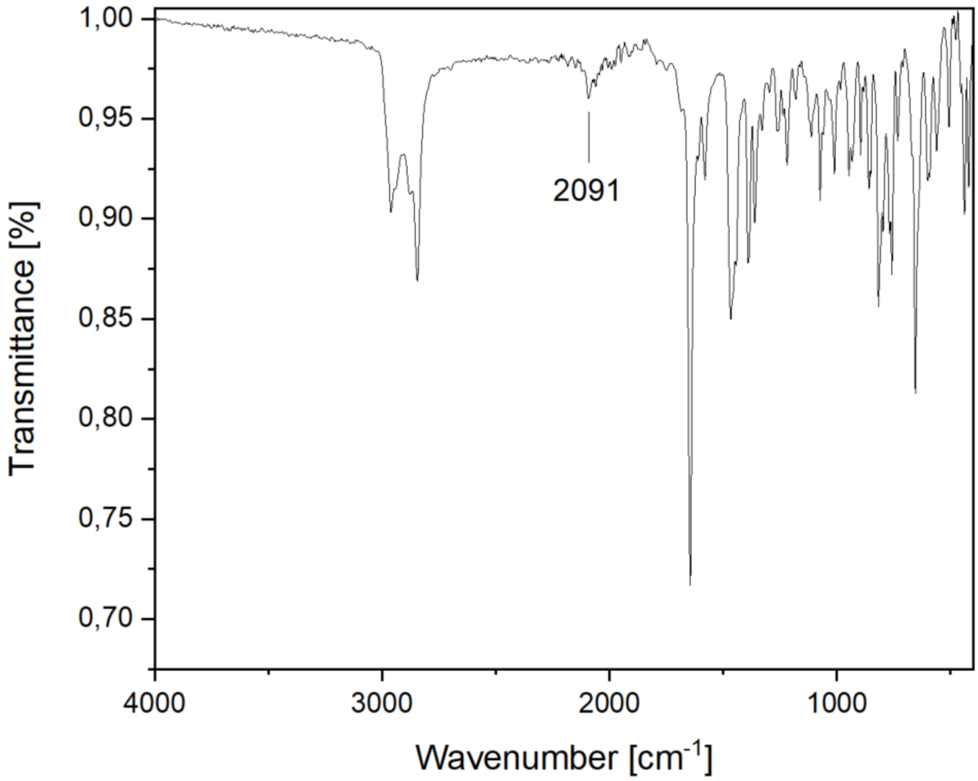

Figure S49: Solid-state FT-IR spectrum of **5**. The position of the Si-H band is marked.

Synthesis of Cyclic Silacarbene (6)

Method A (NMR scale):

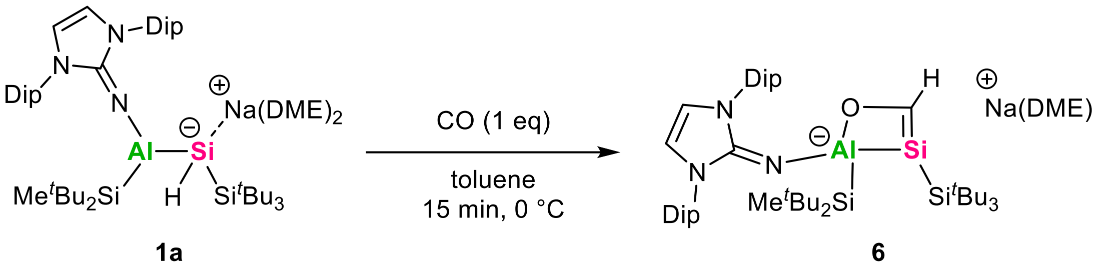

Alumanyl silanide **1a** (29.8 mg, 32.3 μmol, 1 eq) was dissolved in toluene (2 mL) in a J-Young NMR tube with a total volume of 2.6 mL. Subsequently, CO (1.5 bar) was added at 0 °C without degassing, after which the color changed from an initially orange to orange-yellow within 15 min. After leaving the reaction solution at r.t. for another 12 h, the solvent was removed in vacuum and the crude product was washed with *n*-pentane (1 mL). After drying in vacuum, **6** was obtained as orange powder (19.7 mg, **64%**).

Method B (big scale):

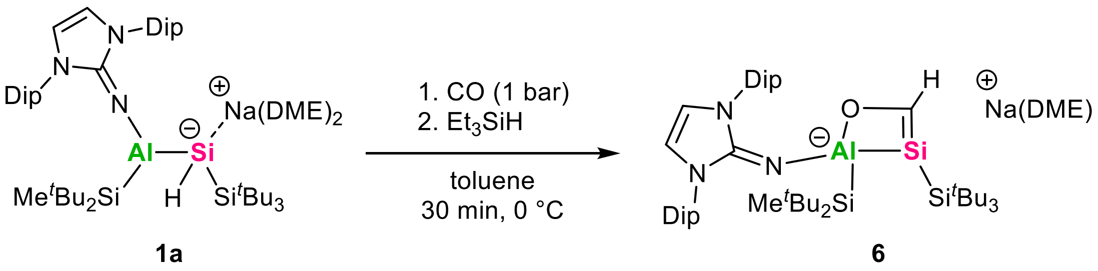

A solution of alumanyl silanide **1a** (200 mg, 215 μmol, 1 eq) in toluene (10 mL) was frozen with subsequent removal of the argon atmosphere in dynamic vacuum. After pressurizing the reaction mixture with CO (1 bar), the color changed from an initially orange to green over the course of 30 min at 0 °C. The reaction solution was frozen and the CO atmosphere was removed in dynamic vacuum. A solution of triethylsilane (34.4 μL, 215 μmol, 1 eq) in toluene (1 mL) was added dropwise over 5 min at 0 °C.[a] After stirring at 0 °C for 30 min, the reaction was allowed to warm to r.t. and stirred for another 1 h, during which the reaction color changed to orange-yellow. After removing the solvent in vacuum, the crude product was washed with *n*-pentane (1 mL) and dried in vacuum. The product was obtained as orange powder (129 mg, **63%**).

Notes: **a**: The addition of triethylsilane presumably quenches reactive intermediates and increases both purity and yield of **6**.

^{1}**H NMR** (400.1 MHz, C_6D_6): *δ* = 8.72 (s, 1H, C*H*O), 7.13 (m, 6H, DipH), 5.98 (s, 2H, NC*H*), 3.64 (bs, 2H, C*H*-^{i}Pr), 3.27 (bs, 2H, C*H*-^{i}Pr), 2.99 (s, 6H, C*H*$_3$-DME), 2.85 (s, 4H, C*H*$_2$-DME), 1.51 (s, 27H, Si^{t}Bu$_3$), 1.40 (m, 12H, C*H*$_3$-^{i}Pr), 1.29 (s, 9H, Si^{t}Bu$_2$), 1.23 (m, 12H, C*H*$_3$-^{i}Pr), 1.19 (s, 9H, Si^{t}Bu$_2$), 0.12 (s, 3H, SiCH$_3$). ^{13}**C{^{1}H NMR** (100.6 MHz, C_6D_6): *δ* = 199.8 (*C*HO), 149.4 (NHI-*C*$_{aryl}$), 148.7 (NHI-*C*$_{aryl}$), 139.8 (NHI-*C*$_{aryl}$), 137.9 (NHI-*C*$_{aryl}$), 136.8 (NHI-*C*$_{aryl}$), 125.7 (NHI-*C*$_{aryl}$), 124.2 (NHI-*C*$_{aryl}$), 123.7 (NHI-*C*$_{aryl}$), 113.8 (N*C*H), 70.9 (DME-*C*H$_2$), 59.3 (DME-*C*H$_3$), 32.8 (Si^{t}Bu$_3$), 31.3 (Si^{t}Bu$_2$), 31.1 (Si^{t}Bu$_2$), 28.9 (*C*H-^{i}Pr), 28.8 (*C*H-^{i}Pr), 24.8 (Si^{t}Bu$_2$), 24.7 (Si^{t}Bu$_2$), 24.4 (Si^{t}Bu$_3$), 23.9 (*C*H$_3$-^{i}Pr), -4.9 (Si*C*H$_3$). ^{29}**Si{^{1}H} NMR** (99.4 MHz, C_6D_6): *δ* = 43.6 (*Si*H), 23.3 (*Si*^{t}Bu$_3$), 3.3 (*Si*^{t}Bu$_2$)[a].

Melting point: 188.5°C

Notes: **a**: The assessment of the Si^{t}Bu$_2$ signal was verified by ^{1}H^{29}Si HMBC experiment.

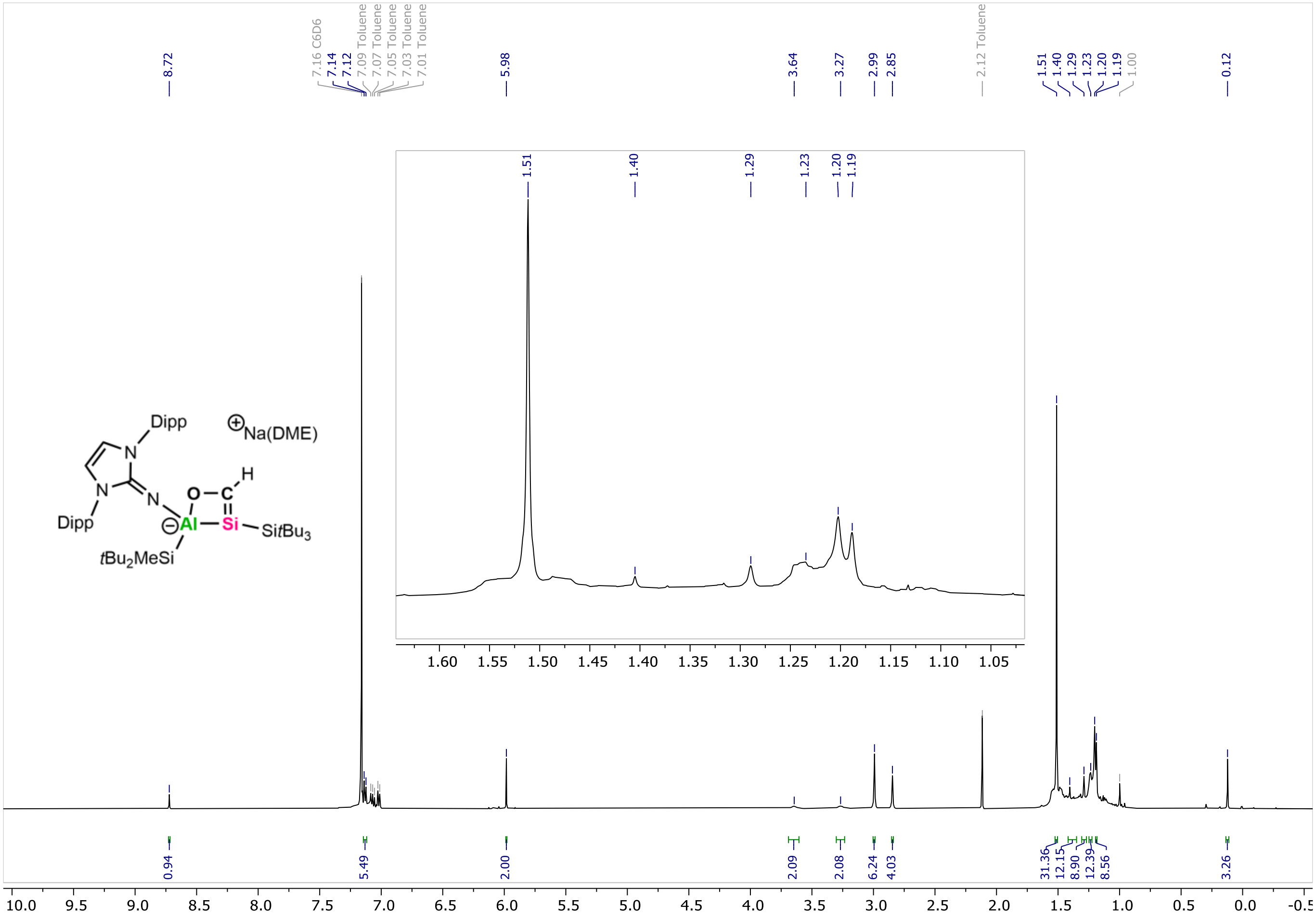

Figure S50: ^{1}H NMR spectrum (400.1 MHz, C_6D_6) of **6** including "zoom in" on the aliphatic region.

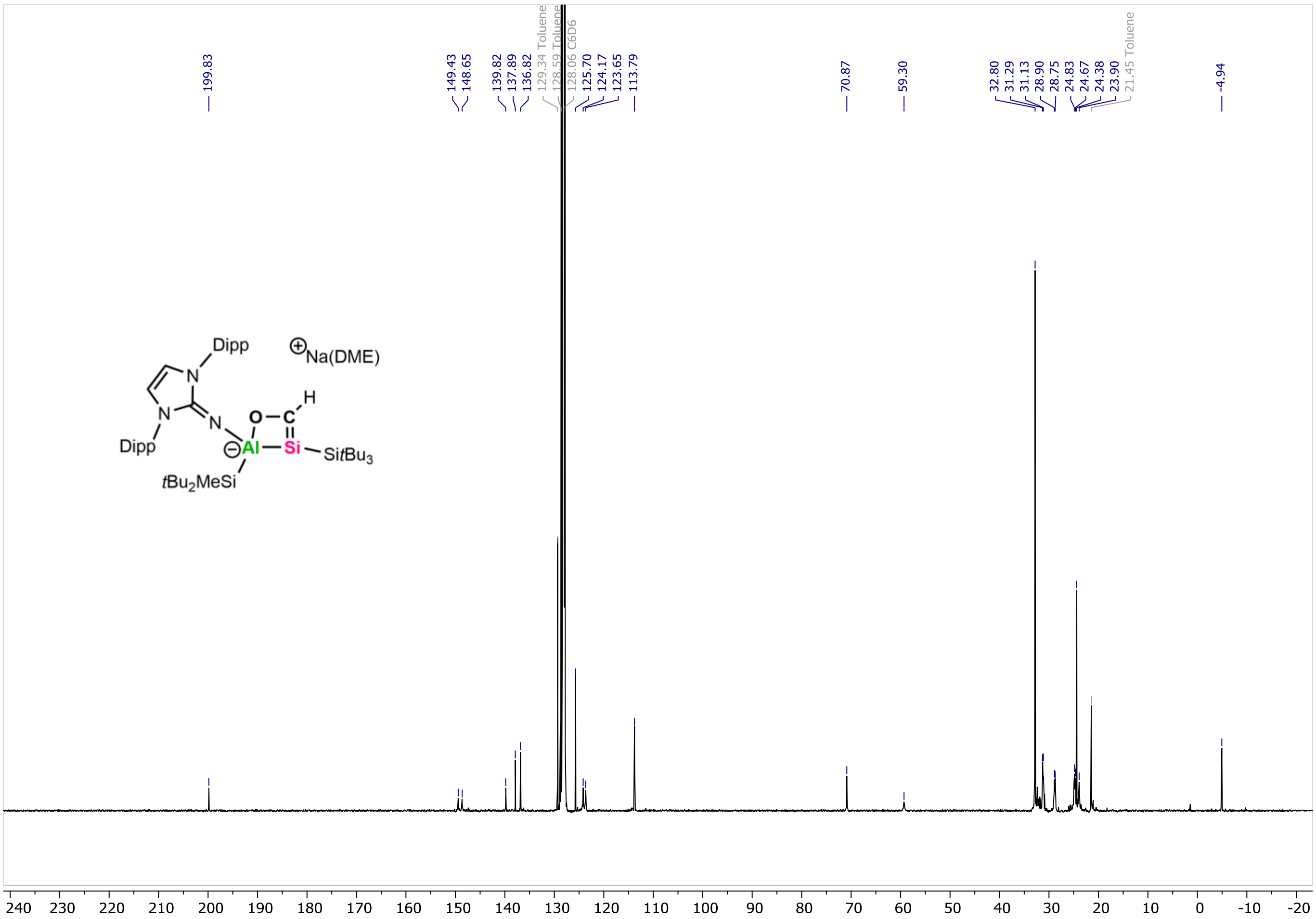

Figure S51: ^{13}C {^{1}H} NMR spectrum (100.6 MHz, C_6D_6) of **6**.

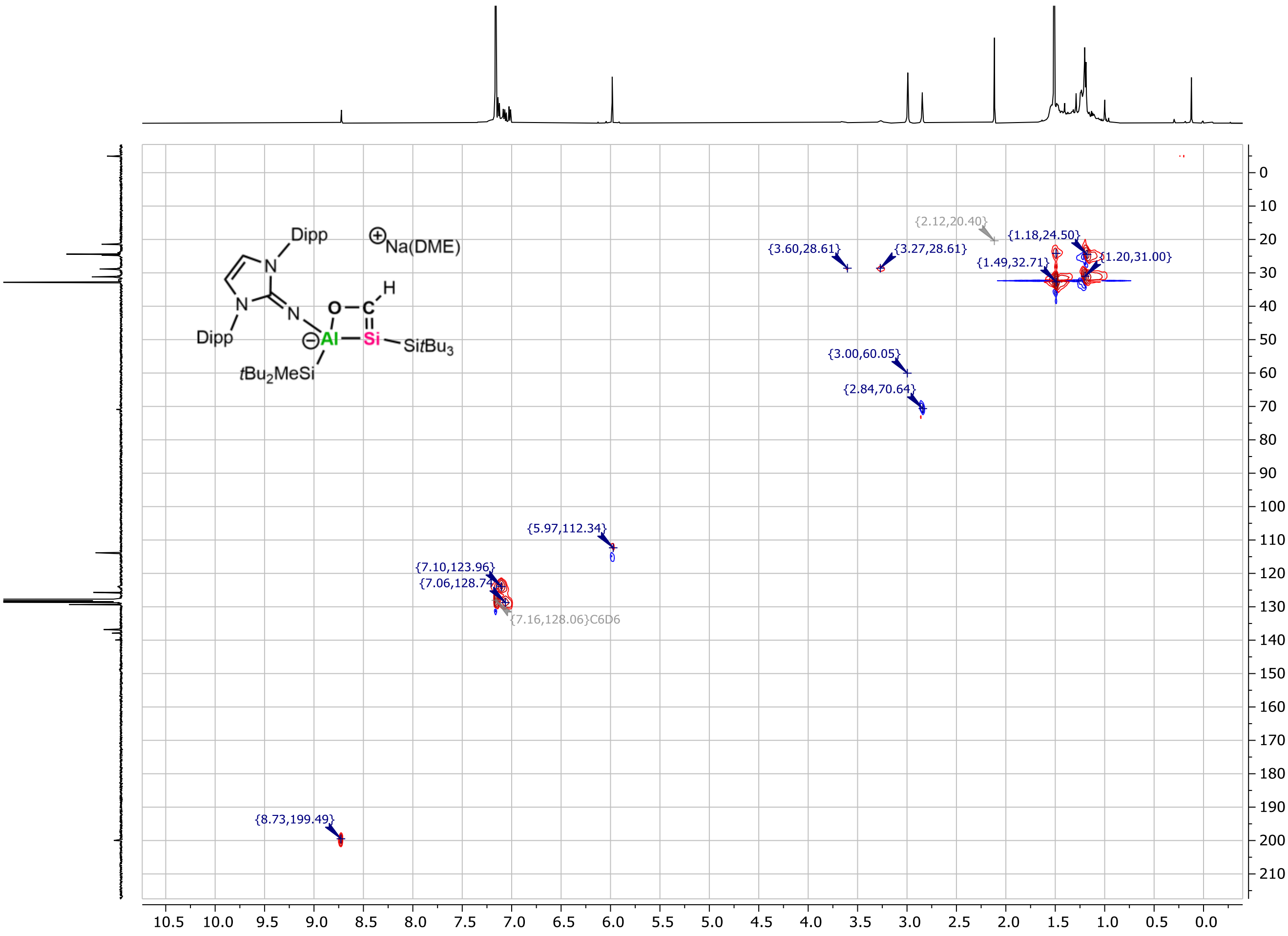

Figure S52: $^{1}H^{13}C$ HSQC NMR spectrum of **6** with $^{13}C\{^{1}H\}$ spectrum as vertical trace and ^{1}H spectrum as horizontal trace.

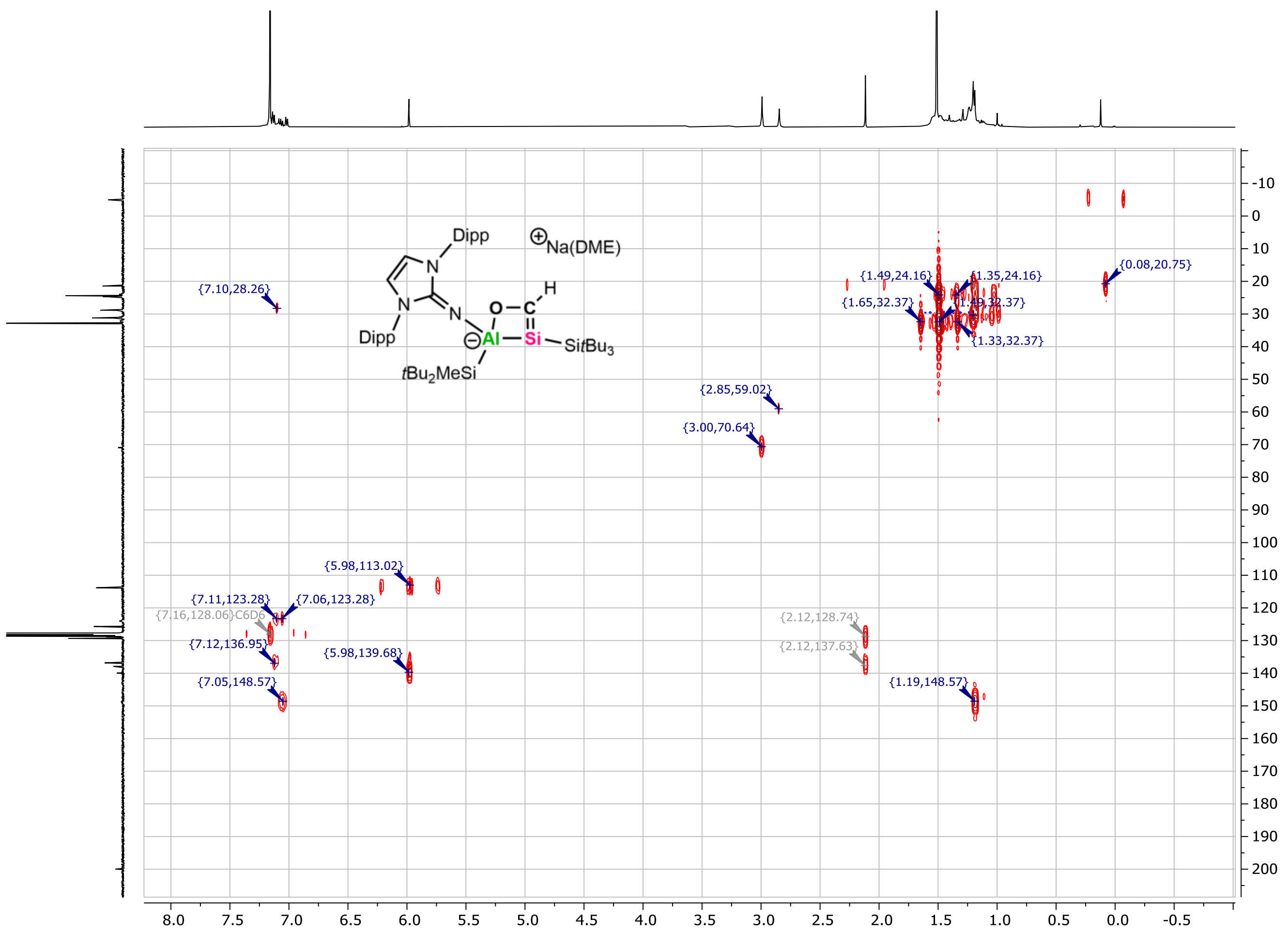

Figure S53: $^{1}H^{13}C$ HMBC NMR spectrum of **6** with $^{13}C\{^{1}H\}$ spectrum as vertical trace and ^{1}H spectrum as horizontal trace.

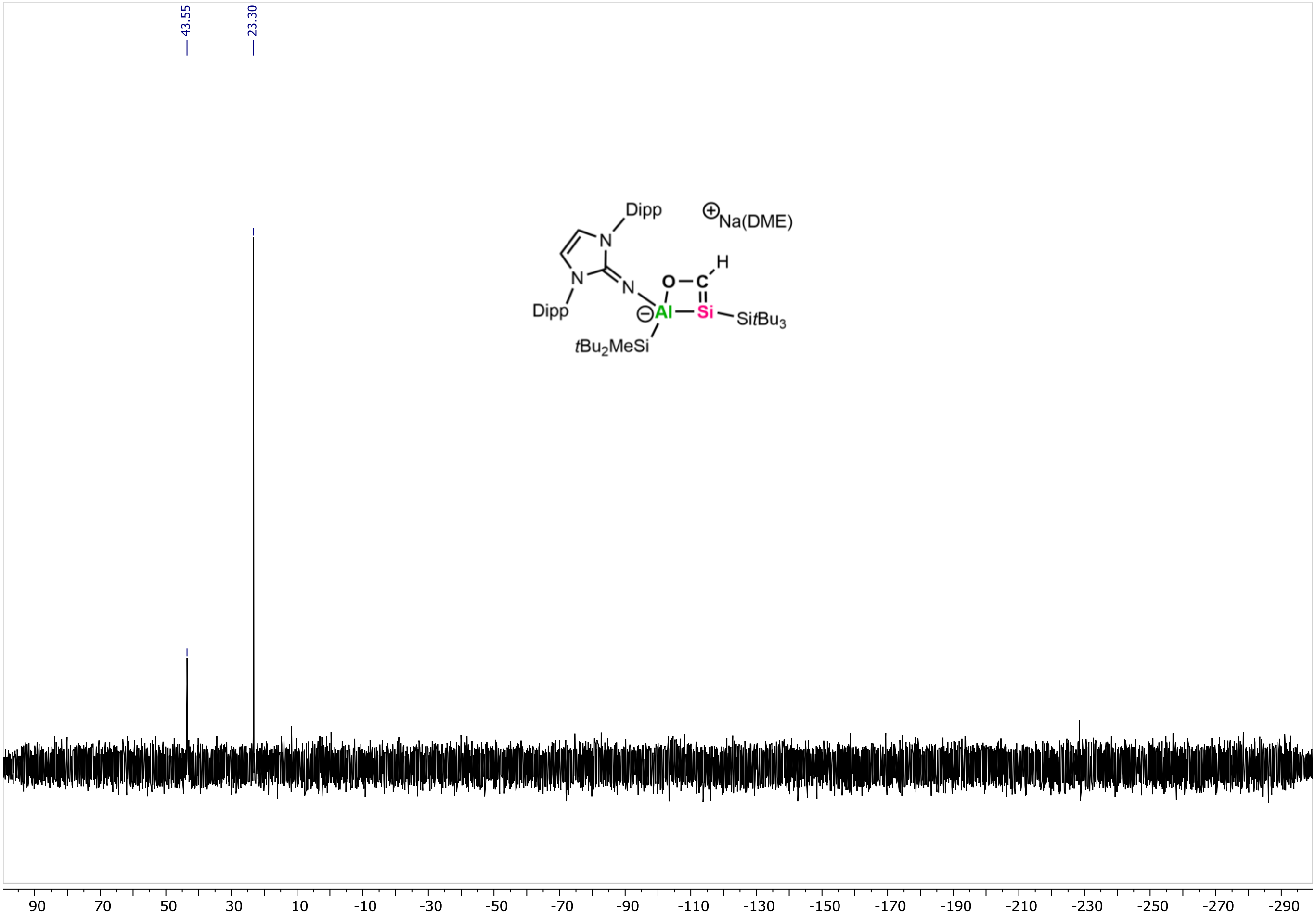

Figure S54: $^{29}Si\{^1H\}$ NMR spectrum (99.4 MHz, C_6D_6) of **6**.

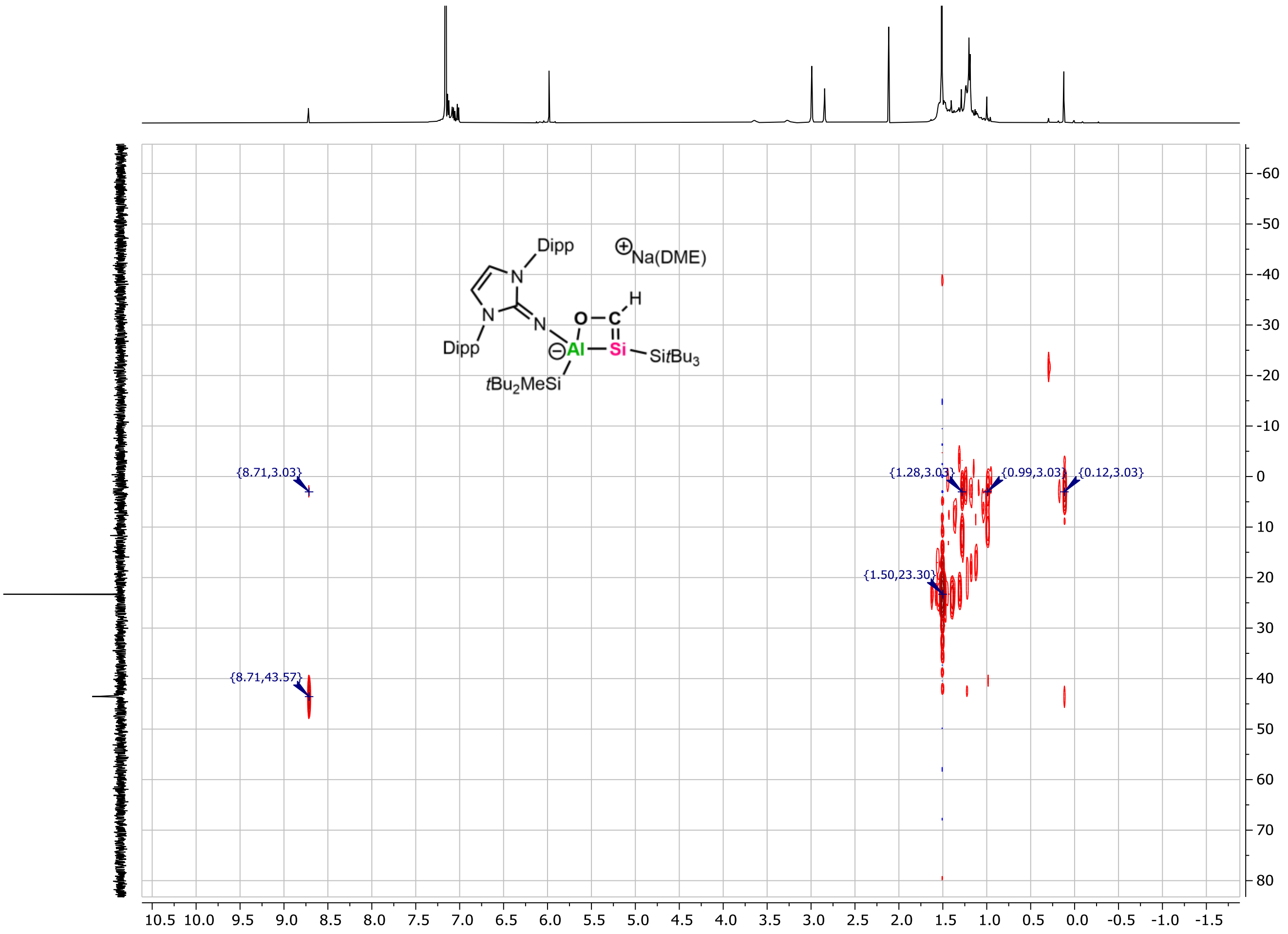

Figure S55: $^{1}H^{29}Si$ HMBC NMR spectrum of **6** with $^{29}Si\{^{1}H\}$ spectrum as vertical trace and ^{1}H spectrum as horizontal trace. The spectrum is referenced to the ***H*****Si*****Si***^{t}Bu3 cross-peak according to the ^{1}H and $^{29}Si\{^{1}H\}$ spectral data.

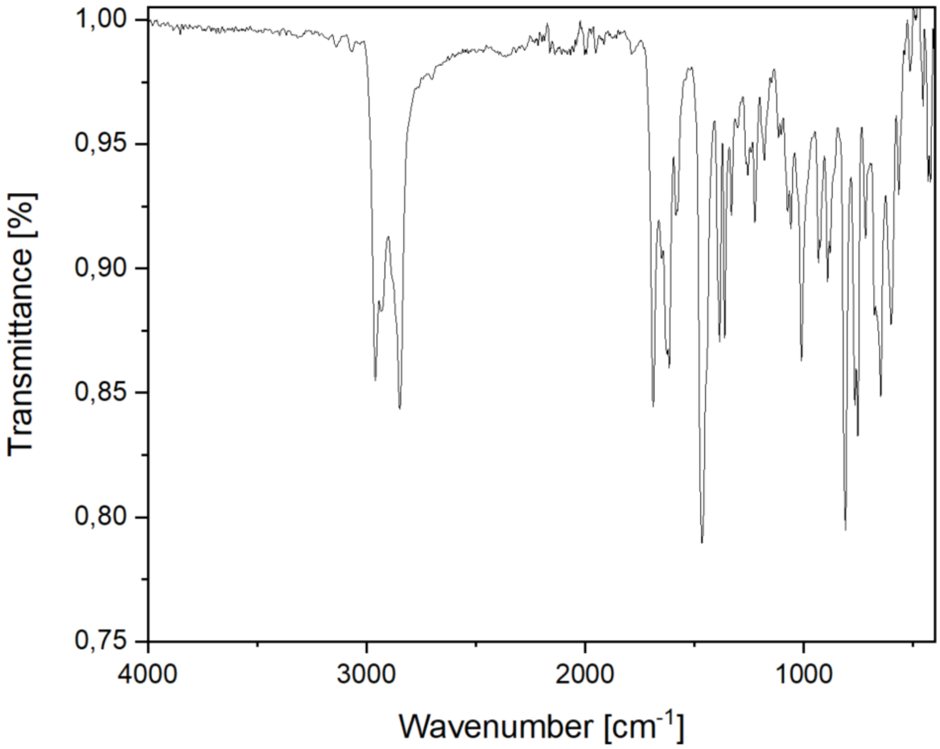

Figure S56: Solid-state FT-IR spectrum of **6**.

Synthesis of ^{13}C-labeled Cyclic Silacarbene (6^{13C})

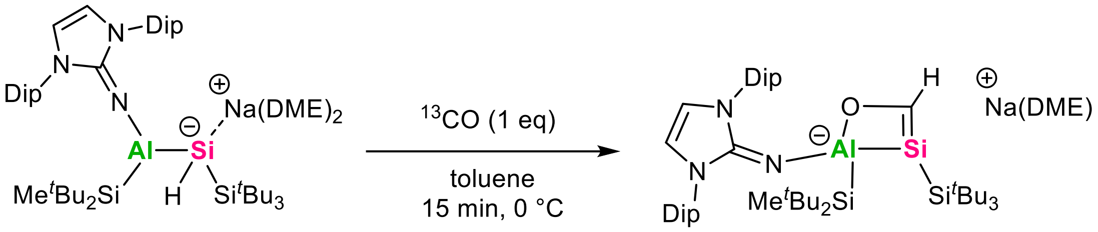

1a **6**^{13C}

For additional characteristic analytical data, the ^{13}C-labeled isotopomer **6**^{13C} was synthesized using the same procedure and ^{13}CO as described for the synthesis of **6**. Alumanyl silanide **1a** (30 mg, 32.3 µmol, 1 eq) was dissolved in toluene (2 mL) in a J-Young NMR tube with a total volume of 2.6 mL. Subsequently, ^{13}CO (1.5 bar) was added at 0 °C without degassing, after which the color changed from an initially orange to orange-yellow within 15 min. NMR analysis shows the selective formation of **6**^{13C}.

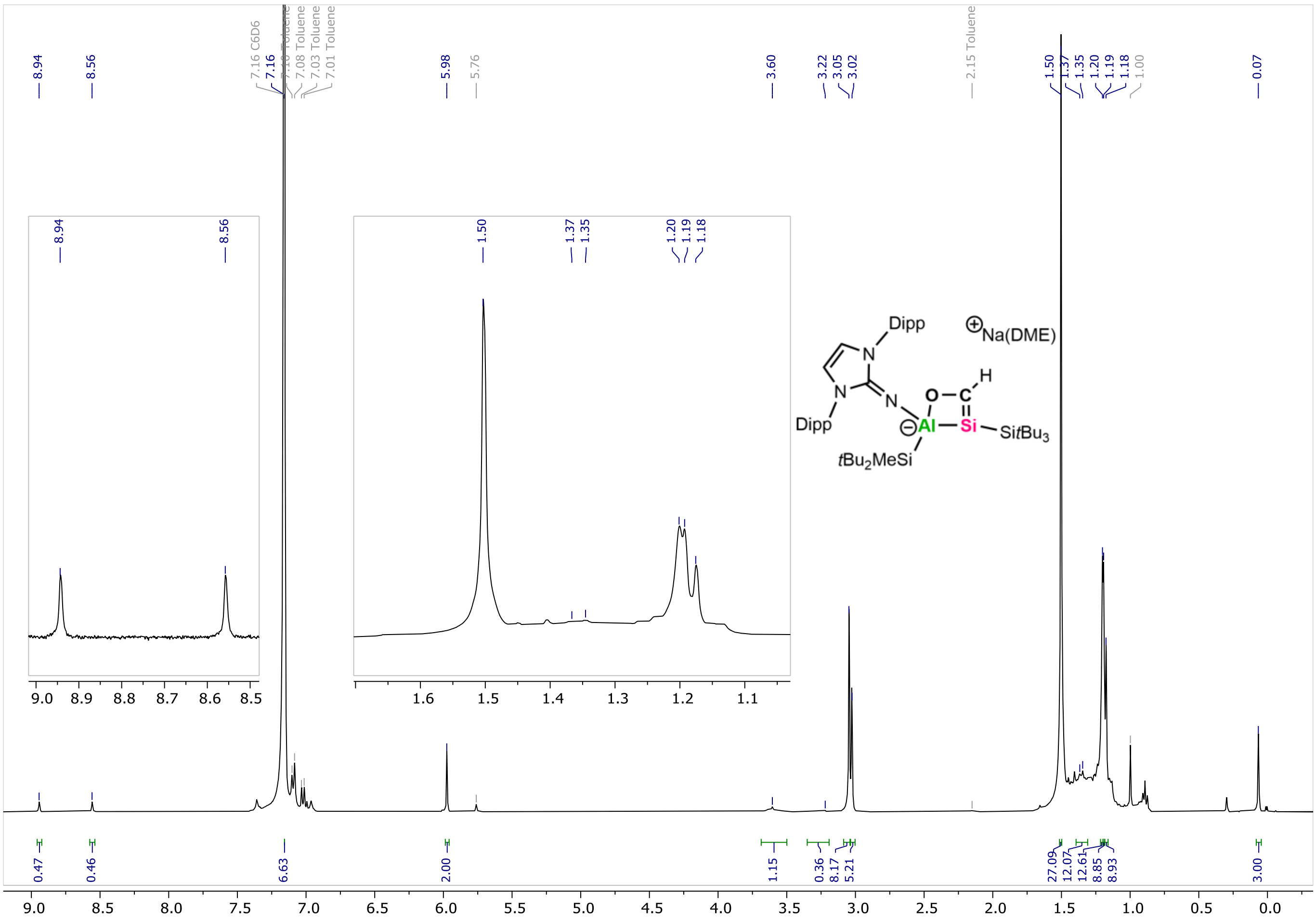

Figure S57: ^{1}H NMR spectrum (400.1 MHz, C_6D_6) of **6**^{13C} including "zoom in" on the C***H***O signal and the aliphatic region.

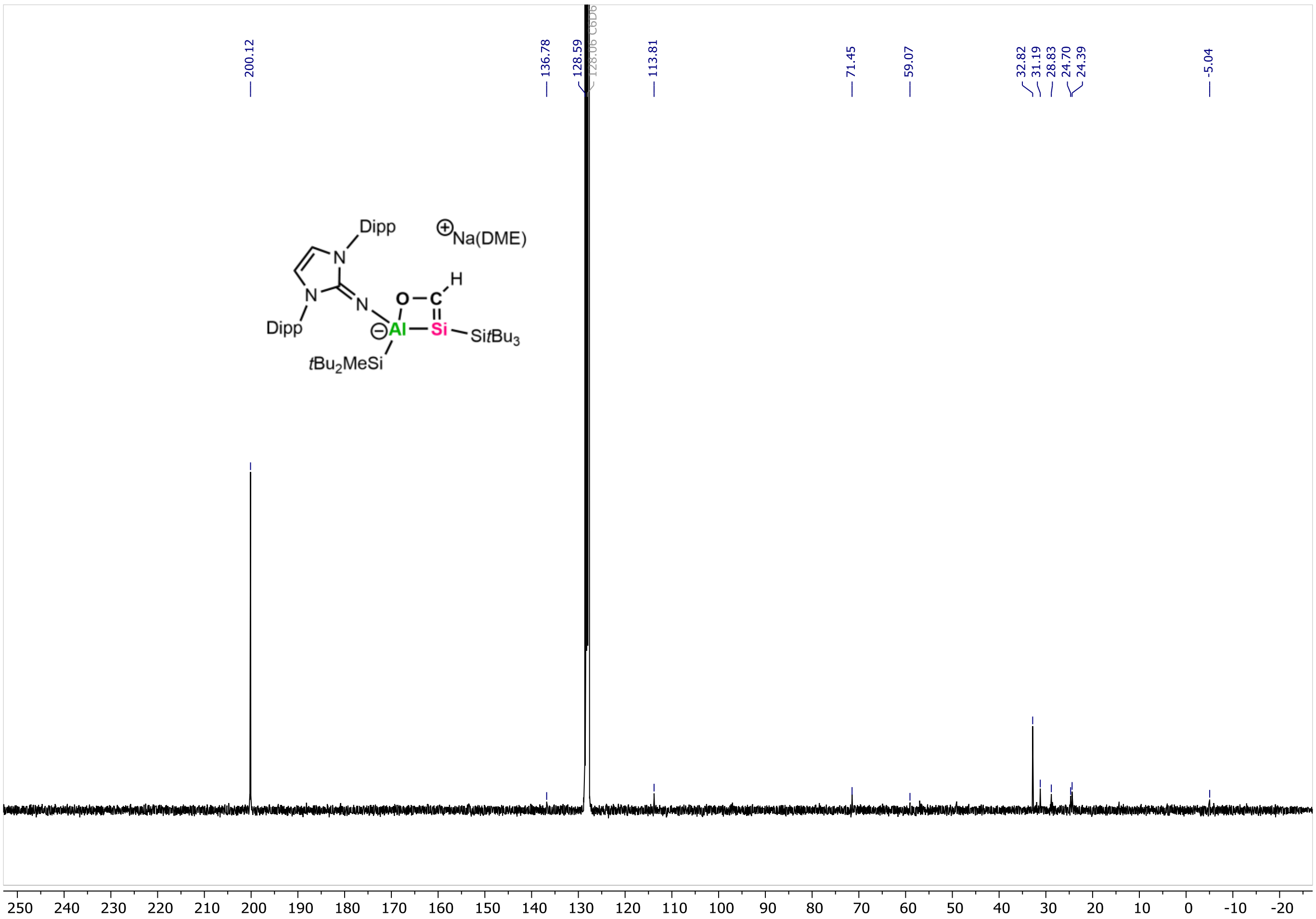

Figure S58: ^{13}C {^{1}H} NMR spectrum (100.6 MHz, C_6D_6) of **6**^{13C}.

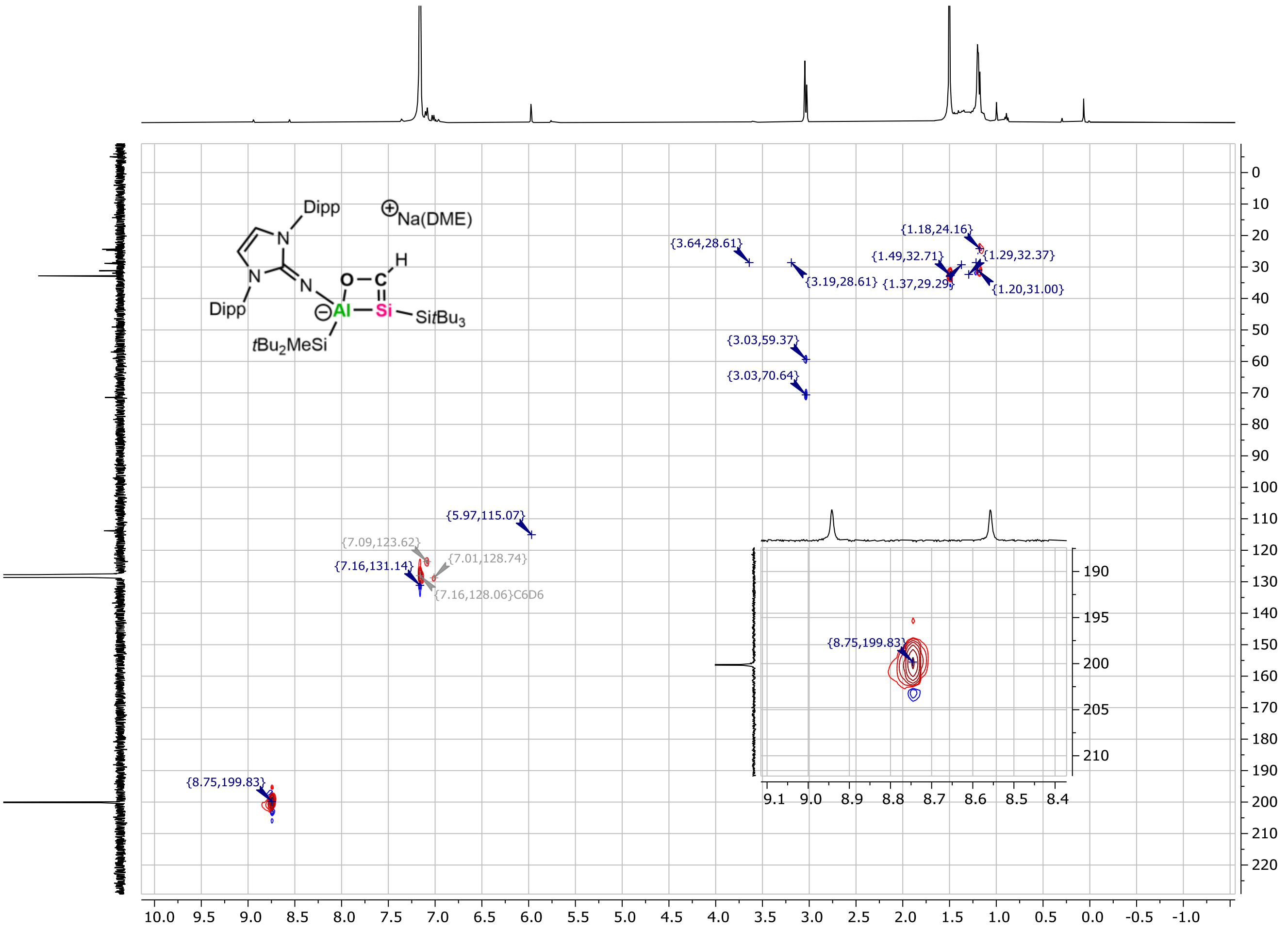

Figure S59: $^{1}H^{13}C$ HSQC NMR spectrum of **6**^{13C} including "zoom-in" on the ***CH*O** cross peak with $^{13}C\{^{1}H\}$ spectrum as vertical trace and ^{1}H spectrum as horizontal trace.

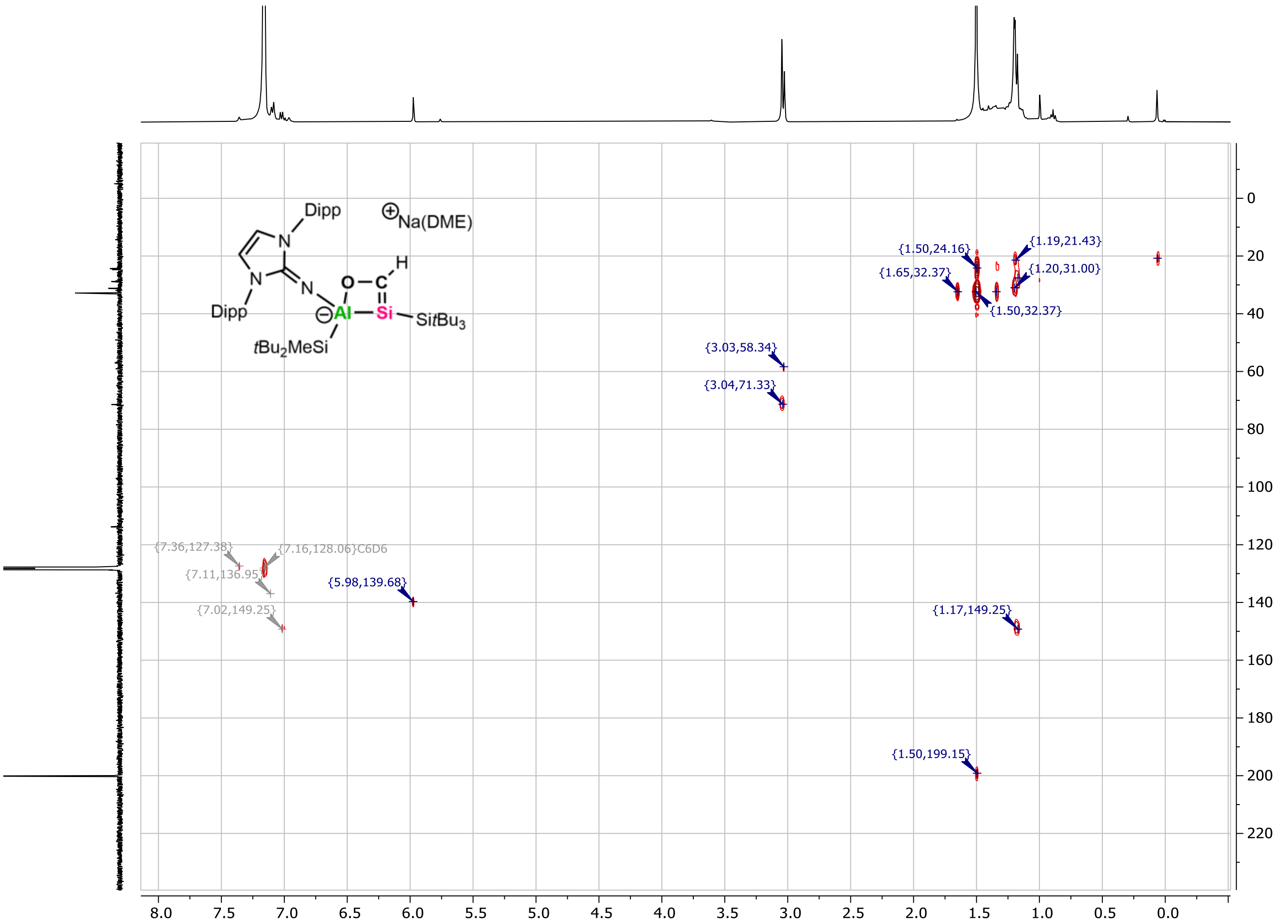

Figure S60: $^{1}H^{13}C$ HMBC NMR spectrum of **6**^{13C} with $^{13}C\{^{1}H\}$ spectrum as vertical trace and ^{1}H spectrum as horizontal trace.

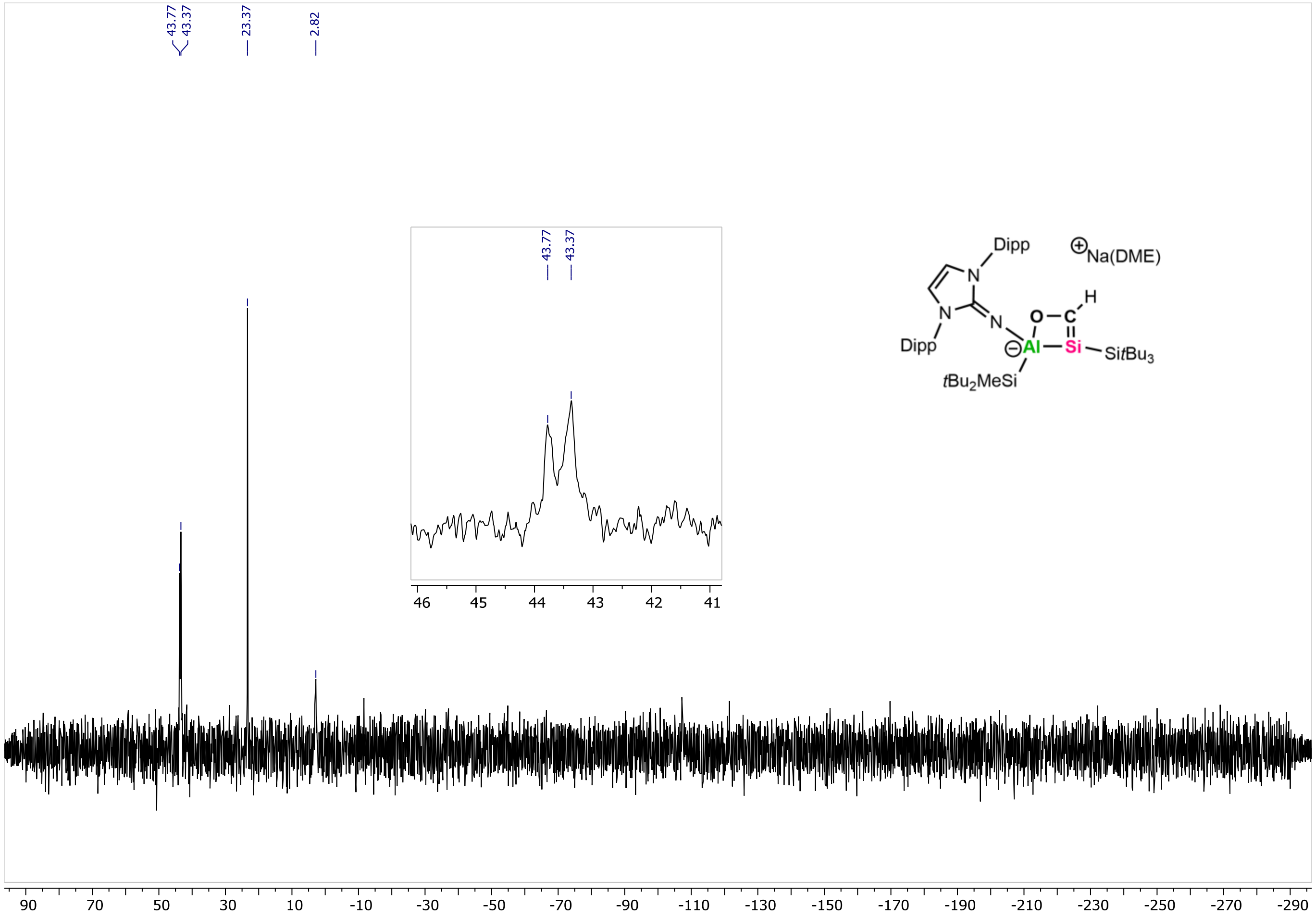

Figure S61: $^{29}Si\{^{1}H\}$ NMR spectrum (99.4 MHz, C_6D_6) of **6**^{13C} including “zoom-in” on the central silicon resonance. The central silicon signal appears as a doublet (^{1}J = 33 Hz) due to coupling with the adjacent ^{13}C-labeled nucleus.

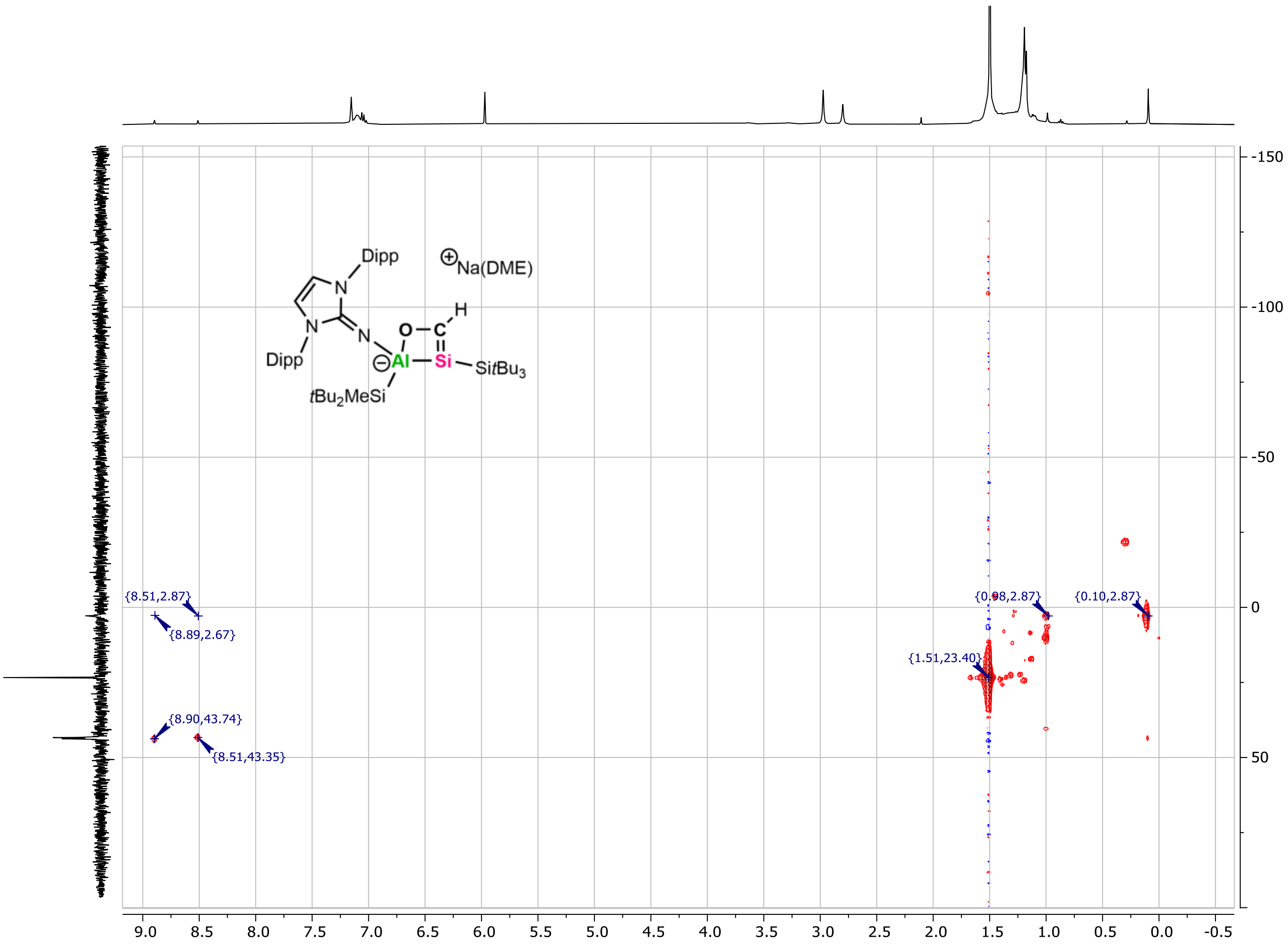

Figure S62: $^{1}H^{29}Si$ HMBC NMR spectrum of **6**^{13C} with $^{29}Si\{^{1}H\}$ spectrum as vertical trace and ^{1}H spectrum as horizontal trace. The spectrum is referenced to the ***H***Si***Si***^{t}Bu$_3$ cross-peak according to the ^{1}H and $^{29}Si\{^{1}H\}$ spectral data.

2.) Crystallographic Details

The X-ray intensity data were collected on an X-ray single crystal diffractometer equipped with a CMOS detector (Bruker Photon-100), a rotating anode (Bruker TXS) with MoKα radiation (λ = 0.71073 Å) and a Helios mirror optic by using the APEX4 software package[3] or an X-ray single crystal diffractometer equipped with a CMOS detector (Bruker Photon-100), an IMS microsource with MoKα radiation (λ = 0.71073 Å) and a Helios mirror optic by using the APEX4 software package.[3] The measurement was performed on single crystals coated with perfluorinated ether. The crystal was fixed on the top of a microsampler, transferred to the diffractometer and measured under a stream of cold nitrogen. A matrix scan was used to determine the initial lattice parameters. Reflections were merged and corrected for Lorenz and polarization effects, scan speed, and background using SAINT.[4] Absorption corrections, including odd and even ordered spherical harmonics were performed using SADABS.[5] Space group assignments were based upon systematic absences, E statistics, and successful refinement of the structures. Structures were solved by direct methods with the aid of successive difference Fourier maps, and were refined against all data using the APEX4[3] in conjunction with SHELXL-2018/3.[6, 7] and SHELXLE.[8] Methyl hydrogen atoms were refined as part of rigid rotating groups, with a C–H distance of 0.98 Å and Uiso(H) = 1.5·Ueq(C). Other H atoms were placed in calculated positions and refined using a riding model, with methylene and aromatic C-H distances of 0.99 and 0.95 Å, respectively, and Uiso(H) = 1.2·Ueq(C). H1 in **2a**, **5**, and **7** could be located in the difference Fourier maps and were allowed to refine freely. If not mentioned otherwise, non-hydrogen atoms were refined with anisotropic displacement parameters. PLATON SQUEEZE was used to remove a highly disordered toluene molecule from crystal structure **2a,** highly disordered benzene and pentane molecules from crystal structure **7,** and a highly disordered toluene molecule from crystal structure **8**.[9] Full-matrix least-squares refinements were carried out by minimizing $\Delta w(Fo^2\text{-}Fc^2)^2$ with SHELXL-2014[10] weighting scheme. Neutral atom scattering factors for all atoms and anomalous dispersion corrections for the non-hydrogen atoms were taken from International Tables for Crystallography.[11] Images of the crystal structures were generated by PLATON and MERCURY.[12, 13] The CCDC numbers CCDC-2499949 – 2499954 contain the supplementary crystallographic data for the structures **2a**, **5**, **6**, **7**, **8**, and **6’**. These data can be obtained free of charge from the Cambridge Crystallographic Data Centre via https://www.ccdc.cam.ac.uk/structures/. The crystallographic information files (CIF) were generated using FinalCif.[14]

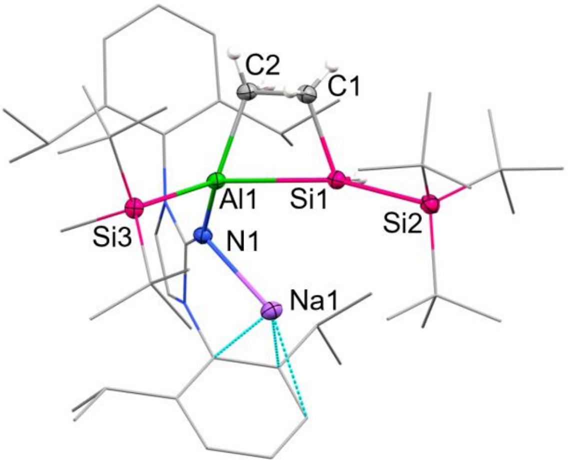

Figure S63: Ellipsoid plot (20% level) of the molecular structure of **2a** in the solid state (from SC-XRD analysis). Solvent and hydrogen atoms are omitted; except on Si1, C1 and C2. Selected alkyl and aryl groups are shown as wireframe models.

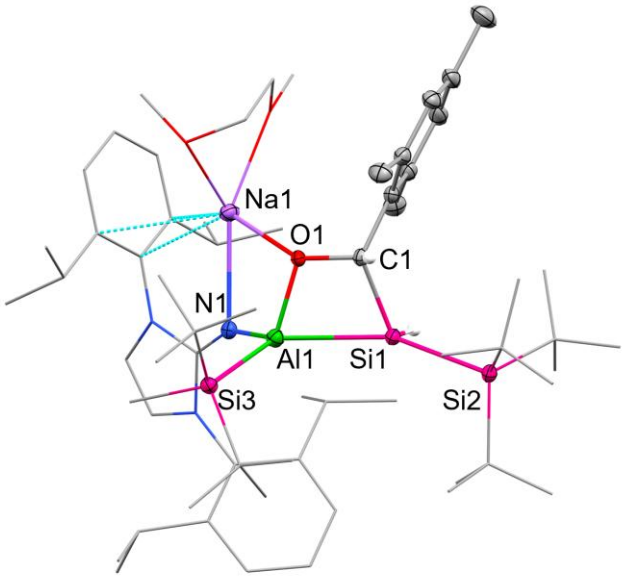

Figure S64: Ellipsoid plot (20% level) of the molecular structure of **5** in the solid state (from SC-XRD analysis). Solvent and hydrogen atoms are omitted; except on Si1 and C1. Selected alkyl and aryl groups are shown as wireframe models. Selected bond lengths [Å] and angles [°]: Al1–Si1 = 2.5502(5), Al1–Si3 = 2.5502(5), Al1–O1 = 1.8125(8), O1–C1 = 1.4603(13), Si1–C1 = 1.9690(11), Si1–Si2 = 2.4032(4), O1–Al1–Si1 = 72.98(3), Al1–Si1–C1 = 71.10(3), Si1–C1–C2 = 100.76(6), C1–O1–Al1 = 108.84(6).

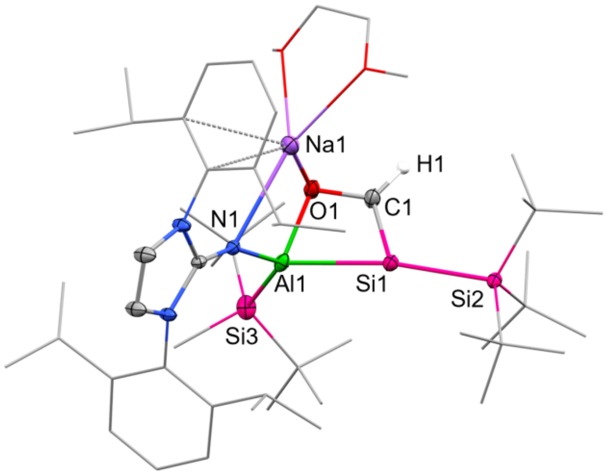

Figure S65: Ellipsoid plot (20% level) of the molecular structure of **6** in the solid state (from SC-XRD analysis). Solvent and hydrogen atoms are omitted; except on C1. Selected alkyl and aryl groups are shown as wireframe models. Selected bond lengths [Å] and angles [°]: Al1–Si1 = 2.5596(12), Al1–Si3 = 2.5358(14), Al1–O1 = 1.840(3), O1–C1 = 1.394(4), Si1–C1 = 1.781(4), Si1–Si2 = 2.3841(11), O1–Al1–Si1 = 72.97(8), Al1–Si1–C1 = 68.44(12), Si1–C1–O1 = 114.6(2), C1–O1–Al1 = 101.8(2).

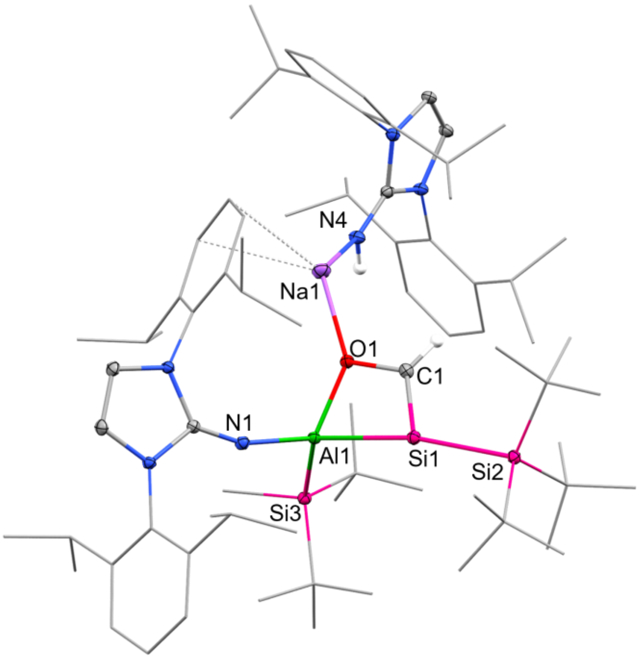

Figure S66: Ellipsoid plot (20% level) of the molecular structure of **6’** in the solid state (from SC-XRD analysis). The assumed side-product along the decomposition pathway of **6** was crystallized from gas-phase diffusion of hexane into a concentrated toluene solution (20 mg of **1a**), which was pressurized with CO (1 bar) beforehand, at -35 °C. Solvent and hydrogen atoms are omitted; except on C1. Selected alkyl and aryl groups are shown as wireframe models. Selected bond lengths [Å] and angles [°]: Al1–Si1 = 2.5143(7), Al1–Si3 = 2.4783(7), Al1–O1 = 1.8333(13), O1–C1 = 1.405(2), Si1–C1 = 1.7701(19), Si1–Si2 = 2.3652(7), O1–Al1–Si1 = 74.02(4), Al1–Si1–C1 = 68.92(6), Si1–C1–O1 = 114.18(13), C1–O1–Al1 = 100.27(10).

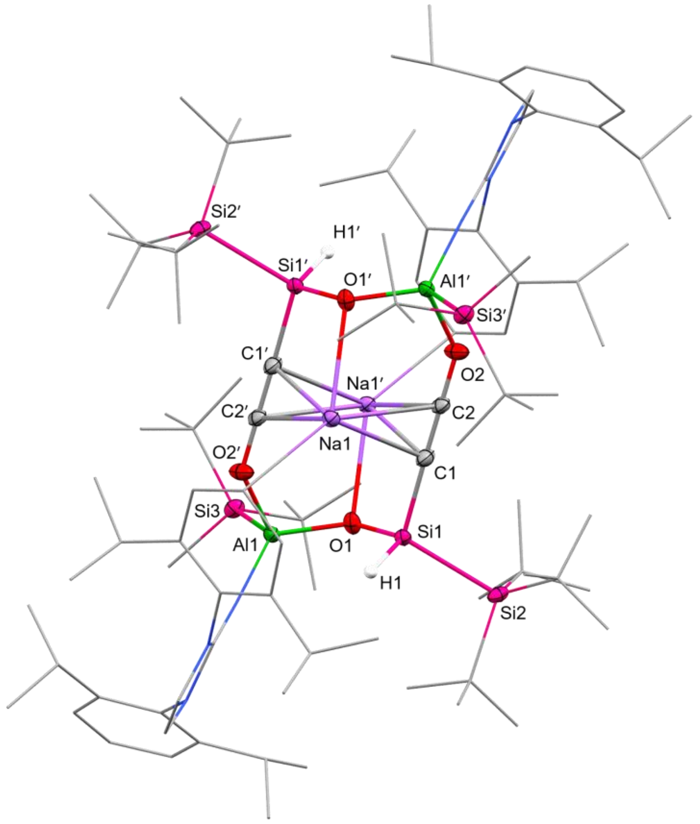

Figure S67: Ellipsoid plot (20% level) of the molecular structure of **7** in the solid state (from SC-XRD analysis). Solvent and hydrogen atoms are omitted; except on Si1. Selected alkyl and aryl groups are shown as wireframe models. Symmetry-generated atoms are marked with prime. Selected bond lengths [Å] and angles [°]: Al1–O1 = 1.744(3), O1–Si1 = 1.627(3), Al1–Si3 = 2.5707(16), Si1–Si2 = 2.4529(13), Si1–C1 = 1.864(3), C1–C2 = 1.219(4), C2–O2 = 1.253(4), Al1–O2 = 1.867(2), Al1–O1–Si1 = 133.00(19).

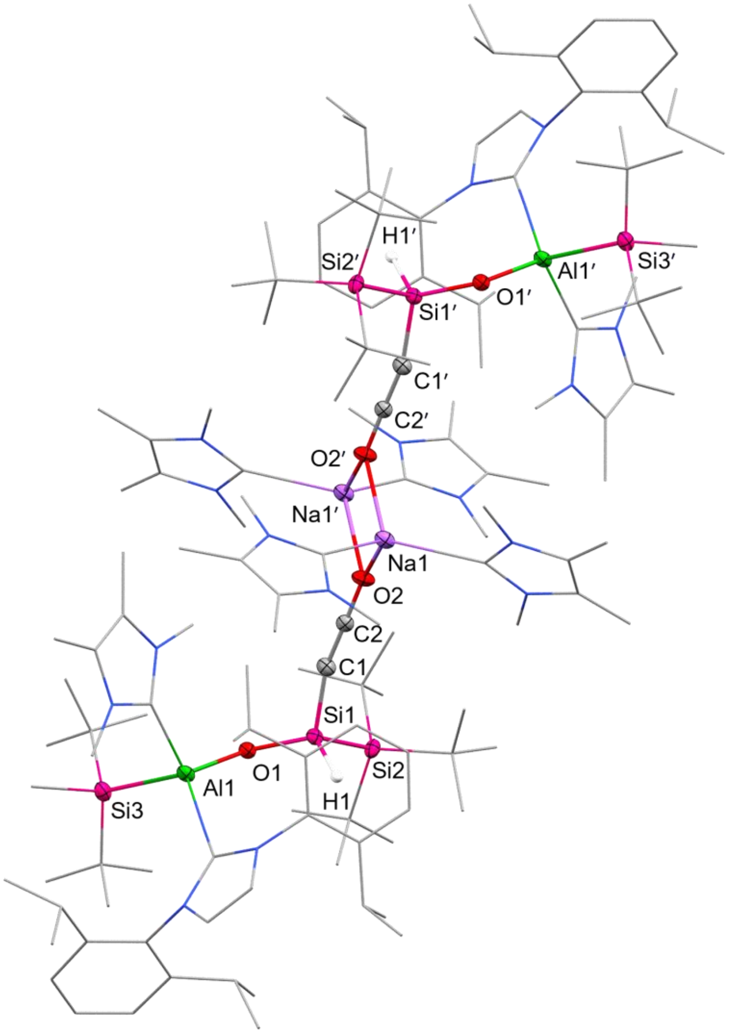

Figure S68: Ellipsoid plot (20% level) of the molecular structure of **8** in the solid state (from SC-XRD analysis). Solvent and hydrogen atoms are omitted; except on Si1. Symmetry-generated atoms are marked with prime. Selected alkyl and aryl groups are shown as wireframe models. Selected bond lengths [Å] and angles [°]: Al1–O1 = 1.7399(13), O1–Si1 = 1.6300(13), Al1–Si3 = 2.5393(7), Si1–Si2 = 2.3694(7), Si1–C1 = 1.7891(19), C1–C2 = 1.219(3), C2–O2 = 1.247(2), Al1–O1–Si1 = 140.46(8).

Table 1: Experimental details for SC-XRD and structure refinement.

	**2a**	**5**	**6**
**CCDC number**	2499949	2499950	2499951
**Empirical formula**	$C_{50}H_{89}AlN_3NaSi_3$	$C_{65}H_{110}AlN_3NaO_3Si_3$	$C_{53}H_{95}AlN_3NaO_3Si_3$
**Formula weight**	866.48	1115.79	956.55
**Temperature [K]**	100(2)	100(2)	100(2)
**Crystal system**	monoclinic	triclinic	monoclinic
**Space group (number)**	$P2_1/n$ (14)	$P\bar{1}$ (2)	$C2/c$ (15)
a **[Å]**	12.7332(14)	13.4662(11)	40.718(2)
b **[Å]**	19.7139(19)	15.2603(14)	12.2330(6)
c **[Å]**	22.965(3)	18.1791(16)	24.1961(13)
**α [°]**	90	91.518(3)	90
**β [°]**	98.091(4)	92.963(3)	104.607(3)
**γ [°]**	90	115.278(3)	90
**Volume [$Å^3$]**	5707.3(11)	3368.7(5)	11662.6(11)
Z	4	2	8
**ρ_{calc} [gcm^{-3}]**	1.008	1.100	1.090
**μ [mm^{-1}]**	0.138	0.133	0.144
***F*(000)**	1904	1222	4192
**Crystal size [mm^3]**	0.181×0.231×0.245	0.287×0.474×0.535	0.119×0.196×0.285
**Crystal color**	colorless	colorless	yellow
**Crystal shape**	fragment	block	block
**Radiation**	MoK_α (λ=0.71073 Å)	MoK_α (λ=0.71073 Å)	MoK_α (λ=0.71073 Å)
**2θ range [°]**	3.91 to 50.70 (0.83 Å)	4.49 to 51.34 (0.82 Å)	3.78 to 51.36 (0.82 Å)
**Index ranges**	$-15 \leq h \leq 15$<br>$-23 \leq k \leq 23$<br>$-27 \leq l \leq 27$	$-16 \leq h \leq 16$<br>$-18 \leq k \leq 18$<br>$-22 \leq l \leq 22$	$-49 \leq h \leq 49$<br>$-14 \leq k \leq 14$<br>$-29 \leq l \leq 29$
**Reflections collected**	162669	147796	168105
**Independent reflections**	10436<br>R_{int} = 0.0778<br>R_{sigma} = 0.0285	12671<br>R_{int} = 0.0524<br>R_{sigma} = 0.0310	11040<br>R_{int} = 0.0691<br>R_{sigma} = 0.0295
**Completeness to θ = 25.242°**	99.9 %	99.2 %	99.8 %
**Data / Restraints /**	10436 / 1398 / 681	12671 / 79 / 748	11040 / 1391 / 703

Parameters			
Absorption correction T_{min}/T_{max} (method)	0.7027 / 0.7453 (multi-scan)	0.7081 / 0.7453 (multi-scan)	0.7006 / 0.7453 (multi-scan)
Goodness-of-fit on F^2	1.056	1.055	1.026
Final R indexes [I≥2σ(I)]	R_1 = 0.0919 wR_2 = 0.2419	R_1 = 0.0363 wR_2 = 0.0996	R_1 = 0.0791 wR_2 = 0.2217
Final R indexes [all data]	R_1 = 0.1485 wR_2 = 0.3434	R_1 = 0.0374 wR_2 = 0.1008	R_1 = 0.0916 wR_2 = 0.2373
Largest peak/hole [eÅ^{-3}]	0.82/−0.41	0.51/−0.44	1.72/−0.77

	7	8	6‘
CCDC number	2499952	2499953	2499954
Empirical formula	$C_{142}H_{242}Al_2N_{18}Na_2O_4Si_6$	$C_{100}H_{170}Al_2N_6Na_2O_4Si_6$	$C_{76}H_{122}AlN_6NaOSi_3$
Formula weight	2534.00	1788.89	1270.03
Temperature [K]	100(2)	100(2)	100(2)
Crystal system	triclinic	monoclinic	monoclinic
Space group (number)	$P\overline{1}$ (2)	$P2_1/n$ (14)	$P2_1/c$ (14)
a [Å]	14.5099(7)	16.0418(8)	22.679(3)
b [Å]	16.7775(8)	19.1540(10)	12.3747(15)
c [Å]	21.5228(10)	18.3143(9)	29.100(4)
α [°]	88.188(2)	90	90
β [°]	79.160(2)	91.533(2)	105.380(4)
γ [°]	76.431(2)	90	90
Volume [Å^3]	5002.0(4)	5625.3(5)	7874.3(17)
Z	1	2	4
ρ_{calc} [gcm^{-3}]	0.841	1.056	1.071
μ [mm^{-1}]	0.096	0.144	0.121
F(000)	1384	1952	2776
Crystal size [mm^3]	0.244×0.260×0.318	0.208×0.254×0.285	0.114×0.143×0.168
Crystal color	colorless	colorless	colorless
Crystal shape	fragment	block	block
Radiation	MoK_α (λ=0.71073 Å)	MoK_α (λ=0.71073 Å)	MoK_α (λ=0.71073 Å)

**2θ range [°]**	4.57 to 50.05 (0.84 Å)	4.25 to 51.41 (0.82 Å)	3.84 to 50.05 (0.84 Å)
**Index ranges**	$-17 \le h \le 17$<br>$-19 \le k \le 19$<br>$-25 \le l \le 25$	$-19 \le h \le 19$<br>$-23 \le k \le 23$<br>$-22 \le l \le 22$	$-26 \le h \le 26$<br>$-14 \le k \le 14$<br>$-34 \le l \le 34$
**Reflections collected**	101413	246124	394797
**Independent reflections**	17623<br>R_{int} = 0.0437<br>R_{sigma} = 0.0300	10707<br>R_{int} = 0.0575<br>R_{sigma} = 0.0214	13907<br>R_{int} = 0.3944<br>R_{sigma} = 0.0926
**Completeness to θ = 25.242°**	99.8 %	99.9 %	99.9 %
**Data / Restraints / Parameters**	17623 / 2847 / 1193	10707 / 1420 / 737	13907 / 72 / 862
**Absorption correction T_{min}/T_{max} (method)**	0.6802 / 0.7453<br>(multi-scan)	0.7087 / 0.7453<br>(multi-scan)	0.6817 / 0.7453<br>(multi-scan)
**Goodness-of-fit on F^2**	1.033	1.049	0.976
**Final *R* indexes [$I \geq 2\sigma(I)$]**	R_1 = 0.0503<br>wR_2 = 0.1519	R_1 = 0.0711<br>wR_2 = 0.1925	R_1 = 0.0457<br>wR_2 = 0.1151
**Final *R* indexes [all data]**	R_1 = 0.0609<br>wR_2 = 0.1631	R_1 = 0.0763<br>wR_2 = 0.1978	R_1 = 0.0646<br>wR_2 = 0.1221
**Largest peak/hole [eÅ^{-3}]**	0.40/−0.19	0.76/−0.59	0.43/−0.60

CCDC XXXXXXX-YYYYYYY (generally used for organic and metal-organic structures)

CSD XXXXXXX-YYYYYYY (generally used for inorganic structures)

Deposition Number 2499949-2499954

Summary of Data - Deposition Number 2499949

Compound Name:
Data Block Name: data_mo_LudMo17_0m_sq
Unit Cell Parameters: a 12.7332(14) b 19.7139(19) c 22.965(3) P21/n
----

Summary of Data - Deposition Number 2499950

Compound Name:
Data Block Name: data_LudMo13_0m
Unit Cell Parameters: a 13.4662(11) b 15.2603(14) c 18.1791(16) P-1

Summary of Data - Deposition Number 2499951

Compound Name:
Data Block Name: data_LudMo29_0ma
Unit Cell Parameters: a 40.718(2) b 12.2330(6) c 24.1961(13) C2/c
----

Summary of Data - Deposition Number 2499952

Compound Name:
Data Block Name: data_LudMo24_0m_sq
Unit Cell Parameters: a 14.5099(7) b 16.7775(8) c 21.5228(10) P-1

Summary of Data - Deposition Number 2499953

Compound Name:
Data Block Name: data_LudMo26_0m_sq
Unit Cell Parameters: a 16.0418(8) b 19.1540(10) c 18.3143(9) P21/n
----

Summary of Data - Deposition Number 2499954

Compound Name:
Data Block Name: data_LudMo18_0m
Unit Cell Parameters: a 22.679(3) b 12.3747(15) c 29.100(4) P21/c
----

3.) Details of Theoretical Calculations

The geometry optimizations were performed with the Gaussian16 (Revision C.02) program[15] using the PBE0 hybrid exchange functional[16-18] and Def2-SVP basis set.[19, 20] In addition, Grimme's empirical dispersion correction with Becke-Johnson damping (GD3BJ)[21] was used as well as an ultrafine integration grid. Full analytical frequency calculations were performed for the optimized structures to ensure the nature of the stationary points found (minima, no imaginary frequencies or a transition state with one imaginary frequency). The Gibbs free energies were corrected by single-point calculations employing the SMD (benzene or toluene (mechanism 6Na)) solvent model[22] at the SMD-PBE0-GD3BJ/Def2-TZVP level. The used computational method is thus described as SMD-PBE0-GD3BJ/Def2-TZVP//PBE0-GD3BJ/Def2-SVP. The reported Gibbs free energies are additionally corrected with 7.90 kJ mol^{-1} for standard state conditions.

Bonding analyses were done for the optimized structures using AIMAll (QTAIM)[23] and the NBO calculations were performed with NBO3.1 as implemented in Gaussian16.

Comparison of experimental and computational structures

Compound **1** was optimized as an anion and with $Na(DME)_2^+$ and Na^+ as the counter cations (Table 2). Comparison of the here optimized structures to the experimental and previously reported computational data were in good agreement. The naked anionic structure exhibits slight deviation in the bond parameters (**1**$^-$ vs **1Na.crypt.**), but this is expected due to the absence of the (sterically demanding) counter cation stabilized by a crown, DME or cryptand. The optimized structures of **2a**, **5** and **6** are in good agreement with the experimental data.

Table 2: Comparison of experimentally determined (XRD) and calculated bond metrics. Calculated structures at the PBE0-GD3BJ/Def2-SVP level.

Compound	Al-Si (Å)	Al-N(NHI) (Å)	Si-X (Å)	Al-Y (Å)	X-Y (Å)	Si-Al-N (°)
**1Na.crypt.**, exp. (ref. 7)	2.4179(10)	1.7778(17)	-	-	-	133.58(6)
**1**$^-$, comp.	2.390	1.796	-	-	-	128.80
**1Na(DME)$_2$**, comp.	2.407	1.776	-	-	-	130.36
**1Na**, comp.	2.397	1.810	-	-	-	112.99
**1Na.crypt.** comp. (ref. 7)	2.407	1.774	-	-	-	133.38
**2a**, exp. (X,Y = C)	2.600(6)	1.831(4)	1.926(6)	1.999(5)	1.537(8)	108.38(14)
**2a**, comp.	2.608	1.855	1.930	2.007	1.543	105.17
**5**, exp. (X=C, Y=O)	2.551(10)	1.831	1.969	1.813	1.461	125.43
**5**, comp.	2.530	1.855	1.976	1.850	1.427	125.779
**6**, exp. (X=C, Y=O)	2.514(6)	1.780	1.769	1.833	1.405	121.91
**6**, comp.	2.479	1.785	1.785	1.887	1.358	129.04

Frontier Molecular Orbitals (FMOs), Electronic structures and bonding analyses

Table 3: Calculated FMO energies (SMD-PBE0-GD3BJ/Def2-TZVP//PBE0-GD3BJ/Def2-SVP).

Compound	HOMO, eV	LUMO, eV	HOMO-LUMO gap, eV	Remarks
**1**$^-$ (anion)	-2.813	0.463	3.28	HOMO: delocalized, part Si lone pair, part Si-Al bond
**2a**	-4.633	-0.813	3.82	HOMO: delocalized, part N lone pair, part Si centered
**5**	-4.576	-0.592	3.98	HOMO: σ-bond Al-Si(^{t}Bu$_2$Me) and Si lone pair
**6**	-4.046	-0.555	3.49	HOMO: Si-C π-bond

QTAIM and NBO

Both QTAIM and NBO analyses were conducted on the optimized structures of **2a**, **5** and **6**. The results indicate, that upon reaction with the substrate (ethylene, MesCHO or CO) the Al-Si bond order is reduced and both Al and Si oxidized (Table 4). The bond critical points (BCPs) indicate that the interaction between the Al-Si moiety and the substrates is in all three cases non-covalent (closed-shell interactions) in nature (electron density is small and Laplacian positive, compared to the X-Y bond, which is in all cases covalent). QTAIM and NBO are in agreement with these bonding descriptions (although NBO can't find all interactions and only provides dative bonding in many cases, see below).

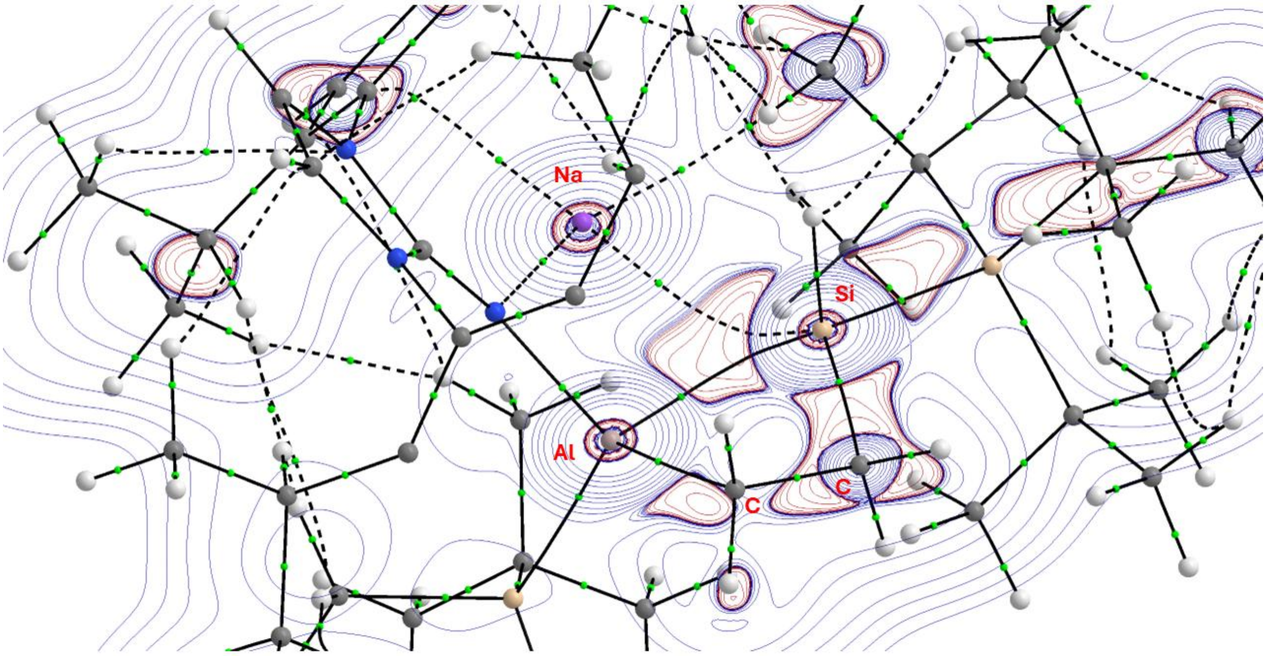

Figure S69: Depiction of the Laplacian of the electron density of **2a** in the Al-Si-C plane. Depicted are areas of charge depletion (blue) and areas of charge concentration (red). Atom colors: N: blue, Al: pink, Si: peach, Na: purple, C: grey and H: white.

Table 4: Details of QTAIM and NBO calculations (values given in a.u.).

Compound	**2a** (X,Y = C)	**5** (X=C, Y=O)	**6** (X=C, Y=O)
BCP (Al-Si)			
ρ	0.0401	0.0485	0.0474
$\nabla^2(\rho)$	0.0257	0.0242	0.0301
ε	0.3944	0.0821	0.0366
BCP (Al-Y)			
ρ	0.0790	0.0776	0.0711
$\nabla^2(\rho)$	0.2247	0.4613	0.4046
ε	0.0512	0.0727	0.0555
BCP (Si-X)			
ρ	0.1099	0.1032	0.1279
$\nabla^2(\rho)$	0.1361	0.1056	0.4299
ε	0.0705	0.1224	0.3269
BCP (X-Y)			
ρ	0.2310	0.2545	0.2934
$\nabla^2(\rho)$	-0.519	-0.5590	-0.5258
ε	0.0457	0.0856	0.0423
**QTAIM** (Bader) charges (Natural charges)			
q(Si)	0.737 (-0.071)	0.690 (0.011)	0.171 (-0.157)
q(Al)	2.177 (1.242)	2.205 (1.373)	2.206 (1.359)
q(X)	-0.694 (-0.829)	-0.102 (-0.299)	-0.382 (-0.394)
q(Y)	-0.700 (-1.035)	-1.356 (-1.047)	-1.373 (-0.981)
**Wiberg bond indices**			
Al-Si	0.6188	0.6757	0.6726
Al-Y	0.5136	0.2688	0.247
Si-X	0.8392	0.7776	1.5292
X-Y	1.0447	0.9344	1.0615

NBO hybridization and localization

If a bond between two atoms is not listed below, the NBO analysis could not identify it (most likely a lone pair donor as a bonding interaction).

2a

Al-Si: Occupancy: 1.90741

22.80% 0.4775*Al $sp^{3.11}$

77.20% 0.8786*Si $sp^{2.07}$

Localization: 22.8% of the bonding electron density originates from Al and 77.2% from Si Hybridization: $sp^{3.11}$ (Al) and $sp^{2.07}$ (Si).

Si-C: Occupancy: 1.96310

68.95% 0.8304*C $sp^{2.59}$

31.05% 0.5572*Si $sp^{3.69}$

5

Al-Si: Occupancy: 1.90865

25.96% 0.5095*Al $sp^{1.93}$

74.04% 0.8605*Si $sp^{2.30}$

C-O: Occupancy: 1.98586

32.24% 0.5851*C $sp^{3.59}$

65.76% 0.8109*O $sp^{2.26}$

6

Al-Si: Occupancy: 1.90115

74.90% 0.8655*Si $sp^{1.39}$

25.10% 0.5010*Al $sp^{2.04}$

Si-C: Occupancy: 1.96111

27.68% 0.5261*Si $sp^{3.69}$

72.32% 0.8504*C $sp^{1.09}$

Si-C: Occupancy: 1.90084 (π-bond of the double bond, the above is the σ-bond)

48.25% 0.6946*Si $sp^{99.99}$ (fully p-orbital character)

51.75% 0.7194*C $sp^{99.99}$

C-O: Occupancy: 1.99219

32.35% 0.5688*C $sp^{3.19}$

67.65% 0.8225*O $sp^{1.72}$

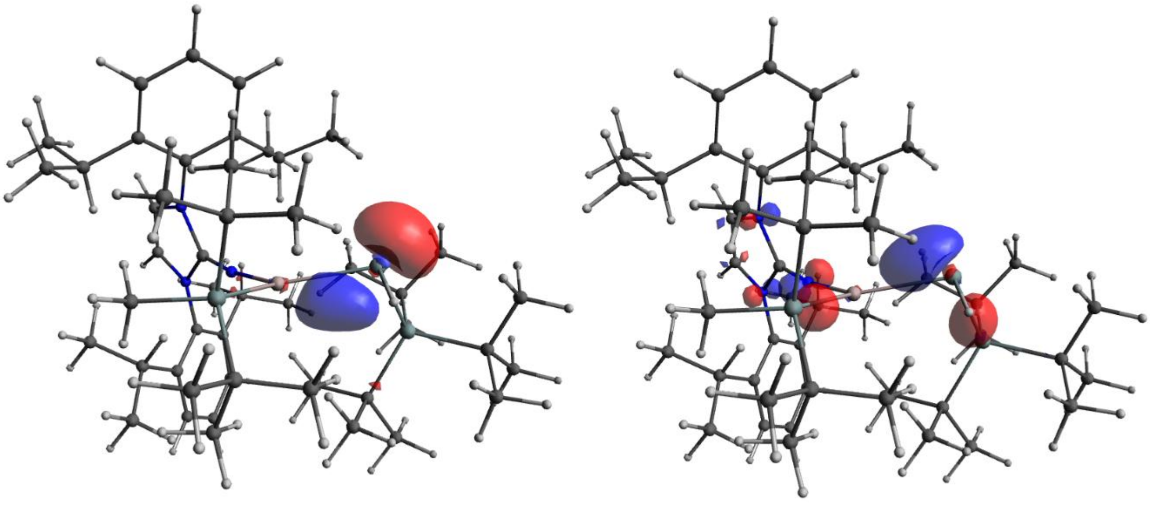

Figure S70. HOMO (left) and HOMO-1 (right) of **1a**. LUMO is delocalized over the aryl ligand.

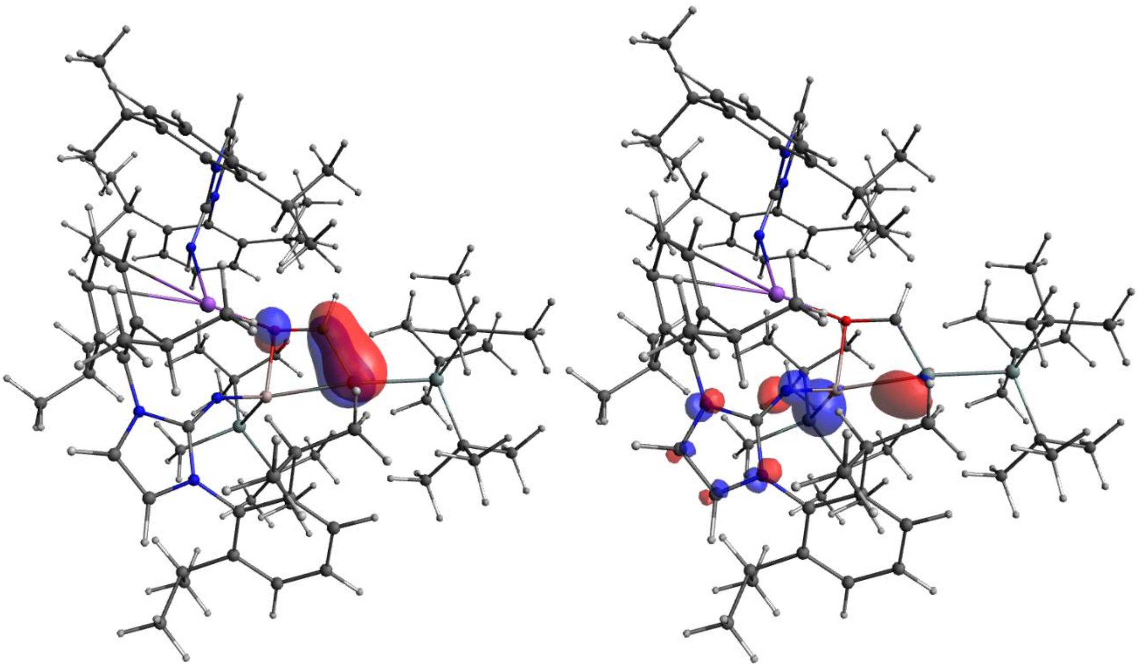

Figure S71. HOMO (left) and HOMO-1 (right) of **6**. LUMO is delocalized over the aryl ligand.

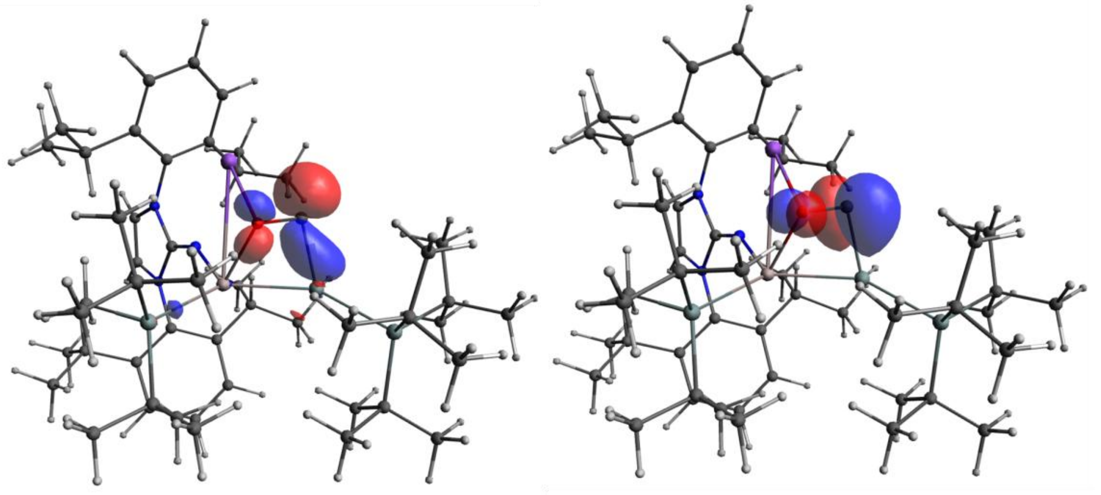

Figure S72: HOMO (left) and LUMO (right) of the carbenic intermediate **Int1** for the calculated mechanism involving **1(Na)** and CO.

Table 5: Energies of the optimized and calculated structures.

Compound	G (Def2-SVP), a.u.	G_{corr} (Def2-SVP), a.u.	E_{sp} (Def2-TZVP), a.u.	E_{sp} + G_{corr} + SMD_{corr}, a.u.
**1Na**	-3312.0673	1.140704	-3315.798849	-3314.658145
**1⁻**	-3149.945671	1.136845	-3153.645458	-3152.508613
**1(DME)$_2$**	-3620.262952	1.265913	-3624.462821	-3623.196908
**2a**	-3390.521676	1.189873	-3394.38537	-3393.195497
**5**	-4082.850614	1.461157	-4087.727339	-4086.266182
**5Na**	-3774.656368	1.324148	-3779.063258	-3777.73911
**6**	-4637.952548	1.704579	-4643.643533	-4641.938954
**6Na**	-3425.231057	1.147291	-3429.09662	-3427.949329
**CO**	-113.110344	-0.01394	-113.22677	-113.24071
**MesCHO**	-462.508028	0.155693	-463.1680571	-463.0123641
Mechanism for **5Na**, TS1	-3774.595944	1.321976	-3778.99854102	-3777.676565
Mechanism for **6Na**, 1Na+CO	-3425.178892	1.140082	-3429.038765	-3427.898683
Mechanism for **6Na**, TS1	-3425.150417	1.143401	-3429.017984	-3427.874583
Mechanism for **6Na**, Int1	-3425.170198	1.144011	-3429.034577	-3427.890566
Mechanism for **6Na**, TS2	-3425.147349	1.140948	-3429.013031	-3427.872083

4.) Supplementary References

[1] M. Ludwig, D. Franz, A. Espinosa Ferao, M. Bolte, F. Hanusch, S. Inoue, *Nat. Chem.* **2023**, *15*, 1452-1460.

[2] N. Kuhn, T. Kratz, *Synthesis* **1993**, 561-562.

[3] Bruker, APEX suite of crystallographic software, (2015.5-2), Madison, Wisconsin, USA, **2015**.

[4] Bruker, SAINT, (Version 8.40A), Madison, Wisconsin, USA, **2016**.

[5] Bruker, SADABS, (Version 2016/2,), Madison, Wisconsin, USA, **2016**.

[6] G. M. Sheldrick, *Acta Crystallogr. C.* **2015**, *71*, 3-8.

[7] G. M. Sheldrick, *Acta Crystallographica Section A: Foundations of Crystallography* **2015**, *71*, 3-8.

[8] C. B. Huebschle, G. M. Sheldrick, B. Dittrich, *J. Appl. Cryst.* **2011**, *44*, 1281.

[9] A. Spek, *Acta Crystallographica Section C* **2015**, *71*, 9-18.

[10] G. M. Sheldrick, SHELXL-2014, Göttingen, Germany, **2014**.

[11] A. J. C. Wilson, V. Geist, *Vol. C*, Kluwer Academic Publishers (published for the International Union of Crystallography), Dordrecht/Boston/London, **1992**, pp. Tables 6.1.1.4 (pp 500-502), 504.502.506.508 (pp. 219-222) and 504.502.504.502 (pp. 193-199).

[12] A. L. Spek, PLATON, A Multipurpose Crystallographic Tool, Utrecht, Netherlands, **2010**.

[13] C. F. Macrae, I. J. Bruno, J. A. Chisholm, P. R. Edgington, P. McCabe, E. Pidcock, L. Rodriguez-Monge, R. Taylor, J. van de Streek, P. A. Wood, *J. Appl. Crystallogr.* **2008**, *41*, 466-470.

[14] D. Kratzert, FinalCif, (V145), Available online: https://dkratzert.de/finalcif.html, Freiburg, **2025**.

[15] M. Frisch, G. Trucks, H. Schlegel, G. Scuseria, M. Robb, J. Cheeseman, G. Scalmani, V. Barone, G. Petersson, H. Nakatsuji, *Wallingford CT*.

[16] J. P. Perdew, K. Burke, M. Ernzerhof, *Phys. Rev. Lett.* **1996**, *77*, 3765.

[17] J. P. Perdew, K. Burke, M. Ernzerhof, *Phys. Rev. Lett.* **1997**, *78*, 1396.

[18] C. Adamo, V. Barone, *J. Chem. Phys.* **1999**, *110*, 6158-6170.

[19] A. Schäfer, H. Horn, R. Ahlrichs, *J. Chem. Phys.* **1992**, *97*, 2571.

[20] A. Schäfer, C. Huber, R. Ahlrichs, *J. Chem. Phys.* **1994**, *100*, 5829-5835.

[21] S. Grimme, S. Ehrlich, L. Goerigk, *J. Comp. Chem.* **2011**, *32*, 1456-1465.

[22] A. V. Marenich, C. J. Cramer, D. G. Truhlar, *J. Phys. Chem.* **2009**, *113*, 6378-6396.

[23] T. A. Keith, *AIMAll (Version 19.10.12), TK Gristmill Software: Overland Park, KS, USA* **2019**, *23*.
